# Probing Evolutionary Patterns in Neotropical Birds through DNA Barcodes

**DOI:** 10.1371/journal.pone.0004379

**Published:** 2009-02-05

**Authors:** Kevin C. R. Kerr, Darío A. Lijtmaer, Ana S. Barreira, Paul D. N. Hebert, Pablo L. Tubaro

**Affiliations:** 1 Department of Integrative Biology, Biodiversity Institute of Ontario, University of Guelph, Guelph, Ontario, Canada; 2 División Ornitología, Museo Argentino de Ciencias Naturales “Bernardino Rivadavia”, Buenos Aires, Argentina; American Museum of Natural History, United States of America

## Abstract

**Background:**

The Neotropical avifauna is more diverse than that of any other biogeographic region, but our understanding of patterns of regional divergence is limited. Critical examination of this issue is currently constrained by the limited genetic information available. This study begins to address this gap by assembling a library of mitochondrial COI sequences, or DNA barcodes, for Argentinian birds and comparing their patterns of genetic diversity to those of North American birds.

**Methodology and Principal Findings:**

Five hundred Argentinian species were examined, making this the first major examination of DNA barcodes for South American birds. Our results indicate that most southern Neotropical bird species show deep sequence divergence from their nearest-neighbour, corroborating that the high diversity of this fauna is not based on an elevated incidence of young species radiations. Although species ages appear similar in temperate North and South American avifaunas, patterns of regional divergence are more complex in the Neotropics, suggesting that the high diversity of the Neotropical avifauna has been fueled by greater opportunities for regional divergence. Deep genetic splits were observed in at least 21 species, though distribution patterns of these lineages were variable. The lack of shared polymorphisms in species, even in species with less than 0.5M years of reproductive isolation, further suggests that selective sweeps could regularly excise ancestral mitochondrial polymorphisms.

**Conclusions:**

These findings confirm the efficacy of species delimitation in birds via DNA barcodes, even when tested on a global scale. Further, they demonstrate how large libraries of a standardized gene region provide insight into evolutionary processes.

## Introduction

DNA barcoding, the survey of sequence diversity in a standard gene region (5′ segment of mitochondrial cytochrome *c* oxidase I, or COI, for animals), has a strong track record for identifying species in varied taxonomic groups [1]–[3]. One particularly comprehensive study of DNA barcodes revealed that 94% of 643 North American bird species possess diagnostic barcode sequences [4], [5]. Moreover, the few cases where barcode sharing limited taxonomic resolution in this fauna involved closely allied species that often hybridize. Similar results have been obtained from the Palearctic; Yoo et al. [6] reported that barcodes reliably identify Korean birds (92 of 450 species were examined).

There remains a need for similar investigations in the Neotropics, the hotspot for avian diversity with a fauna of 3,370 breeding species including 3,121 endemics [7]. Aside from this high taxon diversity, tropical species often possess greater genetic structure than their temperate zone counterparts [8]–[11]. For both these reasons, it has been argued that the Neotropical avifauna will challenge DNA barcoding [12]. Yet, the only previous test of barcoding in Neotropical birds disagreed with this conclusion as it found that 16 species of the endemic family Thamnophilidae could be discriminated [13]. Clearly, a larger-scale investigation is needed. Moreover, a broad survey of sequence diversity at COI in Neotropical birds permits the analysis of patterns of genetic divergence and geographic distributions of distinct lineages, as well as comparisons with other geographic areas (particularly the Nearctic, where most avian species have already been barcoded; [4], [5]). This contribution would be highly valuable to study diverse aspects of evolution in birds and to detect species, or groups of species, requiring more detailed investigations of taxonomic status.

The Argentinian avifauna includes 980 species, approximately 25% of Neotropical bird species [14], [15]. The present study examines patterns of barcode divergence in over half of the bird species native to Argentina. In addition to testing the effectiveness of DNA barcodes for species identification, we explore cases where different species share COI sequences and those where single species include two or more divergent lineages. Finally, and more critically, we analyze patterns of sequence divergence in the birds of Argentina and compare them with those in North America with a view towards understanding the origins of the diversity in South American avifauna and obtaining a broader perspective on mitochondrial genetic variation in New World birds.

## Results

In total, 1,594 sequences were obtained from 500 species representing 51% of the bird species known from Argentina, including 22 of 23 orders and 68 of 81 families (Table S1 provides a species list). On average, 3.2 individuals (range 1–19) were analyzed per species, with 389 taxa represented by multiple specimens. Only sequences longer than 550 bp with less than 1% ambiguous base calls were included (average sequence length was 692 bp), except four sequences possessing 1–3% ambiguous calls that represented the sole records for their species.

**Figure 1:**
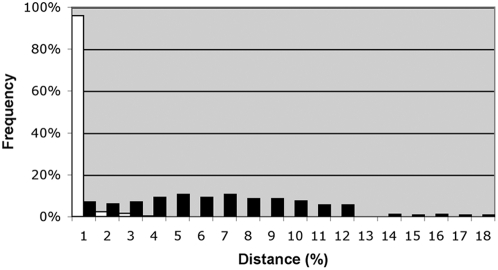
Frequency histogram of COI sequence variation for birds of Argentina. Distance to nearest congeneric neighbour for 282 species from genera represented by multiple taxa (black) and mean intraspecific distance for the 389 species of birds with two or more sequence records (white).

The mean sequence distance among congeneric taxa was 7.6%, while the distance to the nearest congener averaged 6.2%, based on 282 comparisons. In genera represented by multiple species, 92% of species were more than 1.0% divergent from their nearest congener and 83% were more than 2.5% divergent (Figure 1). COI delivered a species identification for 98.8% of species, using either a distance-based criterion or, in cases of very low divergence (less than 0.5%), using diagnostic nucleotide substitutions. Mean intraspecific distance was 0.24% based on the 389 species represented by multiple specimens (weighted equally regardless of the number of individuals). There was a weak association between intraspecific variation and sample size (linear regression, p<0.01, R^2^ = 0.12).

A few cases of low (<1%) divergence between congeners were detected. In most cases, discrimination by COI was possible because each species formed a distinct cluster in a neighbour-joining tree or showed diagnostic sequence differences. For example, sequences of the mockingbird species *Mimus dorsalis* and *M. triurus* showed three diagnostic substitutions, and the woodpeckers *Veniliornis frontalis* and *V. passerinus* differed by two nucleotides. Two goldfinch species (*Carduelis atrata* and *C. crassirostris*) and three ground-tyrants (*Muscisaxicola capistratus*, *M. frontalis* and *M. maclovianus*) also showed low divergences but were separable. Two ducks, *Anas puna* and *A. versicolor,* possessed five diagnostic substitutions, although the latter species showed considerable within species variation at other nucleotide positions. However, in all these cases, barcodes delivered reliable identifications because sequences for each species formed a single cluster (bootstrap support exceeded 70% in all cases, with the exception of *A. versicolor*, which had 57% support). Three other ground-tyrants (*Muscisaxicola flavinucha*, *M. cinereus*, *M. rufivertex*) with even lower genetic divergences were paraphyletic, impeding straightforward identification. Additionally, there was one species complex where barcode resolution was clearly compromised- six species of *Sporophila* (*S. cinnamomea*, *S. hypochroma*, *S. hypoxantha*, *S. palustris*, *S. ruficollis*, *S. zelichi*) all shared barcodes.

Hebert et al. [4] suggested that a sequence threshold of ten times the average intraspecific variation could be used to identify those cases where a current species might represent more than one taxon. For the birds of Argentina, this threshold lies at 2.4% and its application flags 13 species as possessing unusually high sequence variation (Table 1). Another way of identifying species in need of taxonomic scrutiny involves the search for taxa whose specimens form two or more distinct clusters with high bootstrap support (i.e. >98%) in a neighbour-joining tree. If applied to Argentinian birds, eight more species are flagged, all showing maximum intraspecific distances higher than 1.5% (Table 1).

**Table 1:** Bird species from Argentina with two or three deeply divergent groups at COI.

#	Family	Species	Max. distance	Individuals per lineage	Pattern
1	Charadriidae	*Vanellus chilensis*	1.54	4/4	A
2	Strigidae	*Athene cunicularia*	1.60	1/5	A
3	Dendrocolaptidae	***Sittasomus griseicapillus***	**3.25**	**1/6**	A
4	Furnariidae	***Geositta cunicularia***	**3.41**	**1/2**	S
5		***Leptasthenura aegithaloides***	**3.72**	**2/8**	A
6		***Cinclodes fuscus***	**4.65**	**3/5**	A
7		***Upucerthia dumetaria***	**5.41**	**2/10**	A
8		*Cranioleuca pyrrhophya*	1.53	2/4	P
9	Thamnophilidae	***Thamnophilus caerulescens***	**2.44**	**3/4**	P
10		***Thamnophilus ruficapillus***	**4.03**	**2/2**	A
11	Pipridae	***Manacus manacus***	**3.56**	**3/3**	S
12	Tyrannidae	*Serpophaga subcristata*	2.04	2/3	A
13		***Myiophobus fasciatus***	**4.67**	**1/3**	A
14		*Knipolegus aterrimus*	1.9	3/4	P
15	Troglodytidae	***Cistothorus platensis***	**4.95**	**1/2**	A
16		***Troglodytes aedon***	**4.99**	**3/8/8**	A/P
17	Vireonidae	***Vireo olivaceus***	**3.09**	**2/3**	P
18	Thraupidae	***Thraupis bonariensis***	**3.29**	**1/6**	A
19	Cardinalidae	*Cyanocompsa brissonii*	2.04	2/6	P
20		*Saltator aurantiirostris*	1.52	1/6	A
21	Emberizidae	*Arremon flavirostris*	1.75	3/4	A

Species showing more than 2.4% sequence divergence between groups are in bold. Maximum distances are reported in percent divergence. Patterns represent allopatry (A), parapatry (P), or sympatry (S).

More than 10% of the Argentine avifauna (111 of 980 species) also occurs in North America, but 45 of these species are migrants that do not breed in Argentina, five are pelagic visitors to both regions, and six are introduced to one or both areas. However, barcode data are available for 42 of the remaining 55 species, which possess natural breeding ranges extending from Argentina to North America (see Table S2). Seven of these widely distributed species displayed substantial genetic divergence (>2.4%) between North American and Argentinian populations, three displayed smaller divergences (1.5–2.4%), while the remaining 32 showed limited or no divergence.

## Discussion

### Barcodes in Argentinian Birds

Just nine of the 500 bird species included in our study cannot be distinguished using COI sequences. Three of these are *Muscisaxicola* ground-tyrants, which have low interspecific divergence and appear to be paraphyletic. The remaining six species, which all share barcodes, are members of the southern capuchinos, a sub-group within the genus *Sporophila* that includes nine species (seven of them present in Argentina) and shows little mitochondrial sequence variation [16]. Members of this group are believed to have diverged within the past 0.5M years, fueled by sexual selection and a fragmented landscape, and they are known to hybridize [16]. Although shared mitochondrial sequences have also been reported in white-headed gulls [5], in Darwin's finches [17], and possibly in crossbills [18], the present study reinforces earlier evidence that such cases are exceptional in both Nearctic and Neotropical locales despite the known existence of hybridization in birds, which involves around 10% of the world's species [19].

Five additional genera with very low interspecific divergences included a pair or triad of species; none of these cases was a surprise. *Mimus dorsalis* and *M. triurus* are regarded as sister taxa [20], while *Veniliornis frontalis* and *V. passerinus* are so similar morphologically that Nores [21] designated them as allospecies, and Moore et al. [22] suggested that they diverged only 0.35 Mya. *Anas puna* and *A. versicolor* [23] have sometimes been considered conspecific [24], but the present results support the conclusion that they are young species. Arnaiz-Villena et al. [25] suggested a very recent expansion of *Carduelis* in South America to explain the low genetic divergence between *C. atrata* and *C. crassirostris*. Finally, Chesser [26] proposed a middle-late Pleistocene diversification for *Muscisaxicola* because of the shallow divergences between its member species. Further instances of low divergence between species will undoubtedly be revealed as taxonomic coverage builds for Argentina, but there is no evidence that young species radiations are any more common in this region than in North America. Further studies in northern, more tropical areas of South America are needed to establish if this similarity is a consequence of the comparison of two mainly temperate regions in opposite hemispheres or if the same trend is present throughout the Neotropics.

Two evolutionary inferences derive from the present results. First, the relative paucity of very closely related species implies that high species diversity in southern Neotropical birds does not owe its origin to an elevated incidence of young species radiations, a finding that is consistent with recent proposals [27], [28]. Second, and more generally, the low variation within species and the fixation of diagnostic COI sequences even in young species groups conflicts with expectations based on stochastic models of mitochondrial variation, which argue that ancestral polymorphisms will persist for millions of generations [29]. Instead, the rapid emergence of fixed differences is compatible with the growing evidence that selective sweeps recurrently strip variation from mitochondrial gene pools [30], although demographic factors have not been ruled out as a possible explanation.

Tropical taxa are generally thought to show more genetic structure than their temperate zone counterparts, even in the absence of geographical barriers [31]. Work on North American birds revealed deep barcode divergences in 2.7% of species (15/546), all involving allopatric lineages, usually east-west splits. The incidence of deep splits was slightly higher in Argentina with 3.3% of species with multiple records (13/389) showing divergences greater than the 2.4% threshold. Another eight species possessed distinct barcode clusters with 1.5–2.4% divergence, producing a total of 21 species with marked population structure (5.4% of the species examined). Interestingly, these cases of divergence included situations of allopatric, parapatric and sympatric divergence.

**Figure 2:**
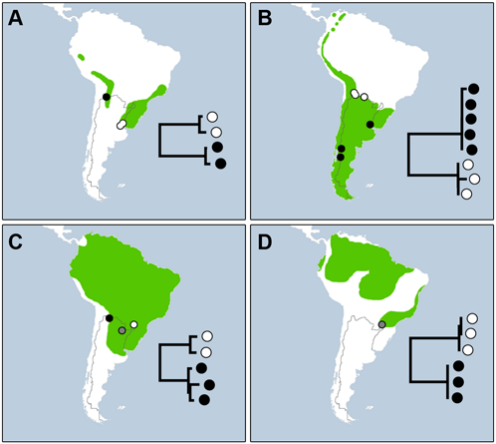
Maps detailing the different distributional patterns of divergent barcode lineages. Species ranges are highlighted in green and circles indicate collection sites. Hollow and filled in circles correspond to lineages represented on superimposed neighbour-joining trees (shaded circles represent sites with overlap). (A) Barcode lineages are allopatric and coincide with disjunctions in the distribution of populations (e.g. *Thamnophilus ruficapillus*). (B) Barcode lineages are allopatric, but species distribution appears continuous (e.g. *Cinclodes fuscus*). (C) Barcode lineages are parapatric (e.g. *Vireo olivaceus*). (D) Barcode lineages are sympatric (e.g. *Manacus manacus*).

Fourteen of the 21 species showed allopatric divergences although there was no simple pattern of geographic structuring. Some cases involved north-south divergence. For example, Patagonian populations of *Cistothorus platensis* and *Cinclodes fuscus* possessed almost 5% divergence from those in northwestern Argentina (Figure 2B), which is consistent with previous findings [32]. Other barcode splits coincided with environmental gradients or known barriers to gene flow. For example, specimens of *Upucerthia dumetaria* from different elevations in the Andes diverged by as much as 5.4%, while 4% divergence between lineages of *Thamnophilus ruficapillus* (see Figure 2A) coincided with isolation caused by the Chaco woodland [21]. Some cases of allopatric divergence seem to represent overlooked species; specimens of *Serpophaga subcristata* from northeastern Argentina and those from Patagonia and Buenos Aires province not only exhibit 2% COI divergence, but differences in morphology and vocalizations [33]. Likewise *Troglodytes aedon* possessed three COI lineages with divergences as high as 5% and its Neotropical populations are thought to include several species (J. Klicka, unpublished data). In other cases, the situation is unclear. For example, two subspecies of *Vanellus chilensis* (northern *V. c. chilensis*, southern *V. c. fretensis*) show just 1.5% divergence, but this matches the divergence between other closely allied species in the same family (e.g. *Charadrius alticola* and *C. falklandicus*).

Five of the 21 species with deep divergences involved cases of parapatry. For example, populations of *Vireo olivaceus* in northeastern and northwestern Argentina possess up to 3.1% sequence divergence, but both COI lineages occurred at one northeastern site (Figure 2C). Does this area represent a region of sympatry between reproductively isolated species or a contact zone between phylogeographic groups? More specimens from this location need to be examined for variation at nuclear loci to resolve this uncertainty [34].

Interestingly, two of the 21 species possessed divergent mitochondrial lineages in sympatry. One of these species, *Manacus manacus,* includes four colour forms that are sometimes regarded as different species [35]. Specimens from Parque Nacional Iguazú included two COI groups with 3.5% divergence and males of both lineages were collected from a single lek (Figure 2D), suggesting that the divergent COI groups in *M. manacus* represent a rare case of deep intra-specific divergence. However, further study is required to determine the origin of such genetic variation and the taxonomic status of this species. Furthermore, this sympatric distribution of lineages could prove to be parapatric with increased sampling (the same could theoretically be true for any of the above examples of allopatry if a region of overlap has been left unsampled). This emphasizes the need to collect several specimens per locality, as well as to sample the entire distribution of each species.

### Barcoding the Avifaunas of Argentina and North America

Although species coverage is higher for North America (643 species, 93% of fauna) than for Argentina (500 species, 51% of fauna), sample sizes are high enough in both regions to provide a good sense of overall patterns of COI variation. Mean intraspecific divergences are congruent - 0.23% in North America and 0.24% in Argentina. The nearest neighbour distance for congeneric taxa is lower in North America (4.3%) than in Argentina (6.2%), but this regional difference will undoubtedly lessen as species coverage builds for Argentina. Barcode sequences are effective for species identification in both settings; 94% of North American birds and 98% of birds from Argentina birds can be identified to a species level. The incidence of deep intraspecific divergences is similar in the two regions (2.7% versus 3.3%), but distributional patterns vary. Most of the genetically divergent groups in North America reflect east-west allopatry [5], while divergences in Argentina are more complex; some are north–south, others are east–west, and yet others occur along altitudinal gradients or in response to specific habitat barriers. Moreover, some cases of deep barcode divergence in Argentinian species involve parapatric or sympatric lineages.

Aside from a test of congruence in barcode patterns, this study provided information on sequence divergences for 42 species whose breeding range extends from Argentina to North America. Fifteen of the 32 species with low divergence are waterbirds (e.g. herons, rails, cormorants, ducks); their use of coastal habitats facilitates gene flow [36]. Seven other species are tropical raptors with limited ranges in North America and whose long-distance movements ensure gene flow [37]. The few small passerines in this group may represent recent range extensions into the southernmost United States. The 10 species with deeper genetic divergences (>1.5%) were largely plain-coloured passerines (*Troglodytes aedon*, *Vireo olivaceus*) and birds with cryptic lifestyles (*Nyctidromus albicollis, Glaucidium brasilianum*). Most possessed a disjunct range, typically with northern migratory and southern non-migratory populations (e.g. *Athene cunicularia, Troglodytes aedon*, *Vireo olivaceus*). Some groups, such as the vireos, are thought to have evolved migratory behaviour on multiple occasions [38], switches that might provoke rapid speciation because they isolate breeding populations [39]. The status of all 10 species with deep splits requires further evaluation, but the need for taxonomic revisions has already been suggested in some cases (e.g., ref. [40]).

Aside from revealing cases of geographic divergence, the coupling of North American and Argentinian results revealed two cases of barcode sharing. *Parula americana*, a species ranging from eastern North America to Central America, shares barcodes with *P. pitiayumi*, a tropical species whose range extends north to Texas. A recent range expansion from a common ancestor has been proposed as the most likely cause for the low divergence between these species [41]. The second case of sequence overlap involves *Anas americana,* restricted to North/Central America, and *A. sibilatrix,* confined to the southern cone of South America. While ducks generally exhibit low genetic divergences, these two species possess striking plumage differences. Peters et al. [42] proposed that rapid phenotypic changes have been provoked by divergent selective pressures in the northern and southern hemispheres.

### Conclusions

The taxonomy of Neotropical birds remains largely reliant on dated morphological studies [43], but molecular data promise to expedite a newly detailed understanding of this fauna [44]. Although levels of genetic differentiation do not dictate taxonomic status [45], barcode analysis illuminates those taxa and those segments of their ranges where further research is justified. Taxonomic decisions cannot be based simply on COI sequences, but barcode surveys are a powerful tool for rapidly identifying those species in need of further investigation. The occurrence of limited variation between well-known sister taxa suggests that even more cryptic species may persist than a liberal thresholding approach, such as the 10× rule, might indicate [46]. The present study shows the way in which a broad-ranging analysis of sequence diversity in a single gene region can also deliver insights concerning the diversification of faunas as opposed to small groups of species. Interestingly, Argentinian and North American birds showed similar incidences of deep intraspecific divergences and of barcode sharing. The underlying causes of both these situations are of great importance to our understanding of avian speciation. We expect that follow-up investigations of sequence variation at other loci, and studies on morphology, behaviour, vocalization and distributions (e.g., ref. [47]) will rapidly advance understanding of the diversity and diversification of the Neotropical avifauna.

## Materials and Methods

Most specimens (88%) were collected by the Ornithology Division of the Museo Argentino de Ciencias Naturales “Bernardino Rivadavia” (MACN) between 2003 and 2007, sometimes in collaboration with other institutions. A few additional specimens were donated (6%), confiscated from illegal traders (4%), or obtained from skins of birds collected after 1995 (2%). DNA was usually extracted from frozen samples of pectoral muscle, liver or heart, but a few extractions were from blood (1%) or small pieces of skin/toe pads from museum skins (2%). All samples derive from the tissue collection at the MACN.

A voucher is present in the MACN or in another collaborating institution for 99% of the specimens that provided a tissue sample for analysis. While most of these vouchers were study skins, a few were skeletons or specimens in ethanol. In the case of blood samples, birds were photographed prior to release to provide an e-voucher. All specimens were identified in the field and validated after preparation; taxonomic assignments follow Clements [48]. Only specimens with confirmed species identities were included. Adults were preferred over juveniles and, in the case of species with sexual dimorphism, males were chosen over females. Specimens were examined from all localities with representatives of a species, but no more than three individuals were analyzed from a single location, excepting a few species with particularly high genetic divergence.

DNA extracts were obtained using glass fibre columns with vertebrate lysis buffer, and an automated protocol using a Biomek FXP liquid handler [49]. Extracts were eluted in 50 µl of molecular grade water. COI sequences were amplified using the primer pair of BirdF1 (5′-TTCTCCAACCACAAAGACATTGGCAC-3′) and COIbirdR2 (5′-ACGTGGGAGATAATTCCAAATCCTGG-3′). When PCR failed and degraded DNA was the suspected cause, internal primers were used in conjunction with those above: AvMiR1 (5′-ACTGAAGCTCCGGCATGGGC-3′) and AvMiF1 (5′-CCCCCGACATAGCATTCC-3′). PCR reactions were initially run following the thermal cycling program in Kerr et al. [5]. Later samples used a shorter program which was equally effective: One cycle at 94°C for 1 min, five cycles of 94°C for 1 min, 45°C for 40 s, and 72°C for 1 min, 35 cycles of 94°C for 1 min, 51°C for 40 s, and 72°C for 1 min, and lastly one cycle of 72°C for 5 min. PCR products were visualized on 2% agarose gels (E-gel 96, Invitrogen) and were bi-directionally sequenced on an ABI 3730×l DNA Analyzer. Sequence records were assembled from forward and reverse reads using SEQUENCHER, version 4.5 (Gene Codes) and aligned by eye using BioEdit version 7.0.5.3 [50].

Specimen and collection data, sequences, and trace files are available in the project ‘Birds of Argentina–Phase I’ in BOLD (http://www.barcodinglife.org). Sequences have been deposited in GenBank under accession numbers FJ027014–FJ028607. BOLD process IDs, museum numbers, and GenBank accession numbers for each specimen analyzed are outlined in Table S3. Comparisons to COI sequences for North American birds employed data available from BOLD in the container project ‘Birds of North America–Phase II’ (sequences are also available from GenBank under accession numbers AY666171–AY666596, DQ432694–DQ433261, DQ433274–DQ433846, and DQ434243–DQ434805).

Sequences were compared using the Kimura 2-parameter distance option [51] in the BOLD Management & Analysis System [52]. Linear regression was performed using R version 2.5.0 [53]. Intra- and interspecific variation were examined visually with neighbour-joining trees generated using the ‘Taxon ID Tree’ option on BOLD. Bootstrap support using 1000 replicates was calculated using MEGA version 4.0 [54]. Sequences of pairs or trios of species with low divergence were analyzed by eye for the identification of diagnostic nucleotides (positions fixed within each species but different between them), which have previously proven to be robust in other species (Kerr et al., unpublished data).

## Supporting Information

Table S1 List of species included in the study (with common English and Spanish names provided), plus the number of individuals analyzed for each.
(0.60 MB DOC)

Table S2 List of species with natural breeding ranges extending from North America to Argentina. The number of individuals sampled per continent is provided, plus mean genetic distance when applicable.
(0.11 MB DOC)

Table S3 List of all specimens included in the study and their associated BOLD process IDs, museum numbers, and GenBank accession numbers.
(1.74 MB DOC)
