# Supplementary material for: Probing Evolutionary Patterns in Neotropical Birds through DNA Barcodes
Source: PLoS One. 2009 Feb 5;4(2):e4379. doi: 10.1371/journal.pone.0004379 (PMC2632745; doi:10.1371/journal.pone.0004379)
Supplement: Table S1 — List of species included in the study (with common English and Spanish names provided), plus the number of individuals analyzed for each. (0.60 MB DOC) [file pone.0004379.s001.doc]

Table S1.

| # | **Species** | **Common Name** | **Nombre Común** | **n** |
| --- | --- | --- | --- | --- |
| 1 | *Crypturellus tataupa* | Tataupa Tinamou | Tataupá Común | 1 |
| 2 | *Rhynchotus rufescens* | Red-winged Tinamou | Colorada | 4 |
| 3 | *Nothoprocta cinerascens* | Brushland Tinamou | Inambú Montarás | 1 |
| 4 | *Nothura darwinii* | Darwin's Nothura | Inambú Pálido | 1 |
| 5 | *Nothura maculosa* | Spotted Nothura | Inambú Común | 4 |
| 6 | *Eudromia elegans* | Elegant Crested Tinamou | Martineta Común | 3 |
| 7 | *Tinamotis pentlandii* | Puna Tinamou | Quiula Puneña | 3 |
| 8 | *Tinamotis ingoufi* | Patagonian Tinamou | Quiula Patagónica | 2 |
| 9 | *Pygoscelis papua* | Gentoo Penguin | Pingüino de Vincha | 1 |
| 10 | *Spheniscus magellanicus* | Magellanic Penguin | Pingüino Patagónico | 3 |
| 11 | *Podilymbus podiceps* | Pied-billed Grebe | Macá Pico Grueso | 1 |
| 12 | *Rollandia rolland* | White-tufted Grebe | Macá Común | 1 |
| 13 | *Podiceps major* | Great Grebe | Macá Grande | 1 |
| 14 | *Podiceps gallardoi* | Hooded Grebe | Macá Tobiano | 2 |
| 15 | *Thalassarche chlororhynchos* | Yellow-nosed Albatross | Albatros Pico Fino | 1 |
| 16 | *Macronectes giganteus* | Antarctic Giant Petrel | Petrel Gigante Común | 1 |
| 17 | *Pelecanoides magellani* | Magellanic Diving-Petrel | Yunco Ceja Blanca | 1 |
| 18 | *Pelecanoides georgicus* | South Georgia Diving-Petrel | Yunco Geórgico | 2 |
| 19 | *Phalacrocorax brasilianus* | Neotropic Cormorant | Biguá | 3 |
| 20 | *Phalacrocorax atriceps* | Imperial Shag | Cormorán Imperial | 2 |
| 21 | *Anhinga anhinga* | Anhinga | Aninga | 1 |
| 22 | *Syrigma sibilatrix* | Whistling Heron | Chiflón | 4 |
| 23 | *Ardea cocoi* | Cocoi Heron | Garza Mora | 2 |
| 24 | *Ardea alba* | Great Egret | Garza Blanca | 3 |
| 25 | *Egretta caerulea* | Little Blue Heron | Garza Azul | 1 |
| 26 | *Egretta thula* | Snowy Egret | Garcita Blanca | 3 |
| 27 | *Bubulcus ibis* | Cattle Egret | Garcita Bueyera | 3 |
| 28 | *Butorides striata* | Striated Heron | Garcita azulada | 3 |
| 29 | *Nycticorax nycticorax* | Black-crowned Night-Heron | Garza Bruja | 1 |
| 30 | *Tigrisoma lineatum* | Rufescent Tiger-Heron | Hocó Colorado | 2 |
| 31 | *Ixobrychus involucris* | Stripe-backed Bittern | Mirasol Común | 1 |
| 32 | *Botaurus pinnatus* | Pinnated Bittern | Mirasol Grande | 1 |
| 33 | *Mycteria americana* | Wood Stork | Tuyuyú | 1 |
| 34 | *Ciconia maguari* | Maguari Stork | Cigüeña Americana | 1 |
| 35 | *Theristicus melanopis* | Black-faced Ibis | Bandurria Austral | 2 |
| 36 | *Phimosus infuscatus* | Bare-faced Ibis | Cuervillo Cara Pelada | 3 |
| 37 | *Plegadis chihi* | White-faced Ibis | Cuervillo de Cañada | 3 |
| 38 | *Platalea ajaja* | Roseate Spoonbill | Espátula Rosada | 2 |
| 39 | *Phoenicopterus chilensis* | Chilean Flamingo | Flamenco Austral | 3 |
| 40 | *Phoenicopterus andinus* | Andean Flamingo | Parina Grande | 2 |
| 41 | *Phoenicopterus jamesi* | Puna Flamingo | Parina Chica | 2 |
| 42 | *Dendrocygna viduata* | White-faced Whistling-Duck | Sirirí Pampa | 7 |
| 43 | *Dendrocygna autumnalis* | Black-bellied Whistling-Duck | Sirirí Vientre Negro | 2 |
| 44 | *Cygnus melancoryphus* | Black-necked Swan | Cisne Cuello Negro | 2 |
| 45 | *Coscoroba coscoroba* | Coscoroba Swan | Coscoroba | 2 |
| 46 | *Chloephaga picta* | Upland Goose | Cauquén Común | 2 |
| 47 | *Chloephaga poliocephala* | Ashy-headed Goose | Cauquén Real | 2 |
| 48 | *Sarkidiornis melanotos* | Comb Duck | Pato Crestudo | 1 |
| 49 | *Callonetta leucophrys* | Ringed Teal | Pato de Collar | 6 |
| 50 | *Amazonetta brasiliensis* | Brazilian Teal | Pato Cutirí | 7 |
| 51 | *Anas sibilatrix* | Chiloe Wigeon | Pato Overo | 6 |
| 52 | *Anas flavirostris* | Speckled Teal | Pato Barcino | 5 |
| 53 | *Anas specularioides* | Crested Duck | Pato Crestón | 5 |
| 54 | *Anas georgica* | Yellow-billed Pintail | Pato Maicero | 6 |
| 55 | *Anas bahamensis* | White-cheeked Pintail | Pato Gargantilla | 1 |
| 56 | *Anas puna* | Puna Teal | Pato Puneño | 2 |
| 57 | *Anas versicolor* | Silver Teal | Pato Capuchino | 8 |
| 58 | *Anas cyanoptera* | Cinnamon Teal | Pato Colorado | 5 |
| 59 | *Anas platalea* | Red Shoveler | Pato Cuchara | 3 |
| 60 | *Netta peposaca* | Rosy-billed Pochard | Pato Picazo | 6 |
| 61 | *Heteronetta atricapilla* | Black-headed Duck | Pato Cabeza Negra | 1 |
| 62 | *Nomonyx dominica* | Masked Duck | Pato Fierro | 1 |
| 63 | *Oxyura ferruginea* | Andean Duck | Pato Zambullidor Grande | 1 |
| 64 | *Coragyps atratus* | Black Vulture | Jote Cabeza Negra | 2 |
| 65 | *Cathartes burrovianus* | Lesser Yellow-headed Vulture | Jote Cabeza Amarilla | 2 |
| 66 | *Gampsonyx swainsonii* | Pearl Kite | Milano Chico | 1 |
| 67 | *Elanus leucurus* | White-tailed Kite | Milano Blanco | 1 |
| 68 | *Rostrhamus sociabilis* | Snail Kite | Caracolero | 2 |
| 69 | *Ictinia mississippiensis* | Mississippi Kite | Milano Boreal | 2 |
| 70 | *Ictinia plumbea* | Plumbeous Kite | Milano Plomizo | 3 |
| 71 | *Circus buffoni* | Long-winged Harrier | Gavilán Planeador | 1 |
| 72 | *Circus cinereus* | Cinereous Harrier | Gavilán Ceniciento | 1 |
| 73 | *Accipiter erythronemius* | Rufous-thighed Hawk | Esparvero Común | 4 |
| 74 | *Accipiter bicolor* | Bicolored Hawk | Esparvero Variado | 3 |
| 75 | *Geranospiza caerulescens* | Crane Hawk | Gavilán Patas Largas | 1 |
| 76 | *Buteogallus urubitinga* | Great Black-Hawk | Águila Negra | 3 |
| 77 | *Buteogallus meridionalis* | Savanna Hawk | Aguilucho Colorado | 3 |
| 78 | *Parabuteo unicinctus* | Harris' Hawk | Gavilán Mixto | 1 |
| 79 | *Busarellus nigricollis* | Black-collared Hawk | Aguilucho Pampa | 1 |
| 80 | *Geranoaetus melanoleucus* | Black-chested Buzzard-Eagle | Águila Mora | 1 |
| 81 | *Buteo magnirostris* | Roadside Hawk | Taguató Común | 6 |
| 82 | *Buteo leucorrhous* | White-rumped Hawk | Taguató Negro | 2 |
| 83 | *Buteo albicaudatus* | White-tailed Hawk | Aguilucho Alas Largas | 1 |
| 84 | *Buteo polyosoma* | Red-backed Hawk | Aguilucho Común | 1 |
| 85 | *Caracara plancus* | Southern Caracara | Carancho | 3 |
| 86 | *Milvago chimachima* | Yellow-headed Caracara | Chimachima | 4 |
| 87 | *Falco sparverius* | American Kestrel | Halconcito Colorado | 3 |
| 88 | *Falco femoralis* | Aplomado Falcon | Halcón Plomizo | 4 |
| 89 | *Falco peregrinus* | Peregrine Falcon | Halcón Pelegrino | 1 |
| 90 | *Callipepla californica* | California Quail | Codorniz de California | 2 |
| 91 | *Aramus guarauna* | Limpkin | Carau | 3 |
| 92 | *Aramides cajanea* | Gray-necked Wood-Rail | Chiricote | 2 |
| 93 | *Aramides ypecaha* | Giant Wood-Rail | Ipacaá | 4 |
| 94 | *Pardirallus sanguinolentus* | Plumbeous Rail | Gallineta Común | 1 |
| 95 | *Porphyrio flavirostris* | Azure Gallinule | Pollona Celeste | 1 |
| 96 | *Gallinula chloropus* | Common Moorhen | Pollona Negra | 3 |
| 97 | *Gallinula melanops* | Spot-flanked Gallinule | Pollona Pintada | 2 |
| 98 | *Fulica ardesiaca* | Slate-colored Coot | Gallareta Andina | 2 |
| 99 | *Fulica armillata* | Red-gartered Coot | Gallareta Ligas Rojas | 4 |
| 100 | *Fulica rufifrons* | Red-fronted Coot | Gallareta Escudete Rojo | 1 |
| 101 | *Fulica gigantea* | Giant Coot | Gallareta Gigante | 1 |
| 102 | *Fulica cornuta* | Horned Coot | Gallareta Cornuda | 1 |
| 103 | *Jacana jacana* | Wattled Jacana | Jacana | 2 |
| 104 | *Rostratula semicollaris* | American Painted-snipe | Aguatero | 3 |
| 105 | *Haematopus leucopodus* | Magellanic Oystercatcher | Ostrero Austral | 1 |
| 106 | *Haematopus ater* | Blackish Oystercatcher | Ostrero Negro | 1 |
| 107 | *Himantopus melanurus* | White-backed Stilt | Tero-real | 3 |
| 108 | *Recurvirostra andina* | Andean Avocet | Avoceta Andina | 4 |
| 109 | *Vanellus chilensis* | Southern Lapwing | Tero Común | 8 |
| 110 | *Pluvialis dominica* | American Golden-Plover | Chorlo Pampa | 1 |
| 111 | *Charadrius collaris* | Collared Plover | Chorlito de Collar | 3 |
| 112 | *Charadrius alticola* | Puna Plover | Chorlito Puneño | 5 |
| 113 | *Charadrius falklandicus* | Two-banded Plover | Chorlito Doble Collar | 2 |
| 114 | *Charadrius modestus* | Rufous-chested Dotterel | Chorlito Pecho Colorado | 2 |
| 115 | *Oreopholus ruficollis* | Tawny-throated Dotterel | Charlo Cabezón | 1 |
| 116 | *Pluvianellus socialis* | Magellanic Plover | Chorlito Ceniciento | 2 |
| 117 | *Gallinago paraguaiae* | South American Snipe | Becasina Común | 3 |
| 118 | *Tringa solitaria* | Solitary Sandpiper | Pitotoy Solitario | 2 |
| 119 | *Tringa melanoleuca* | Greater Yellowlegs | Pitotoy Grande | 1 |
| 120 | *Tringa flavipes* | Lesser Yellowlegs | Pitotoy Chico | 3 |
| 121 | *Calidris bairdii* | Baird's Sandpiper | Playerito Unicolor | 4 |
| 122 | *Calidris melanotos* | Pectoral Sandpiper | Playerito Pectoral | 2 |
| 123 | *Attagis gayi* | Rufous-bellied Seedsnipe | Agachona Grande | 2 |
| 124 | *Attagis malouinus* | White-bellied Seedsnipe | Agachona Patagónica | 1 |
| 125 | *Thinocorus orbignyianus* | Gray-breasted Seedsnipe | Agachona de Collar | 6 |
| 126 | *Larus scoresbii* | Dolphin Gull | Gaviota Gris | 2 |
| 127 | *Larus dominicanus* | Kelp Gull | Gaviota Cocinera | 2 |
| 128 | *Sternula superciliaris* | Yellow-billed Tern | Gaviotín Chico Común | 2 |
| 129 | *Phaetusa simplex* | Large-billed Tern | Atí | 1 |
| 130 | *Sterna trudeaui* | Snowy-crowned Tern | Gaviotín Lagunero | 2 |
| 131 | *Thalasseus maximus* | Royal Tern | Gaviotín Real | 2 |
| 132 | *Thalasseus sandvicensis* | Sandwich Tern | Gaviotín Pico Amarillo | 2 |
| 133 | *Rynchops niger* | Black Skimmer | Rayador | 3 |
| 134 | *Stercorarius antarcticus* | Brown Skua | Escúa Antártica | 1 |
| 135 | *Columba livia* | Rock Pigeon | Paloma Doméstica | 1 |
| 136 | *Patagioenas picazuro* | Picazuro Pigeon | Paloma Picazuró | 6 |
| 137 | *Patagioenas maculosa* | Spot-winged Pigeon | Paloma Manchada | 3 |
| 138 | *Patagioenas araucana* | Chilean Pigeon | Paloma Araucana | 3 |
| 139 | *Patagioenas cayennensis* | Pale-vented Pigeon | Paloma Colorada | 1 |
| 140 | *Zenaida auriculata* | Eared Dove | Torcaza | 8 |
| 141 | *Columbina talpacoti* | Ruddy Ground-Dove | Torcacita Colorada | 4 |
| 142 | *Columbina picui* | Picui Ground-Dove | Torcacita Común | 7 |
| 143 | *Metriopelia melanoptera* | Black-winged Ground-Dove | Palomita Cordillerana | 2 |
| 144 | *Metriopelia aymara* | Golden-spotted Ground-Dove | Palomita Dorada | 5 |
| 145 | *Leptotila verreauxi* | White-tipped Dove | Yerutí Común | 6 |
| 146 | *Leptotila megalura* | White-faced Dove | Yerutí Yungueña | 1 |
| 147 | *Leptotila rufaxilla* | Gray-fronted Dove | Yerutí Colorada | 1 |
| 148 | *Aratinga acuticaudata* | Blue-crowned Parakeet | Calacante Común | 2 |
| 149 | *Aratinga mitrata* | Mitred Parakeet | Calacante Cara Roja | 3 |
| 150 | *Aratinga leucophthalma* | White-eyed Parakeet | Calacante Ala Roja | 2 |
| 151 | *Aratinga aurea* | Peach-fronted Parakeet | Calacante Frente Dorada | 1 |
| 152 | *Cyanoliseus patagonus* | Burrowing Parrot | Loro Barranquero | 3 |
| 153 | *Pyrrhura molinae* | Green-cheeked Parakeet | Chiripepé Cabeza Parda | 2 |
| 154 | *Myiopsitta monachus* | Monk Parakeet | Cotorra | 8 |
| 155 | *Psilopsiagon aymara* | Gray-hooded Parakeet | Catita Serrana Grande | 3 |
| 156 | *Psilopsiagon aurifrons* | Mountain Parakeet | Catita Serrana Chica | 2 |
| 157 | *Forpus xanthopterygius* | Blue-winged Parrotlet | Catita Enana | 2 |
| 158 | *Brotogeris chiriri* | Yellow-chevroned Parakeet | Catita Chirirí | 1 |
| 159 | *Pionus maximiliani* | Scaly-headed Parrot | Loro Mataica | 2 |
| 160 | *Amazona tucumana* | Tucuman Parrot | Loro Alisero | 3 |
| 161 | *Amazona aestiva* | Blue-fronted Parrot | Loro Hablador | 3 |
| 162 | *Coccyzus cinereus* | Ash-colored Cuckoo | Cuclillo Chico | 1 |
| 163 | *Coccyzus melacoryphus* | Dark-billed Cuckoo | Cuclillo Canela | 3 |
| 164 | *Piaya cayana* | Squirrel Cuckoo | Tingazú | 3 |
| 165 | *Crotophaga ani* | Smooth-billed Ani | Anó Chico | 3 |
| 166 | *Guira guira* | Guira Cuckoo | Pirincho | 1 |
| 167 | *Tapera naevia* | Striped Cuckoo | Crespín | 4 |
| 168 | *Tyto alba* | Barn Owl | Lechuza-de-campanario | 1 |
| 169 | *Megascops choliba* | Tropical Screech-Owl | Alilicucu Común | 4 |
| 170 | *Megascops hoyi* | Hoy's Screech-Owl | Alilicucu Grande | 1 |
| 171 | *Bubo magellanicus* | Magellanic Horned Owl | Ñacurutú | 1 |
| 172 | *Strix rufipes* | Rufous-legged Owl | Lechuza Bataraz | 1 |
| 173 | *Glaucidium brasilianum* | Ferruginous Pygmy-Owl | Caburé Chico | 3 |
| 174 | *Glaucidium nanum* | Austral Pygmy-Owl | Caburé Grande | 2 |
| 175 | *Athene cunicularia* | Burrowing Owl | Lechucita Vizcachera | 6 |
| 176 | *Pseudoscops clamator* | Striped Owl | Lechuzón Orejudo | 1 |
| 177 | *Asio flammeus* | Short-eared Owl | Lechuzón de Campo | 1 |
| 178 | *Nyctibius griseus* | Common Potoo | Urutaú Común | 1 |
| 179 | *Podager nacunda* | Nacunda Nighthawk | Ñacundá | 3 |
| 180 | *Nyctidromus albicollis* | Pauraque | Curiango | 1 |
| 181 | *Caprimulgus parvulus* | Little Nightjar | Atajacaminos Chico | 2 |
| 182 | *Hydropsalis torquata* | Scissor-tailed Nightjar | Atajacaminos Tijera Común | 5 |
| 183 | *Eleothreptus anomalus* | Sickle-winged Nightjar | Atajacaminos Ala Negra | 3 |
| 184 | *Chaetura andrei* | Ashy-tailed Swift | Vencejo de Tormenta | 1 |
| 185 | *Phaethornis eurynome* | Scale-throated Hermit | Ermitaño Escamado | 2 |
| 186 | *Colibri coruscans* | Sparkling Violet-ear | Colibrí Grande | 3 |
| 187 | *Stephanoxis lalandi* | Plovercrest | Picaflor Copetón | 1 |
| 188 | *Chlorostilbon aureoventris* | Glittering-bellied Emerald | Picaflor Común | 6 |
| 189 | *Thalurania glaucopis* | Violet-capped Woodnymph | Picaflor Corona Violácea | 3 |
| 190 | *Hylocharis chrysura* | Gilded Sapphire | Picaflor Bronceado | 6 |
| 191 | *Leucippus chionogaster* | White-bellied Hummingbird | Picaflor Vientre Blanco | 4 |
| 192 | *Agyrtria versicolor* | Versicolored Emerald | Picaflor Esmeralda | 2 |
| 193 | *Oreotrochilus leucopleurus* | White-sided Hillstar | Picaflor Andino | 4 |
| 194 | *Patagona gigas* | Giant Hummingbird | Picaflor Gigante | 3 |
| 195 | *Sephanoides sephaniodes* | Green-backed Firecrown | Picaflor Rubí | 3 |
| 196 | *Eriocnemis glaucopoides* | Blue-capped Puffleg | Picaflor Frente Azul | 3 |
| 197 | *Sappho sparganura* | Red-tailed Comet | Picaflor Cometa | 3 |
| 198 | *Trogon rufus* | Black-throated Trogon | Surucuá Amarillo | 1 |
| 199 | *Trogon surrucura* | Surucua Trogon | Surucuá Común | 5 |
| 200 | *Ceryle torquatus* | Ringed Kingfisher | Martín Pescador Grande | 4 |
| 201 | *Chloroceryle amazona* | Amazon Kingfisher | Martín Pescador Mediano | 3 |
| 202 | *Chloroceryle americana* | Green Kingfisher | Martín Pescador Chico | 3 |
| 203 | *Baryphthengus ruficapillus* | Rufous-capped Motmot | Yeruvá | 3 |
| 204 | *Nystalus chacuru* | White-eared Puffbird | Chacurú Cara Negra | 1 |
| 205 | *Nonnula rubecula* | Rusty-breasted Nunlet | Charcurú Chico | 1 |
| 206 | *Pteroglossus castanotis* | Chestnut-eared Araçari | Arasarí Fajado | 2 |
| 207 | *Ramphastos dicolorus* | Red-breasted Toucan | Tucán Pico Verde | 1 |
| 208 | *Ramphastos toco* | Toco Toucan | Tucán Grande | 1 |
| 209 | *Picumnus cirratus* | White-barred Piculet | Carpinterito Común | 6 |
| 210 | *Picumnus temminckii* | Ochre-collared Piculet | Carpinterito Cuello Canela | 2 |
| 211 | *Melanerpes candidus* | White Woodpecker | Carpintero Blanco | 5 |
| 212 | *Melanerpes cactorum* | White-fronted Woodpecker | Carpintero del Cardón | 4 |
| 213 | *Picoides lignarius* | Striped Woodpecker | Carpintero Bataraz Grande | 2 |
| 214 | *Picoides mixtus* | Checkered Woodpecker | Carpintero Bataraz Chico | 6 |
| 215 | *Veniliornis passerinus* | Little Woodpecker | Carpintero Oliva Chico | 3 |
| 216 | *Veniliornis frontalis* | Dot-fronted Woodpecker | Carpintero Oliva Yungueño | 1 |
| 217 | *Piculus chrysochloros* | Golden-green Woodpecker | Carpintero Dorado Común | 3 |
| 218 | *Colaptes melanochloros* | Green-barred Woodpecker | Carpintero Real | 6 |
| 219 | *Colaptes pitius* | Chilean Flicker | Carpintero Pitío | 3 |
| 220 | *Colaptes rupicola* | Andean Flicker | Carpintero Andino | 1 |
| 221 | *Colaptes campestris* | Campo Flicker | Carpintero Campestre | 2 |
| 222 | *Celeus lugubris* | Pale-crested Woodpecker | Carpintero Copete Pajizo | 3 |
| 223 | *Dryocopus lineatus* | Lineated Woodpecker | Carpintero Garganta Estriada | 1 |
| 224 | *Campephilus leucopogon* | Cream-backed Woodpecker | Carpintero Lomo Blanco | 6 |
| 225 | *Campephilus magellanicus* | Magellanic Woodpecker | Carpintero Gigante | 3 |
| 226 | *Geositta cunicularia* | Common Miner | Caminera Común | 3 |
| 227 | *Geositta punensis* | Puna Miner | Caminera Puneña | 5 |
| 228 | *Upucerthia dumetaria* | Scale-throated Earthcreeper | Bandurrita Común | 12 |
| 229 | *Upucerthia ruficaudus* | Straight-billed Earthcreeper | Bandurrita Pico Recto | 3 |
| 230 | *Ochetorhynchus certhioides* | Chaco Earthcreeper | Bandurrita Chaqueña | 5 |
| 231 | *Eremobius phoenicurus* | Band-tailed Earthcreeper | Bandurrita Patagónica | 6 |
| 232 | *Cinclodes fuscus* | Bar-winged Cinclodes | Remolinera Común | 8 |
| 233 | *Cinclodes patagonicus* | Dark-bellied Cinclodes | Remolinera Araucana | 4 |
| 234 | *Cinclodes atacamensis* | White-winged Cinclodes | Remolinera Castaña | 3 |
| 235 | *Furnarius rufus* | Rufous Hornero | Hornero | 10 |
| 236 | *Phleocryptes melanops* | Wren-like Rushbird | Junquero | 5 |
| 237 | *Aphrastura spinicauda* | Thorn-tailed Rayadito | Rayadito | 6 |
| 238 | *Leptasthenura fuliginiceps* | Brown-capped Tit-Spinetail | Coludito Canela | 3 |
| 239 | *Leptasthenura platensis* | Tufted Tit-Spinetail | Coludito Copetón | 1 |
| 240 | *Leptasthenura aegithaloides* | Plain-mantled Tit-Spinetail | Coludito Cola Negra | 10 |
| 241 | *Spartonoica maluroides* | Bay-capped Wren-Spinetail | Espartillero Enano | 1 |
| 242 | *Sylviorthorhynchus desmursii* | Des Murs' Wiretail | Colilarga | 3 |
| 243 | *Schoeniophylax phryganophilus* | Chotoy Spinetail | Chotoy | 4 |
| 244 | *Synallaxis frontalis* | Sooty-fronted Spinetail | Pijuí Frente Gris | 4 |
| 245 | *Synallaxis azarae* | Azara's Spinetail | Pijuí Ceja Canela | 3 |
| 246 | *Synallaxis albescens* | Pale-breasted Spinetail | Pijuí Cola Parda | 6 |
| 247 | *Synallaxis spixi* | Chicli Spinetail | Pijuí Plomizo | 2 |
| 248 | *Cranioleuca pyrrhophia* | Stripe-crowned Spinetail | Curutié Blanco | 6 |
| 249 | *Certhiaxis cinnamomeus* | Yellow-chinned Spinetail | Curutié Colorado | 6 |
| 250 | *Asthenes modesta* | Cordilleran Canastero | Canastero Pálido | 4 |
| 251 | *Asthenes anthoides* | Austral Canastero | Espartillero Austral | 3 |
| 252 | *Asthenes pyrrholeuca* | Lesser Canastero | Canastero Coludo | 9 |
| 253 | *Asthenes dorbignyi* | Creamy-breasted Canastero | Canastero Rojizo | 3 |
| 254 | *Asthenes steinbachi* | Steinbach's Canastero | Canastero Castaño | 1 |
| 255 | *Asthenes baeri* | Short-billed Canastero | Canastero Chaqueño | 5 |
| 256 | *Asthenes patagonica* | Patagonian Canastero | Canastero Patagónico | 3 |
| 257 | *Phacellodomus rufifrons* | Common Thornbird | Espinero Frente Rojiza | 1 |
| 258 | *Phacellodomus striaticeps* | Streak-fronted Thornbird | Espinero Andino | 1 |
| 259 | *Phacellodomus striaticollis* | Freckle-breasted Thornbird | Espinero Pecho Manchado | 3 |
| 260 | *Phacellodomus ruber* | Greater Thornbird | Espinero Grande | 8 |
| 261 | *Phacellodomus maculipectus* | Spot-breasted Thornbird | Espinero Serrano | 1 |
| 262 | *Anumbius annumbi* | Firewood-gatherer | Leñatero | 4 |
| 263 | *Coryphistera alaudina* | Lark-like Brushrunner | Crestudo | 3 |
| 264 | *Pseudoseisura gutturalis* | White-throated Cacholote | Cachalote Pardo | 5 |
| 265 | *Syndactyla rufosuperciliata* | Buff-browed Foliage-gleaner | Ticotico Común | 5 |
| 266 | *Philydor lichtensteini* | Ochre-breasted Foliage-gleaner | Ticotico Ocráceo | 1 |
| 267 | *Philydor atricapillus* | Black-capped Foliage-gleaner | Ticotico Cabeza Negra | 2 |
| 268 | *Automolus leucophthalmus* | White-eyed Foliage-gleaner | Ticotico Ojo Blanco | 4 |
| 269 | *Xenops minutus* | Plain Xenops | Picolezna Chico | 3 |
| 270 | *Pygarrhichas albogularis* | White-throated Treerunner | Picolezna Patagónico | 5 |
| 271 | *Dendrocincla turdina* | Thrush-like Woodcreeper | Arapasú | 3 |
| 272 | *Sittasomus griseicapillus* | Olivaceous Woodcreeper | Tarefero | 7 |
| 273 | *Xiphocolaptes albicollis* | White-throated Woodcreeper | Trepador Garganta Blanca | 2 |
| 274 | *Xiphocolaptes major* | Great Rufous Woodcreeper | Trepador Gigante | 3 |
| 275 | *Dendrocolaptes platyrostris* | Planalto Woodcreeper | Trepador Oscuro | 2 |
| 276 | *Xiphorhynchus fuscus* | Lesser Woodcreeper | Chinchero Enano | 3 |
| 277 | *Lepidocolaptes angustirostris* | Narrow-billed Woodcreeper | Chinchero Chico | 7 |
| 278 | *Lepidocolaptes falcinellus* | Scalloped Woodcreeper | Chinchero Escamado | 1 |
| 279 | *Campylorhamphus trochilirostris* | Red-billed Scythebill | Picapalo Colorado | 3 |
| 280 | *Taraba major* | Great Antshrike | Chororó | 3 |
| 281 | *Thamnophilus ruficapillus* | Rufous-capped Antshrike | Choca Corana Rojiza | 4 |
| 282 | *Thamnophilus caerulescens* | Variable Antshrike | Choca Común | 7 |
| 283 | *Dysithamnus mentalis* | Plain Antvireo | Choca Amarilla | 3 |
| 284 | *Drymophila rubricollis* | Bertoni's Antbird | Tiluchi Colorado | 1 |
| 285 | *Pyriglena leucoptera* | White-shouldered Fire-eye | Batará Negro | 3 |
| 286 | *Grallaria albigula* | White-throated Antpitta | Chululú Cabeza Rojiza | 2 |
| 287 | *Conopophaga lineata* | Rufous Gnateater | Chupadientes | 1 |
| 288 | *Pteroptochos tarnii* | Black-throated Huet-huet | Huet-huet | 1 |
| 289 | *Scelorchilus rubecula* | Chucao Tapaculo | Chucao | 2 |
| 290 | *Scytalopus superciliaris* | White-browed Tapaculo | Churrín Ceja Blanca | 3 |
| 291 | *Scytalopus magellanicus* | Magellanic Tapaculo | Churrín Andino | 3 |
| 292 | *Phytotoma rutila* | White-tipped Plantcutter | Cortarramas | 7 |
| 293 | *Phytotoma rara* | Rufous-tailed Plantcutter | Rara | 3 |
| 294 | *Pipra fasciicauda* | Band-tailed Manakin | Bailarín Naranja | 3 |
| 295 | *Chiroxiphia caudata* | Blue Manakin | Bailarín Azul | 3 |
| 296 | *Manacus manacus* | White-bearded Manakin | Bailarín Blanco | 6 |
| 297 | *Schiffornis virescens* | Greenish Schiffornis | Bailarín Oliváceo | 3 |
| 298 | *Phyllomyias sclateri* | Sclater's Tyrannulet | Mosqueta Corona Gris | 3 |
| 299 | *Myiopagis viridicata* | Greenish Elaenia | Fiofío Corona Dorada | 1 |
| 300 | *Elaenia spectabilis* | Large Elaenia | Fiofío Grande | 4 |
| 301 | *Elaenia albiceps* | White-crested Elaenia | Fiofío Silbón | 4 |
| 302 | *Elaenia parvirostris* | Small-billed Elaenia | Fiofío Pico Corto | 4 |
| 303 | *Elaenia strepera* | Slaty Elaenia | Fiofío Plomizo | 3 |
| 304 | *Elaenia obscura* | Highland Elaenia | Fiofío Oscuro | 1 |
| 305 | *Camptostoma obsoletum* | Southern Beardless-Tyrannulet | Piojito Silbón | 2 |
| 306 | *Suiriri suiriri* | Suiriri Flycatcher | Suirirí Común | 3 |
| 307 | *Mecocerculus leucophrys* | White-throated Tyrannulet | Piojito Gargantilla | 3 |
| 308 | *Anairetes flavirostris* | Yellow-billed Tit-Tyrant | Cachudito Pico Amarillo | 3 |
| 309 | *Anairetes parulus* | Tufted Tit-Tyrant | Cachudito Pico Negro | 4 |
| 310 | *Serpophaga subcristata* | White-crested Tyrannulet | Piojito Común | 5 |
| 311 | *Phaeomyias murina* | Mouse-colored Tyrannulet | Piojito Pardo | 2 |
| 312 | *Pseudocolopteryx sclateri* | Crested Doradito | Doradito Copetón | 2 |
| 313 | *Pseudocolopteryx flaviventris* | Warbling Doradito | Doradito Común | 1 |
| 314 | *Corythopis delalandi* | Southern Antpipit | Mosquitero | 3 |
| 315 | *Euscarthmus meloryphus* | Tawny-crowned Pygmy-Tyrant | Barullero | 1 |
| 316 | *Stigmatura budytoides* | Greater Wagtail-Tyrant | Calandrita | 3 |
| 317 | *Phylloscartes ventralis* | Mottle-cheeked Tyrannulet | Mosqueta Común | 2 |
| 318 | *Leptopogon amaurocephalus* | Sepia-capped Flycatcher | Mosqueta Corona Parda | 6 |
| 319 | *Mionectes rufiventris* | Gray-hooded Flycatcher | Ladrillito | 4 |
| 320 | *Sublegatus modestus* | Southern Scrub-Flycatcher | Suirirí Pico Corto | 2 |
| 321 | *Tachuris rubrigastra* | Many-colored Rush-Tyrant | Tachurí Sietecolores | 3 |
| 322 | *Hemitriccus diops* | Drab-breasted Bamboo-Tyrant | Mosqueta de Anteojos | 1 |
| 323 | *Hemitriccus margaritaceiventer* | Pearly-vented Tody-Tyrant | Mosqueta Ojo Dorado | 5 |
| 324 | *Platyrinchus mystaceus* | White-throated Spadebill | Picochato Enano | 3 |
| 325 | *Myiophobus fasciatus* | Bran-colored Flycatcher | Mosqueta Estriada | 4 |
| 326 | *Pyrrhomyias cinnamomeus* | Cinnamon Flycatcher | Birro Chico | 1 |
| 327 | *Lathrotriccus euleri* | Euler's Flycatcher | Mosqueta Parda | 3 |
| 328 | *Cnemotriccus fuscatus* | Fuscous Flycatcher | Mosqueta Ceja Blanca | 1 |
| 329 | *Contopus fumigatus* | Smoke-colored Pewee | Burlisto Copetón | 1 |
| 330 | *Pyrocephalus rubinus* | Vermilion Flycatcher | Churrinche | 6 |
| 331 | *Lessonia rufa* | Austral Negrito | Sobrepuesto Común | 6 |
| 332 | *Lessonia oreas* | Andean Negrito | Sobrepuesto Andino | 6 |
| 333 | *Hymenops perspicillatus* | Spectacled Tyrant | Pico de Plata | 7 |
| 334 | *Knipolegus striaticeps* | Cinereous Tyrant | Viudita Chaqueña | 1 |
| 335 | *Knipolegus hudsoni* | Hudson's Black-Tyrant | Viudita Chica | 3 |
| 336 | *Knipolegus signatus* | Andean Tyrant | Viudita Plomiza | 3 |
| 337 | *Knipolegus cyanirostris* | Blue-billed Black-Tyrant | Viudita Pico Celeste | 3 |
| 338 | *Knipolegus aterrimus* | White-winged Black-Tyrant | Viudita Común | 7 |
| 339 | *Ochthoeca oenanthoides* | D'Orbigny's Chat-Tyrant | Pitajo Canela | 1 |
| 340 | *Colorhamphus parvirostris* | Patagonian Tyrant | Peutrén | 3 |
| 341 | *Satrapa icterophrys* | Yellow-browed Tyrant | Suirirí Amarillo | 5 |
| 342 | *Neoxolmis rufiventris* | Chocolate-vented Tyrant | Monjita Chocolate | 1 |
| 343 | *Xolmis pyrope* | Fire-eyed Diucon | Diucón | 2 |
| 344 | *Xolmis cinereus* | Gray Monjita | Monjita Gris | 3 |
| 345 | *Xolmis coronatus* | Black-crowned Monjita | Monjita Coronada | 2 |
| 346 | *Xolmis irupero* | White Monjita | Monjita Blanca | 5 |
| 347 | *Xolmis rubetra* | Rusty-backed Monjita | Monjita Castaña | 2 |
| 348 | *Agriornis micropterus* | Gray-bellied Shrike-Tyrant | Gaucho Común | 6 |
| 349 | *Agriornis murinus* | Lesser Shrike-Tyrant | Gaucho Chico | 3 |
| 350 | *Muscisaxicola maculirostris* | Spot-billed Ground-Tyrant | Dormilona Chica | 5 |
| 351 | *Muscisaxicola cinereus* | Cinereous Ground-Tyrant | Dormilona Cenicienta | 1 |
| 352 | *Muscisaxicola flavinucha* | Ochre-naped Ground-Tyrant | Dormilona Fraile | 3 |
| 353 | *Muscisaxicola rufivertex* | Rufous-naped Ground-Tyrant | Dormilona Gris | 2 |
| 354 | *Muscisaxicola maclovianus* | Dark-faced Ground-Tyrant | Dormilona Cara Negra | 2 |
| 355 | *Muscisaxicola albilora* | White-browed Ground-Tyrant | Dormilona Ceja Blanca | 2 |
| 356 | *Muscisaxicola capistratus* | Cinnamon-bellied Ground-Tyrant | Dormilona Canela | 2 |
| 357 | *Muscisaxicola frontalis* | Black-fronted Ground-Tyrant | Dormilona Frente Negra | 2 |
| 358 | *Machetornis rixosa* | Cattle Tyrant | Picabuey | 3 |
| 359 | *Myiozetetes similis* | Social Flycatcher | Benteveo Mediano | 1 |
| 360 | *Pitangus sulphuratus* | Great Kiskadee | Benteveo Común | 8 |
| 361 | *Myiodynastes maculatus* | Streaked Flycatcher | Benteveo Rayado | 5 |
| 362 | *Tyrannus melancholicus* | Tropical Kingbird | Suirirí Real | 5 |
| 363 | *Tyrannus savana* | Fork-tailed Flycatcher | Tijereta | 3 |
| 364 | *Casiornis rufus* | Rufous Casiornis | Burlisto Castaño | 3 |
| 365 | *Myiarchus tuberculifer* | Dusky-capped Flycatcher | Burlisto Corona Negra | 3 |
| 366 | *Myiarchus swainsoni* | Swainson's Flycatcher | Burlisto Pico Canela | 2 |
| 367 | *Myiarchus tyrannulus* | Brown-crested Flycatcher | Burlisto Cola Castaña | 5 |
| 368 | *Pachyramphus viridis* | Green-backed Becard | Anambé Verdoso | 2 |
| 369 | *Pachyramphus polychopterus* | White-winged Becard | Anambé Común | 2 |
| 370 | *Pachyramphus validus* | Crested Becard | Anambé Grande | 3 |
| 371 | *Tachycineta leucorrhoa* | White-rumped Swallow | Golodrina Ceja Blanca | 3 |
| 372 | *Tachycineta meyeni* | Chilean Swallow | Golondrina Patagónica | 4 |
| 373 | *Alopochelidon fucata* | Tawny-headed Swallow | Golondrina Cabeza Rojiza | 2 |
| 374 | *Hirundo rustica* | Barn Swallow | Golondrina Tijerita | 3 |
| 375 | *Anthus lutescens* | Yellowish Pipit | Cachirla Chica | 4 |
| 376 | *Anthus correndera* | Correndera Pipit | Cachirla Común | 3 |
| 377 | *Anthus hellmayri* | Hellmayr's Pipit | Cachirla Pálida | 3 |
| 378 | *Donacobius atricapilla* | Black-capped Donacobius | Angú | 3 |
| 379 | *Troglodytes aedon* | House Wren | Ratona Común | 19 |
| 380 | *Troglodytes solstitialis* | Mountain Wren | Ratona Ceja Blanca | 3 |
| 381 | *Cistothorus platensis* | Sedge Wren | Ratona Aperdizada | 3 |
| 382 | *Mimus saturninus* | Chalk-browed Mockingbird | Calandria Grande | 6 |
| 383 | *Mimus patagonicus* | Patagonian Mockingbird | Calandria Mora | 5 |
| 384 | *Mimus dorsalis* | Brown-backed Mockingbird | Calandria Castaña | 3 |
| 385 | *Mimus triurus* | White-banded Mockingbird | Calandria Real | 8 |
| 386 | *Catharus dryas* | Spotted Nightingale-Thrush | Zorzalito Overo | 1 |
| 387 | *Catharus ustulatus* | Swainson's Thrush | Zorzalito Boreal | 3 |
| 388 | *Turdus chiguanco* | Chiguanco Thrush | Zorzal Chiguanco | 3 |
| 389 | *Turdus nigriceps* | Andean Slaty-Thrush | Zorzal Plomizo | 1 |
| 390 | *Turdus rufiventris* | Rufous-bellied Thrush | Zorzal Colorado | 11 |
| 391 | *Turdus falcklandii* | Austral Thrush | Zorzal Patagónico | 4 |
| 392 | *Turdus leucomelas* | Pale-breasted Thrush | Zorzal Sabiá | 3 |
| 393 | *Turdus amaurochalinus* | Creamy-bellied Thrush | Zorzal Chalchalero | 10 |
| 394 | *Turdus albicollis* | White-necked Thrush | Zorzal Collar Blanco | 3 |
| 395 | *Polioptila dumicola* | Masked Gnatcatcher | Tacuarita Azul | 7 |
| 396 | *Cyanocorax cyanomelas* | Purplish Jay | Urraca Morada | 2 |
| 397 | *Cyanocorax chrysops* | Plush-crested Jay | Urraca Común | 5 |
| 398 | *Passer domesticus* | House Sparrow | Gorrión | 7 |
| 399 | *Vireo olivaceus* | Red-eyed Vireo | Chiví Común | 5 |
| 400 | *Cyclarhis gujanensis* | Rufous-browed Peppershrike | Juan Chiviro | 4 |
| 401 | *Euphonia chlorotica* | Purple-throated Euphonia | Tangará Común | 3 |
| 402 | *Euphonia cyanocephala* | Golden-rumped Euphonia | Tangará Cabeza Celeste | 2 |
| 403 | *Euphonia pectoralis* | Chestnut-bellied Euphonia | Tangará Alcalde | 2 |
| 404 | *Carduelis crassirostris* | Thick-billed Siskin | Cabecitanegra Picudo | 3 |
| 405 | *Carduelis magellanica* | Hooded Siskin | Cabecitanegra Común | 4 |
| 406 | *Carduelis barbata* | Black-chinned Siskin | Cabecitanegra Austral | 2 |
| 407 | *Carduelis atrata* | Black Siskin | Negrillo | 4 |
| 408 | *Parula pitiayumi* | Tropical Parula | Pitiayumí | 7 |
| 409 | *Geothlypis aequinoctialis* | Masked Yellowthroat | Arañero Cara Negra | 4 |
| 410 | *Myioborus brunniceps* | Brown-capped Redstart | Arañero Corona Rojiza | 3 |
| 411 | *Basileuterus bivittatus* | Two-banded Warbler | Arañero Coronado Grande | 2 |
| 412 | *Basileuterus signatus* | Pale-legged Warbler | Arañero Ceja Amarilla | 2 |
| 413 | *Basileuterus culicivorus* | Golden-crowned Warbler | Arañero Coronado Chico | 2 |
| 414 | *Basileuterus leucoblepharus* | White-rimmed Warbler | Arañero Silbón | 2 |
| 415 | *Chlorospingus ophthalmicus* | Common Bush-Tanager | Frutero Yungueño | 4 |
| 416 | *Pyrrhocoma ruficeps* | Chestnut-headed Tanager | Pioró | 2 |
| 417 | *Thlypopsis sordida* | Orange-headed Tanager | Tangará Gris | 3 |
| 418 | *Thlypopsis ruficeps* | Rust-and-yellow Tanager | Tangará Alisero | 3 |
| 419 | *Tachyphonus coronatus* | Ruby-crowned Tanager | Frutero Coronado | 2 |
| 420 | *Tachyphonus rufus* | White-lined Tanager | Frutero Negro | 5 |
| 421 | *Trichothraupis melanops* | Black-goggled Tanager | Frutero Corona Amarilla | 3 |
| 422 | *Habia rubica* | Red-crowned Ant-Tanager | Fueguero Morado | 2 |
| 423 | *Piranga flava* | Hepatic Tanager | Fueguero Común | 1 |
| 424 | *Thraupis sayaca* | Sayaca Tanager | Celestino Común | 5 |
| 425 | *Thraupis bonariensis* | Blue-and-yellow Tanager | Naranjero | 7 |
| 426 | *Pipraeidea melanonota* | Fawn-breasted Tanager | Saíra de Antifaz | 2 |
| 427 | *Coryphospingus cucullatus* | Red-crested Finch | Brasita de Fuego | 8 |
| 428 | *Phrygilus atriceps* | Black-hooded Sierra-Finch | Comesebo Cabeza Negra | 5 |
| 429 | *Phrygilus gayi* | Gray-hooded Sierra-Finch | Comesebo Andino | 3 |
| 430 | *Phrygilus patagonicus* | Patagonian Sierra-Finch | Comesebo Patagónico | 3 |
| 431 | *Phrygilus fruticeti* | Mourning Sierra-Finch | Yal Negro | 5 |
| 432 | *Phrygilus unicolor* | Plumbeous Sierra-Finch | Yal Plomizo | 1 |
| 433 | *Phrygilus carbonarius* | Carbonated Sierra-Finch | Yal Carbonero | 3 |
| 434 | *Phrygilus alaudinus* | Band-tailed Sierra-Finch | Yal Platero | 3 |
| 435 | *Phrygilus plebejus* | Ash-breasted Sierra-Finch | Yal Chico | 4 |
| 436 | *Melanodera melanodera* | Canary-winged Finch | Yal Austral | 2 |
| 437 | *Donacospiza albifrons* | Long-tailed Reed-Finch | Cachilo Canela | 2 |
| 438 | *Diuca diuca* | Common Diuca-Finch | Diuca Común | 4 |
| 439 | *Poospiza hypochondria* | Rufous-sided Warbling-Finch | Monterita Pecho Gris | 3 |
| 440 | *Poospiza ornata* | Cinnamon Warbling-Finch | Monterita Canela | 3 |
| 441 | *Poospiza erythrophrys* | Rusty-browed Warbling-Finch | Monterita Ceja Rojiza | 3 |
| 442 | *Poospiza nigrorufa* | Black-and-rufous Warbling-Finch | Sietevestidos | 3 |
| 443 | *Poospiza baeri* | Tucuman Mountain-Finch | Monterita Serrana | 3 |
| 444 | *Poospiza torquata* | Ringed Warbling-Finch | Monterita de Collar | 3 |
| 445 | *Poospiza melanoleuca* | Black-capped Warbling-Finch | Monterita Cabeza Negra | 4 |
| 446 | *Volatinia jacarina* | Blue-black Grassquit | Volantinero | 4 |
| 447 | *Sporophila collaris* | Rusty-collared Seedeater | Corbatita Dominó | 5 |
| 448 | *Sporophila caerulescens* | Double-collared Seedeater | Corbatita Común | 6 |
| 449 | *Sporophila leucoptera* | White-bellied Seedeater | Corbatita Blanco | 1 |
| 450 | *Sporophila hypoxantha* | Tawny-bellied Seedeater | Capuchino Canela | 4 |
| 451 | *Sporophila ruficollis* | Dark-throated Seedeater | Capuchino Garganta Café | 3 |
| 452 | *Sporophila palustris* | Marsh Seedeater | Capuchino Pecho Blanco | 2 |
| 453 | *Sporophila hypochroma* | Gray-and-chestnut Seedeater | Capuchino Castaño | 1 |
| 454 | *Sporophila cinnamomea* | Chestnut Seedeater | Capuchino Corona Gris | 2 |
| 455 | *Sporophila zelichi* | Narosky's Seedeater | Capuchino de Collar | 1 |
| 456 | *Oryzoborus angolensis* | Chestnut-bellied Seed-Finch | Curió | 3 |
| 457 | *Amaurospiza moesta* | Blackish-blue Seedeater | Reinamora Enana | 1 |
| 458 | *Catamenia analis* | Band-tailed Seedeater | Piquitodeoro Común | 3 |
| 459 | *Catamenia inornata* | Plain-colored Seedeater | Piquitodeoro Grande | 3 |
| 460 | *Haplospiza unicolor* | Uniform Finch | Afechero Plomizo | 1 |
| 461 | *Diglossa sittoides* | Rusty Flowerpiercer | Payador Canela | 3 |
| 462 | *Sicalis flaveola* | Saffron Finch | Jilguero Dorado | 6 |
| 463 | *Sicalis luteola* | Grassland Yellow-Finch | Misto | 7 |
| 464 | *Sicalis luteocephala* | Citron-headed Yellow-Finch | Jilguero Corona Gris | 1 |
| 465 | *Sicalis olivascens* | Greenish Yellow-Finch | Jilguero Oliváceo | 7 |
| 466 | *Emberizoides herbicola* | Wedge-tailed Grass-Finch | Coludo Grande | 2 |
| 467 | *Emberizoides ypiranganus* | Lesser Grass-Finch | Coludo Chico | 2 |
| 468 | *Embernagra platensis* | Great Pampa-Finch | Verdón | 5 |
| 469 | *Gubernatrix cristata* | Yellow Cardinal | Cardenal Amarillo | 1 |
| 470 | *Paroaria coronata* | Red-crested Cardinal | Cardenal Común | 5 |
| 471 | *Paroaria capitata* | Yellow-billed Cardinal | Cadenilla | 7 |
| 472 | *Atlapetes citrinellus* | Yellow-striped Brush-Finch | Cerquero Amarillo | 3 |
| 473 | *Buarremon torquatus* | Stripe-headed Brush-Finch | Cerquero Vientre Blanco | 1 |
| 474 | *Arremon flavirostris* | Saffron-billed Sparrow | Cerquero de Collar | 7 |
| 475 | *Ammodramus humeralis* | Grassland Sparrow | Cachilo Ceja Amarilla | 5 |
| 476 | *Zonotrichia capensis* | Rufous-collared Sparrow | Chingolo | 9 |
| 477 | *Saltator coerulescens* | Grayish Saltator | Pepitero Gris | 5 |
| 478 | *Saltator similis* | Green-winged Saltator | Pepitero Verdoso | 3 |
| 479 | *Saltator aurantiirostris* | Golden-billed Saltator | Pepitero de Collar | 7 |
| 480 | *Pheucticus aureoventris* | Black-backed Grosbeak | Rey del Bosque | 3 |
| 481 | *Cyanocompsa brissonii* | Ultramarine Grosbeak | Reinamora Grande | 8 |
| 482 | *Dolichonyx oryzivorus* | Bobolink | Charlatán | 2 |
| 483 | *Chrysomus ruficapillus* | Chestnut-capped Blackbird | Varillero Congo | 5 |
| 484 | *Agelasticus cyanopus* | Unicolored Blackbird | Varillero Negro | 7 |
| 485 | *Agelasticus thilius* | Yellow-winged Blackbird | Varillero Ala Amarilla | 3 |
| 486 | *Sturnella superciliaris* | White-browed Blackbird | Pecho Colorado | 3 |
| 487 | *Sturnella loyca* | Long-tailed Meadowlark | Loica Común | 5 |
| 488 | *Agelaioides badius* | Bay-winged Cowbird | Tordo Músico | 8 |
| 489 | *Molothrus rufoaxillaris* | Screaming Cowbird | Tordo Pico Corto | 3 |
| 490 | *Molothrus bonariensis* | Shiny Cowbird | Tordo Renegrido | 8 |
| 491 | *Icterus cayanensis* | Epaulet Oriole | Boyerito | 7 |
| 492 | *Cacicus haemorrhous* | Red-rumped Cacique | Boyero Cacique | 2 |
| 493 | *Cacicus chrysopterus* | Golden-winged Cacique | Boyero Ala Amarilla | 7 |
| 494 | *Cacicus solitarius* | Solitary Cacique | Boyero Negro | 2 |
| 495 | *Psarocolius decumanus* | Crested Oropendola | Yapú | 1 |
| 496 | *Pseudoleistes guirahuro* | Yellow-rumped Marshbird | Pecho Amarillo Grande | 1 |
| 497 | *Pseudoleistes virescens* | Brown-and-yellow Marshbird | Pecho Amarillo Común | 2 |
| 498 | *Amblyramphus holosericeus* | Scarlet-headed Blackbird | Federal | 3 |
| 499 | *Curaeus curaeus* | Austral Blackbird | Tordo Patagónico | 3 |
| 500 | *Gnorimopsar chopi* | Chopi Blackbird | Chopí | 1 |
