# Supplementary material for: Probing Evolutionary Patterns in Neotropical Birds through DNA Barcodes
Source: PLoS One. 2009 Feb 5;4(2):e4379. doi: 10.1371/journal.pone.0004379 (PMC2632745; doi:10.1371/journal.pone.0004379)
Supplement: Table S2 — List of species with natural breeding ranges extending from North America to Argentina. The number of individuals sampled per continent is provided, plus mean genetic distance when applicable. (0.11 MB DOC) [file pone.0004379.s002.doc]

Table S2.

| **Order** | **Family** | **Species** | **n**  **(N.A.)** | **n**  **(ARG)** | **Mean dist. (%)** |
| --- | --- | --- | --- | --- | --- |
| Podicipediformes | Podicipedidae | *Tachybaptus dominicus* | 1 |  | n/a |
| Podicipediformes | Podicipedidae | *Podilymbus podiceps* | 5 | 1 | 0.90 |
| Procellariiformes | Phalacrocoracidae | *Phalacrocorax brasilianus* | 4 | 3 | 0.25 |
| Procellariiformes | Anhingidae | *Anhinga anhinga* | 1 | 1 | 0.47 |
| Charadriiformes | Rynchopidae | *Rynchops niger* | 2 | 3 | 0.12 |
| Ciconiiformes | Ardeidae | *Ardea alba* | 3 | 3 | 0.37 |
| Ciconiiformes | Ardeidae | *Egretta caerulea* | 2 | 1 | 0 |
| Ciconiiformes | Ardeidae | *Egretta thula* | 1 | 3 | 0.40 |
| Ciconiiformes | Ardeidae | *Bubulcus ibis* | 4 | 3 | 0 |
| Ciconiiformes | Ardeidae | *Nycticorax nycticorax* | 2 | 1 | 0.49 |
| Ciconiiformes | Ardeidae | *Ixobrychus exilis* | 2 |  | n/a |
| Ciconiiformes | Ciconiidae | *Mycteria americana* | 3 | 1 | 0 |
| Ciconiiformes | Threskiornithidae | *Plegadis chihi* | 6 | 3 | 0.37 |
| Ciconiiformes | Threskiornithidae | *Platalea ajaja* | 4 | 2 | 0.16 |
| Anseriformes | Anatidae | *Dendrocygna bicolor* |  |  | n/a |
| Anseriformes | Anatidae | *Dendrocygna autumnalis* | 1 | 2 | 0.3 |
| Anseriformes | Anatidae | *Cairina moschata* | 2 |  | n/a |
| Anseriformes | Anatidae | *Anas cyanoptera* | 2 | 5 | 0.14 |
| Falconiformes | Cathartidae | *Coragyps atratus* | 5 | 2 | 0.33 |
| Falconiformes | Cathartidae | *Cathartes aura* | 3 |  | n/a |
| Falconiformes | Accipitridae | *Elanoides forficatus* | 1 |  | n/a |
| Falconiformes | Accipitridae | *Elanus leucurus* | 2 | 1 | 0 |
| Falconiformes | Accipitridae | *Rostrhamus sociabilis* | 4 | 2 | 0.40 |
| Falconiformes | Accipitridae | *Parabuteo unicinctus* | 2 | 1 | 0.29 |
| Falconiformes | Accipitridae | *Buteo brachyurus* | 1 |  | n/a |
| Falconiformes | Accipitridae | *Buteo albicaudatus* | 2 | 1 | 0.60 |
| Falconiformes | Falconidae | *Falco sparverius* | 8 | 3 | 0.76 |
| Falconiformes | Falconidae | *Falco femoralis* | 1 | 4 | 0.23 |
| Falconiformes | Falconidae | *Falco peregrinus* | 5 | 1 | 0.22 |
| Gruiformes | Aramidae | *Aramus guarauna* | 3 | 3 | 0.27 |
| Gruiformes | Rallidae | *Laterallus jamaicensis* | 1 |  | n/a |
| Gruiformes | Rallidae | *Porphyrio martinica* | 2 |  | n/a |
| Gruiformes | Rallidae | *Gallinula chloropus* | 9 | 3 | 0.37 |
| Charadriiformes | Haematopodidae | *Haematopus palliatus* | 2 |  | n/a |
| Columbiformes | Columbidae | *Patagioenas fasciata* | 2 |  | n/a |
| Cuculiformes | Cuculidae | *Crotophaga ani* | 2 | 3 | 0.19 |
| Strigiformes | Tytonidae | *Tyto alba* | 4 | 1 | 0.37 |
| Strigiformes | Strigidae | *Bubo virginianus* | 6 |  | n/a |
| Strigiformes | Strigidae | *Glaucidium brasilianum* | 5 | 3 | 1.53 |
| Strigiformes | Strigidae | *Athene cunicularia* | 2 | 6 | 4.73 |
| Strigiformes | Strigidae | *Asio flammeus* | 5 | 1 | 3.23 |
| Caprimulgiformes | Caprimulgidae | *Nyctidromus albicollis* | 11 | 1 | 5.11 |
| Coraciiformes | Alcedinidae | *Chloroceryle americana* | 4 | 3 | 2.82 |
| Passeriformes | Tyrannidae | *Sayornis nigricans* | 3 |  | n/a |
| Passeriformes | Tyrannidae | *Pyrocephalus rubinus* | 11 | 6 | 0.81 |
| Passeriformes | Tyrannidae | *Pitangus sulphuratus* | 12 | 8 | 0.22 |
| Passeriformes | Tyrannidae | *Tyrannus melancholicus* | 7 | 5 | 0.63 |
| Passeriformes | Tyrannidae | *Myiarchus tuberculifer* | 8 | 3 | 2.32 |
| Passeriformes | Tyrannidae | *Myiarchus tyrannulus* | 9 | 5 | 0.23 |
| Passeriformes | Troglodytidae | *Troglodytes aedon* | 22 | 19 | 4.12 |
| Passeriformes | Troglodytidae | *Cistothorus platensis* | 2 | 3 | 6.25 |
| Passeriformes | Vireonidae | *Vireo olivaceus* | 14 | 5 | 2.59 |
| Passeriformes | Hirundinidae | *Hirundo rustica* | 3 | 3 | 0.12 |
| Passeriformes | Thraupidae | *Piranga flava* | 2 | 1 | 2.16 |
| Passeriformes | Icteridae | *Molothrus bonariensis* | 10 | 8 | 0.08 |
