# Supplementary material for: Probing Evolutionary Patterns in Neotropical Birds through DNA Barcodes
Source: PLoS One. 2009 Feb 5;4(2):e4379. doi: 10.1371/journal.pone.0004379 (PMC2632745; doi:10.1371/journal.pone.0004379)
Supplement: Table S3 — List of all specimens included in the study and their associated BOLD process IDs, museum numbers, and GenBank accession numbers. (1.74 MB DOC) [file pone.0004379.s003.doc]

Table S3.

| **Species** | **BOLD Process ID** | **Museum ID** | **GenBank Accession** |
| --- | --- | --- | --- |
| *Accipiter bicolor* | KBAR649-06 | MACN-Or-ct708 | FJ027014 |
| *Accipiter bicolor* | KBAR817-06 | MACN-Or-ct1561 | FJ027015 |
| *Accipiter bicolor* | KAARG270-07 | MACN-Or-ct2359 | FJ027016 |
| *Accipiter erythronemius* | KBARG050-07 | MACN-Or-ct1807 | FJ027017 |
| *Accipiter erythronemius* | KBARG057-07 | MACN-Or-ct1723 | FJ027018 |
| *Accipiter erythronemius* | KAARG400-07 | MACN-Or-cp16 | FJ027019 |
| *Accipiter erythronemius* | KAARG399-07 | MACN-Or-cp15 | FJ027020 |
| *Agelaioides badius* | KBAR840-06 | MACN-Or-ct1593 | FJ027021 |
| *Agelaioides badius* | KBAR841-06 | MACN-Or-ct1594 | FJ027022 |
| *Agelaioides badius* | KAARG577-07 | MACN-Or-ct3194 | FJ027023 |
| *Agelaioides badius* | KAARG182-07 | MACN-Or-ct2149 | FJ027024 |
| *Agelaioides badius* | KAARG606-07 | MACN-Or-ct3235 | FJ027025 |
| *Agelaioides badius* | KAARG179-07 | MACN-Or-ct2146 | FJ027026 |
| *Agelaioides badius* | KAARG088-07 | MACN-Or-ct1909 | FJ027027 |
| *Agelaioides badius* | KAARG285-07 | MACN-Or-ct2381 | FJ027028 |
| *Agelasticus cyanopus* | KBAR480-06 | MACN-Or-ct1356 | FJ027029 |
| *Agelasticus cyanopus* | KBAR487-06 | MACN-Or-ct1375 | FJ027030 |
| *Agelasticus cyanopus* | KBAR541-06 | MACN-Or-ct1481 | FJ027031 |
| *Agelasticus cyanopus* | KAARG166-07 | MACN-Or-ct2133 | FJ027032 |
| *Agelasticus cyanopus* | KAARG586-07 | MACN-Or-ct3207 | FJ027033 |
| *Agelasticus cyanopus* | KAARG609-07 | MACN-Or-ct3240 | FJ027034 |
| *Agelasticus cyanopus* | KAARG149-07 | MACN-Or-ct2115 | FJ027035 |
| *Agelasticus thilius* | KAARG163-07 | MACN-Or-ct2130 | FJ027036 |
| *Agelasticus thilius* | KAARG164-07 | MACN-Or-ct2131 | FJ027037 |
| *Agelasticus thilius* | KAARG056-07 | MACN-Or-ct1349 | FJ027038 |
| *Agriornis micropterus* | KBAR099-06 | MACN-Or-ct593 | FJ027039 |
| *Agriornis micropterus* | KBAR100-06 | MACN-Or-ct666 | FJ027040 |
| *Agriornis micropterus* | KBAR098-06 | MACN-Or-ct1099 | FJ027041 |
| *Agriornis micropterus* | KBARG223-07 | MACN-Or-ct2735 | FJ027042 |
| *Agriornis micropterus* | KBARG349-07 | MACN-Or-ct2833 | FJ027043 |
| *Agriornis micropterus* | KAARG335-07 | MACN-Or-ct3015 | FJ027044 |
| *Agriornis murinus* | KBARG192-07 | MACN-Or-ct2731 | FJ027045 |
| *Agriornis murinus* | KBARG308-07 | MACN-Or-ct2839 | FJ027046 |
| *Agriornis murinus* | KBARG208-07 | MACN-Or-ct2733 | FJ027047 |
| *Agyrtria versicolor* | KBARG381-07 | MACN-Or-ct2948 | FJ027048 |
| *Agyrtria versicolor* | KBARG405-07 | MACN-Or-ct2957 | FJ027049 |
| *Alopochelidon fucata* | KBAR924-06 | MACN-Or-ct1699 | FJ027050 |
| *Alopochelidon fucata* | KBAR920-06 | MACN-Or-ct1695 | FJ027051 |
| *Amaurospiza moesta* | KBARG289-07 | MACN-Or-ct2900 | FJ027052 |
| *Amazona aestiva* | KBARG146-07 | MACN-Or-ct3060 | FJ027053 |
| *Amazona aestiva* | KAARG364-07 | MACN-Or-ct3075 | FJ027054 |
| *Amazona aestiva* | KAARG279-07 | MACN-Or-ct2370 | FJ027055 |
| *Amazona tucumana* | KBAR672-06 | MACN-Or-ct958 | FJ027056 |
| *Amazona tucumana* | KBAR673-06 | MACN-Or-ct959 | FJ027057 |
| *Amazona tucumana* | KBAR652-06 | MACN-Or-ct743 | FJ027058 |
| *Amazonetta brasiliensis* | KBARG009-07 | MACN-Or-ct1759 | FJ027059 |
| *Amazonetta brasiliensis* | KAARG222-07 | MACN-Or-ct2198 | FJ027060 |
| *Amazonetta brasiliensis* | KBARG041-07 | MACN-Or-ct1758 | FJ027061 |
| *Amazonetta brasiliensis* | KBARG074-07 | MACN-Or-ct1757 | FJ027062 |
| *Amazonetta brasiliensis* | KAARG232-07 | MACN-Or-ct2219 | FJ027063 |
| *Amazonetta brasiliensis* | KAARG235-07 | MACN-Or-ct2226 | FJ027064 |
| *Amazonetta brasiliensis* | KAARG244-07 | MACN-Or-ct2240 | FJ027065 |
| *Amblyramphus holosericeus* | KBARG032-07 | MACN-Or-ct1748 | FJ027066 |
| *Amblyramphus holosericeus* | KAARG171-07 | MACN-Or-ct2138 | FJ027067 |
| *Amblyramphus holosericeus* | KAARG168-07 | MACN-Or-ct2135 | FJ027068 |
| *Ammodramus humeralis* | KBAR535-06 | MACN-Or-ct1471 | FJ027069 |
| *Ammodramus humeralis* | KBAR548-06 | MACN-Or-ct1495 | FJ027070 |
| *Ammodramus humeralis* | KBAR549-06 | MACN-Or-ct1496 | FJ027071 |
| *Ammodramus humeralis* | KAARG616-07 | MACN-Or-ct3250 | FJ027072 |
| *Ammodramus humeralis* | KAARG010-07 | MACN-Or-ct150 | FJ027073 |
| *Anairetes flavirostris* | KBARG004-07 | MACN-Or-ct1330 | FJ027074 |
| *Anairetes flavirostris* | KBARG067-07 | MACN-Or-ct1331 | FJ027075 |
| *Anairetes flavirostris* | KBAR101-06 | MACN-Or-ct1053 | FJ027076 |
| *Anairetes parulus* | KBAR104-06 | MACN-Or-ct850 | FJ027077 |
| *Anairetes parulus* | KBAR303-06 | MACN-Or-ct550 | FJ027078 |
| *Anairetes parulus* | KBAR311-06 | MACN-Or-ct680 | FJ027079 |
| *Anairetes parulus* | KBARG286-07 | MACN-Or-ct2820 | FJ027080 |
| *Anas bahamensis* | KBAR622-06 | MACN-Or-ct273 | FJ027081 |
| *Anas cyanoptera* | KBAR698-06 | MACN-Or-ct1178 | FJ027082 |
| *Anas cyanoptera* | KBAR704-06 | MACN-Or-ct1194 | FJ027083 |
| *Anas cyanoptera* | KAARG025-07 | MACN-Or-ct242 | FJ027084 |
| *Anas cyanoptera* | KAARG027-07 | MACN-Or-ct250 | FJ027085 |
| *Anas cyanoptera* | KAARG196-07 | MACN-Or-ct2164 | FJ027086 |
| *Anas flavirostris* | KBAR724-06 | MACN-Or-ct1262 | FJ027087 |
| *Anas flavirostris* | KBAR703-06 | MACN-Or-ct1189 | FJ027088 |
| *Anas flavirostris* | KBAR713-06 | MACN-Or-ct1220 | FJ027089 |
| *Anas flavirostris* | KBAR719-06 | MACN-Or-ct1245 | FJ027090 |
| *Anas flavirostris* | KBAR715-06 | MACN-Or-ct1233 | FJ027091 |
| *Anas georgica* | KBAR693-06 | MACN-Or-ct1165 | FJ027092 |
| *Anas georgica* | KBAR712-06 | MACN-Or-ct1219 | FJ027093 |
| *Anas georgica* | KBAR720-06 | MACN-Or-ct1250 | FJ027094 |
| *Anas georgica* | KBAR726-06 | MACN-Or-ct1265 | FJ027095 |
| *Anas georgica* | KAARG029-07 | MACN-Or-ct271 | FJ027096 |
| *Anas georgica* | KAARG242-07 | MACN-Or-ct2237 | FJ027097 |
| *Anas platalea* | KBAR721-06 | MACN-Or-ct1255 | FJ027098 |
| *Anas platalea* | KAARG240-07 | MACN-Or-ct2235 | FJ027099 |
| *Anas platalea* | KAARG245-07 | MACN-Or-ct2241 | FJ027100 |
| *Anas puna* | KBAR694-06 | MACN-Or-ct1167 | FJ027101 |
| *Anas puna* | KBAR727-06 | MACN-Or-ct1267 | FJ027102 |
| *Anas sibilatrix* | KBAR639-06 | MACN-Or-ct577 | FJ027103 |
| *Anas sibilatrix* | KBAR700-06 | MACN-Or-ct1186 | FJ027104 |
| *Anas sibilatrix* | KBAR701-06 | MACN-Or-ct1187 | FJ027105 |
| *Anas sibilatrix* | KBAR722-06 | MACN-Or-ct1258 | FJ027106 |
| *Anas sibilatrix* | KAARG028-07 | MACN-Or-ct251 | FJ027107 |
| *Anas sibilatrix* | KAARG241-07 | MACN-Or-ct2236 | FJ027108 |
| *Anas specularioides* | KBAR697-06 | MACN-Or-ct1175 | FJ027109 |
| *Anas specularioides* | KBAR714-06 | MACN-Or-ct1228 | FJ027110 |
| *Anas specularioides* | KBAR716-06 | MACN-Or-ct1234 | FJ027111 |
| *Anas specularioides* | KBAR718-06 | MACN-Or-ct1244 | FJ027112 |
| *Anas specularioides* | KBAR708-06 | MACN-Or-ct1199 | FJ027113 |
| *Anas versicolor* | KBAR615-06 | MACN-Or-ct246 | FJ027114 |
| *Anas versicolor* | KBAR616-06 | MACN-Or-ct249 | FJ027115 |
| *Anas versicolor* | KAARG218-07 | MACN-Or-ct2192 | FJ027116 |
| *Anas versicolor* | KAARG287-07 | MACN-Or-ct2384 | FJ027117 |
| *Anas versicolor* | KAARG288-07 | MACN-Or-ct2385 | FJ027118 |
| *Anas versicolor* | KAARG226-07 | MACN-Or-ct2207 | FJ027119 |
| *Anas versicolor* | KAARG227-07 | MACN-Or-ct2208 | FJ027120 |
| *Anas versicolor* | KAARG289-07 | MACN-Or-ct2386 | FJ027121 |
| *Anhinga anhinga* | KAARG276-07 | MACN-Or-ct2367 | FJ027122 |
| *Anthus correndera* | KBAR431-06 | MACN-Or-ct581 | FJ027123 |
| *Anthus correndera* | KBAR432-06 | MACN-Or-ct588 | FJ027124 |
| *Anthus correndera* | KBAR439-06 | MACN-Or-ct631 | FJ027125 |
| *Anthus hellmayri* | KBAR176-06 | MACN-Or-ct834 | FJ027126 |
| *Anthus hellmayri* | KBAR670-06 | MACN-Or-ct953 | FJ027127 |
| *Anthus hellmayri* | KBAR686-06 | MACN-Or-ct1050 | FJ027128 |
| *Anthus lutescens* | KBAR912-06 | MACN-Or-ct1686 | FJ027129 |
| *Anthus lutescens* | KBAR537-06 | MACN-Or-ct1474 | FJ027130 |
| *Anthus lutescens* | KBAR536-06 | MACN-Or-ct1473 | FJ027131 |
| *Anthus lutescens* | KBAR824-06 | MACN-Or-ct1569 | FJ027132 |
| *Anumbius annumbi* | KBAR556-06 | MACN-Or-ct1508 | FJ027133 |
| *Anumbius annumbi* | KBAR561-06 | MACN-Or-ct1514 | FJ027134 |
| *Anumbius annumbi* | KBAR890-06 | MACN-Or-ct1660 | FJ027135 |
| *Anumbius annumbi* | KAARG183-07 | MACN-Or-ct2150 | FJ027136 |
| *Aphrastura spinicauda* | KBAR035-06 | MACN-Or-ct613 | FJ027137 |
| *Aphrastura spinicauda* | KBARG195-07 | MACN-Or-ct2592 | FJ027138 |
| *Aphrastura spinicauda* | KBARG125-07 | MACN-Or-ct2568 | FJ027139 |
| *Aphrastura spinicauda* | KAARG329-07 | MACN-Or-ct3008 | FJ027140 |
| *Aphrastura spinicauda* | KAARG330-07 | MACN-Or-ct3010 | FJ027141 |
| *Aphrastura spinicauda* | KAARG342-07 | MACN-Or-ct3024 | FJ027142 |
| *Aramides cajanea* | KBARG120-07 | MACN-Or-ct1857 | FJ027143 |
| *Aramides cajanea* | KBARG158-07 | MACN-Or-ct1828 | FJ027144 |
| *Aramides cajanea* | KAARG002-07 | MACN-Or-ct60 | FJ027145 |
| *Aramides ypecaha* | KBAR835-06 | MACN-Or-ct1588 | FJ027146 |
| *Aramides ypecaha* | KBAR845-06 | MACN-Or-ct1599 | FJ027147 |
| *Aramides ypecaha* | KAARG156-07 | MACN-Or-ct2122 | FJ027148 |
| *Aramus guarauna* | KBAR565-06 | MACN-Or-ct1518 | FJ027149 |
| *Aramus guarauna* | KBAR898-06 | MACN-Or-ct1669 | FJ027150 |
| *Aramus guarauna* | KAARG065-07 | MACN-Or-ct1778 | FJ027151 |
| *Aratinga acuticaudata* | KAARG443-07 | MACN-Or-cp59 | FJ027152 |
| *Aratinga acuticaudata* | KAARG444-07 | MACN-Or-cp60 | FJ027153 |
| *Aratinga aurea* | KAARG442-07 | MACN-Or-cp58 | FJ027154 |
| *Aratinga leucophthalma* | KBAR916-06 | MACN-Or-ct1691 | FJ027155 |
| *Aratinga leucophthalma* | KBAR914-06 | MACN-Or-ct1689 | FJ027156 |
| *Aratinga mitrata* | KBAR655-06 | MACN-Or-ct773 | FJ027157 |
| *Aratinga mitrata* | KBAR676-06 | MACN-Or-ct984 | FJ027158 |
| *Aratinga mitrata* | KBAR677-06 | MACN-Or-ct986 | FJ027159 |
| *Ardea alba* | KBAR882-06 | MACN-Or-ct1650 | FJ027160 |
| *Ardea alba* | KBAR928-06 | MACN-Or-ct1704 | FJ027161 |
| *Ardea alba* | KAARG282-07 | MACN-Or-ct2373 | FJ027162 |
| *Ardea cocoi* | KBARG008-07 | MACN-Or-ct1795 | FJ027163 |
| *Ardea cocoi* | KBAR839-06 | MACN-Or-ct1592 | FJ027164 |
| *Arremon flavirostris* | KBAR226-06 | MACN-Or-ct1143 | FJ027165 |
| *Arremon flavirostris* | KBARG375-07 | MACN-Or-ct2964 | FJ027166 |
| *Arremon flavirostris* | KBAR225-06 | MACN-Or-ct1140 | FJ027167 |
| *Arremon flavirostris* | KBAR227-06 | MACN-Or-ct887 | FJ027168 |
| *Arremon flavirostris* | KBAR925-06 | MACN-Or-ct1700 | FJ027169 |
| *Arremon flavirostris* | KBAR768-06 | MACN-Or-ct1388 | FJ027170 |
| *Arremon flavirostris* | KAARG294-07 | MACN-Or-ct2393 | FJ027171 |
| *Asio flammeus* | KAARG041-07 | MACN-Or-ct338 | FJ027172 |
| *Asthenes anthoides* | KAARG337-07 | MACN-Or-ct3019 | FJ027173 |
| *Asthenes anthoides* | KAARG339-07 | MACN-Or-ct3021 | FJ027174 |
| *Asthenes anthoides* | KAARG344-07 | MACN-Or-ct3026 | FJ027175 |
| *Asthenes baeri* | KBAR366-06 | MACN-Or-ct035 | FJ027176 |
| *Asthenes baeri* | KBAR374-06 | MACN-Or-ct047 | FJ027177 |
| *Asthenes baeri* | KBARG194-07 | MACN-Or-ct2769 | FJ027178 |
| *Asthenes baeri* | KBARG265-07 | MACN-Or-ct2793 | FJ027179 |
| *Asthenes baeri* | KBAR376-06 | MACN-Or-ct050 | FJ027180 |
| *Asthenes dorbignyi* | KBAR320-06 | MACN-Or-ct849 | FJ027181 |
| *Asthenes dorbignyi* | KBAR036-06 | MACN-Or-ct1009 | FJ027182 |
| *Asthenes dorbignyi* | KBAR037-06 | MACN-Or-ct1034 | FJ027183 |
| *Asthenes modesta* | KBAR728-06 | MACN-Or-ct1268 | FJ027184 |
| *Asthenes modesta* | KBAR040-06 | MACN-Or-ct874 | FJ027185 |
| *Asthenes modesta* | KBAR039-06 | MACN-Or-ct1105 | FJ027186 |
| *Asthenes modesta* | KBAR041-06 | MACN-Or-ct882 | FJ027187 |
| *Asthenes patagonica* | KAARG317-07 | MACN-Or-ct2991 | FJ027188 |
| *Asthenes patagonica* | KAARG325-07 | MACN-Or-ct3001 | FJ027189 |
| *Asthenes patagonica* | KAARG326-07 | MACN-Or-ct3003 | FJ027190 |
| *Asthenes pyrrholeuca* | KBAR043-06 | MACN-Or-ct575 | FJ027191 |
| *Asthenes pyrrholeuca* | KBAR044-06 | MACN-Or-ct670 | FJ027192 |
| *Asthenes pyrrholeuca* | KBAR042-06 | MACN-Or-ct545 | FJ027193 |
| *Asthenes pyrrholeuca* | KBAR602-06 | MACN-Or-ct027 | FJ027194 |
| *Asthenes pyrrholeuca* | KBARG205-07 | MACN-Or-ct2644 | FJ027195 |
| *Asthenes pyrrholeuca* | KBARG206-07 | MACN-Or-ct2676 | FJ027196 |
| *Asthenes pyrrholeuca* | KAARG315-07 | MACN-Or-ct2986 | FJ027197 |
| *Asthenes pyrrholeuca* | KAARG316-07 | MACN-Or-ct2989 | FJ027198 |
| *Asthenes pyrrholeuca* | KAARG323-07 | MACN-Or-ct2999 | FJ027199 |
| *Asthenes steinbachi* | KBAR750-06 | MACN-Or-ct1322 | FJ027200 |
| *Athene cunicularia* | KBAR763-06 | MACN-Or-ct1381 | FJ027201 |
| *Athene cunicularia* | KBARG366-07 | MACN-Or-ct2819 | FJ027202 |
| *Athene cunicularia* | KBAR690-06 | MACN-Or-ct1108 | FJ027203 |
| *Athene cunicularia* | KBAR756-06 | MACN-Or-ct1360 | FJ027204 |
| *Athene cunicularia* | KBAR761-06 | MACN-Or-ct1378 | FJ027205 |
| *Athene cunicularia* | KAARG311-07 | MACN-Or-ct2821 | FJ027206 |
| *Atlapetes citrinellus* | KBAR229-06 | MACN-Or-ct715 | FJ027207 |
| *Atlapetes citrinellus* | KBAR230-06 | MACN-Or-ct716 | FJ027208 |
| *Atlapetes citrinellus* | KBAR341-06 | MACN-Or-ct948 | FJ027209 |
| *Attagis gayi* | KBAR433-06 | MACN-Or-ct606 | FJ027210 |
| *Attagis gayi* | KBAR454-06 | MACN-Or-ct1185 | FJ027211 |
| *Attagis malouinus* | KBAR389-06 | MACN-Or-ct290 | FJ027212 |
| *Automolus leucophthalmus* | KBARG315-07 | MACN-Or-ct2855 | FJ027213 |
| *Automolus leucophthalmus* | KBARG316-07 | MACN-Or-ct2841 | FJ027214 |
| *Automolus leucophthalmus* | KBARG339-07 | MACN-Or-ct2858 | FJ027215 |
| *Automolus leucophthalmus* | KBARG363-07 | MACN-Or-ct2862 | FJ027216 |
| *Baryphthengus ruficapillus* | KBARG323-07 | MACN-Or-ct2856 | FJ027217 |
| *Baryphthengus ruficapillus* | KBARG372-07 | MACN-Or-ct2850 | FJ027218 |
| *Baryphthengus ruficapillus* | KBARG389-07 | MACN-Or-ct2950 | FJ027219 |
| *Basileuterus bivittatus* | KBAR209-06 | MACN-Or-ct720 | FJ027220 |
| *Basileuterus bivittatus* | KBAR336-06 | MACN-Or-ct912 | FJ027221 |
| *Basileuterus culicivorus* | KBARG305-07 | MACN-Or-ct2887 | FJ027222 |
| *Basileuterus culicivorus* | KBARG393-07 | MACN-Or-ct2951 | FJ027223 |
| *Basileuterus leucoblepharus* | KBAR893-06 | MACN-Or-ct1663 | FJ027224 |
| *Basileuterus leucoblepharus* | KBAR833-06 | MACN-Or-ct1585 | FJ027225 |
| *Basileuterus signatus* | KBAR211-06 | MACN-Or-ct790 | FJ027226 |
| *Basileuterus signatus* | KBAR347-06 | MACN-Or-ct994 | FJ027227 |
| *Botaurus pinnatus* | KAARG392-07 | MACN-Or-cp8 | FJ027228 |
| *Brotogeris chiriri* | KAARG356-07 | MACN-Or-ct3064 | FJ027229 |
| *Buarremon torquatus* | KBAR231-06 | MACN-Or-ct982 | FJ027230 |
| *Bubo magellanicus* | KBARG065-07 | MACN-Or-ct1339 | FJ027231 |
| *Bubulcus ibis* | KBAR808-06 | MACN-Or-ct1538 | FJ027232 |
| *Bubulcus ibis* | KBAR809-06 | MACN-Or-ct1539 | FJ027233 |
| *Bubulcus ibis* | KBAR837-06 | MACN-Or-ct1590 | FJ027234 |
| *Busarellus nigricollis* | KAARG130-07 | MACN-Or-ct2084 | FJ027235 |
| *Buteo albicaudatus* | KAARG272-07 | MACN-Or-ct2361 | FJ027236 |
| *Buteogallus meridionalis* | KAARG267-07 | MACN-Or-ct2352 | FJ027237 |
| *Buteogallus meridionalis* | KAARG117-07 | MACN-Or-ct2063 | FJ027238 |
| *Buteogallus meridionalis* | KAARG139-07 | MACN-Or-ct2095 | FJ027239 |
| *Buteogallus urubitinga* | KBAR913-06 | MACN-Or-ct1687 | FJ027240 |
| *Buteogallus urubitinga* | KBARG016-07 | MACN-Or-ct1777 | FJ027241 |
| *Buteogallus urubitinga* | KAARG271-07 | MACN-Or-ct2360 | FJ027242 |
| *Buteo leucorrhous* | KBAR679-06 | MACN-Or-ct992 | FJ027243 |
| *Buteo leucorrhous* | KBAR680-06 | MACN-Or-ct993 | FJ027244 |
| *Buteo magnirostris* | KBAR758-06 | MACN-Or-ct1367 | FJ027245 |
| *Buteo magnirostris* | KBAR778-06 | MACN-Or-ct1424 | FJ027246 |
| *Buteo magnirostris* | KBARG077-07 | MACN-Or-ct061 | FJ027247 |
| *Buteo magnirostris* | KBARG407-07 | MACN-Or-ct3045 | FJ027248 |
| *Buteo magnirostris* | KBARG010-07 | MACN-Or-ct265 | FJ027249 |
| *Buteo magnirostris* | KAARG114-07 | MACN-Or-ct1947 | FJ027250 |
| *Buteo polyosoma* | KBAR691-06 | MACN-Or-ct1113 | FJ027251 |
| *Butorides striata* | KBARG083-07 | MACN-Or-ct1805 | FJ027252 |
| *Butorides striata* | KBARG089-07 | MACN-Or-ct1793 | FJ027253 |
| *Butorides striata* | KBARG030-07 | MACN-Or-ct1783 | FJ027254 |
| *Cacicus chrysopterus* | KBAR297-06 | MACN-Or-ct987 | FJ027255 |
| *Cacicus chrysopterus* | KBAR296-06 | MACN-Or-ct784 | FJ027256 |
| *Cacicus chrysopterus* | KBAR345-06 | MACN-Or-ct989 | FJ027257 |
| *Cacicus chrysopterus* | KBAR773-06 | MACN-Or-ct1402 | FJ027258 |
| *Cacicus chrysopterus* | KBAR791-06 | MACN-Or-ct1456 | FJ027259 |
| *Cacicus chrysopterus* | KAARG541-07 | MACN-Or-ct3146 | FJ027260 |
| *Cacicus chrysopterus* | KAARG601-07 | MACN-Or-ct3229 | FJ027261 |
| *Cacicus haemorrhous* | KBARG346-07 | MACN-Or-ct2878 | FJ027262 |
| *Cacicus haemorrhous* | KBARG303-07 | MACN-Or-ct2918 | FJ027263 |
| *Cacicus solitarius* | KBAR497-06 | MACN-Or-ct1403 | FJ027264 |
| *Cacicus solitarius* | KBAR513-06 | MACN-Or-ct1429 | FJ027265 |
| *Calidris bairdii* | KBAR463-06 | MACN-Or-ct1277 | FJ027266 |
| *Calidris bairdii* | KBAR467-06 | MACN-Or-ct1295 | FJ027267 |
| *Calidris bairdii* | KBAR475-06 | MACN-Or-ct1318 | FJ027268 |
| *Calidris bairdii* | KAARG352-07 | MACN-Or-ct3039 | FJ027269 |
| *Calidris melanotos* | KBARG053-07 | MACN-Or-ct1734 | FJ027270 |
| *Calidris melanotos* | KBARG080-07 | MACN-Or-ct1745 | FJ027271 |
| *Callipepla californica* | KBAR429-06 | MACN-Or-ct555 | FJ027272 |
| *Callipepla californica* | KBARG203-07 | MACN-Or-ct2593 | FJ027273 |
| *Callonetta leucophrys* | KBARG011-07 | MACN-Or-ct1756 | FJ027274 |
| *Callonetta leucophrys* | KBARG043-07 | MACN-Or-ct1755 | FJ027275 |
| *Callonetta leucophrys* | KAARG221-07 | MACN-Or-ct2195 | FJ027276 |
| *Callonetta leucophrys* | KBARG076-07 | MACN-Or-ct1754 | FJ027277 |
| *Callonetta leucophrys* | KAARG224-07 | MACN-Or-ct2205 | FJ027278 |
| *Callonetta leucophrys* | KAARG228-07 | MACN-Or-ct2209 | FJ027279 |
| *Campephilus leucopogon* | KBAR028-06 | MACN-Or-ct788 | FJ027280 |
| *Campephilus leucopogon* | KBAR027-06 | MACN-Or-ct721 | FJ027281 |
| *Campephilus leucopogon* | KBAR029-06 | MACN-Or-ct974 | FJ027282 |
| *Campephilus leucopogon* | KBAR930-06 | MACN-Or-ct1708 | FJ027283 |
| *Campephilus leucopogon* | KBAR857-06 | MACN-Or-ct1618 | FJ027284 |
| *Campephilus leucopogon* | KAARG141-07 | MACN-Or-ct2102 | FJ027285 |
| *Campephilus magellanicus* | KBAR438-06 | MACN-Or-ct618 | FJ027286 |
| *Campephilus magellanicus* | KBAR449-06 | MACN-Or-ct696 | FJ027287 |
| *Campephilus magellanicus* | KBAR450-06 | MACN-Or-ct705 | FJ027288 |
| *Camptostoma obsoletum* | KBARG110-07 | MACN-Or-ct1816 | FJ027289 |
| *Camptostoma obsoletum* | KAARG614-07 | MACN-Or-ct3248 | FJ027290 |
| *Campylorhamphus trochilirostris* | KAARG570-07 | MACN-Or-ct3186 | FJ027291 |
| *Campylorhamphus trochilirostris* | KAARG136-07 | MACN-Or-ct2092 | FJ027292 |
| *Campylorhamphus trochilirostris* | KAARG624-07 | MACN-Or-ct3274 | FJ027293 |
| *Caprimulgus parvulus* | KAARG129-07 | MACN-Or-ct2082 | FJ027294 |
| *Caprimulgus parvulus* | KAARG143-07 | MACN-Or-ct2105 | FJ027295 |
| *Caracara plancus* | KBARG013-07 | MACN-Or-ct1753 | FJ027296 |
| *Caracara plancus* | KBARG036-07 | MACN-Or-ct1767 | FJ027297 |
| *Caracara plancus* | KBARG093-07 | MACN-Or-ct1784 | FJ027298 |
| *Carduelis atrata* | KBAR321-06 | MACN-Or-ct858 | FJ027299 |
| *Carduelis atrata* | KBAR324-06 | MACN-Or-ct870 | FJ027300 |
| *Carduelis atrata* | KBAR350-06 | MACN-Or-ct1086 | FJ027301 |
| *Carduelis atrata* | KBAR741-06 | MACN-Or-ct1298 | FJ027302 |
| *Carduelis barbata* | KBAR424-06 | MACN-Or-ct537 | FJ027303 |
| *Carduelis barbata* | KBAR447-06 | MACN-Or-ct691 | FJ027304 |
| *Carduelis crassirostris* | KBAR222-06 | MACN-Or-ct1071 | FJ027305 |
| *Carduelis crassirostris* | KBAR325-06 | MACN-Or-ct872 | FJ027306 |
| *Carduelis crassirostris* | KBAR328-06 | MACN-Or-ct879 | FJ027307 |
| *Carduelis magellanica* | KBAR473-06 | MACN-Or-ct1314 | FJ027308 |
| *Carduelis magellanica* | KBAR545-06 | MACN-Or-ct1491 | FJ027309 |
| *Carduelis magellanica* | KBARG078-07 | MACN-Or-ct1729 | FJ027310 |
| *Carduelis magellanica* | KAARG206-07 | MACN-Or-ct2177 | FJ027311 |
| *Casiornis rufus* | KBAR926-06 | MACN-Or-ct1701 | FJ027312 |
| *Casiornis rufus* | KBAR880-06 | MACN-Or-ct1648 | FJ027313 |
| *Casiornis rufus* | KBAR885-06 | MACN-Or-ct1653 | FJ027314 |
| *Catamenia analis* | KBAR233-06 | MACN-Or-ct1035 | FJ027315 |
| *Catamenia analis* | KBAR232-06 | MACN-Or-ct1030 | FJ027316 |
| *Catamenia analis* | KBAR234-06 | MACN-Or-ct825 | FJ027317 |
| *Catamenia inornata* | KBAR329-06 | MACN-Or-ct880 | FJ027318 |
| *Catamenia inornata* | KBAR349-06 | MACN-Or-ct1049 | FJ027319 |
| *Catamenia inornata* | KBAR319-06 | MACN-Or-ct832 | FJ027320 |
| *Cathartes burrovianus* | KBAR800-06 | MACN-Or-ct1494 | FJ027321 |
| *Cathartes burrovianus* | KBARG049-07 | MACN-Or-ct1742 | FJ027322 |
| *Catharus dryas* | KBAR189-06 | MACN-Or-ct909 | FJ027323 |
| *Catharus ustulatus* | KBAR192-06 | MACN-Or-ct1037 | FJ027324 |
| *Catharus ustulatus* | KBAR313-06 | MACN-Or-ct768 | FJ027325 |
| *Catharus ustulatus* | KBAR333-06 | MACN-Or-ct901 | FJ027326 |
| *Celeus lugubris* | KBARG084-07 | MACN-Or-ct1736 | FJ027327 |
| *Celeus lugubris* | KBARG129-07 | MACN-Or-ct1876 | FJ027328 |
| *Celeus lugubris* | KAARG286-07 | MACN-Or-ct2382 | FJ027329 |
| *Certhiaxis cinnamomeus* | KBAR543-06 | MACN-Or-ct1485 | FJ027330 |
| *Certhiaxis cinnamomeus* | KBAR830-06 | MACN-Or-ct1579 | FJ027331 |
| *Certhiaxis cinnamomeus* | KBAR540-06 | MACN-Or-ct1479 | FJ027332 |
| *Certhiaxis cinnamomeus* | KAARG189-07 | MACN-Or-ct2157 | FJ027333 |
| *Certhiaxis cinnamomeus* | KAARG201-07 | MACN-Or-ct2170 | FJ027334 |
| *Certhiaxis cinnamomeus* | KAARG204-07 | MACN-Or-ct2175 | FJ027335 |
| *Chaetura andrei* | KBARG399-07 | MACN-Or-ct3043 | FJ027336 |
| *Charadrius alticola* | KBAR322-06 | MACN-Or-ct860 | FJ027337 |
| *Charadrius alticola* | KBAR736-06 | MACN-Or-ct1288 | FJ027338 |
| *Charadrius alticola* | KBAR738-06 | MACN-Or-ct1294 | FJ027339 |
| *Charadrius alticola* | KBAR009-06 | MACN-Or-ct1101 | FJ027340 |
| *Charadrius alticola* | KBAR008-06 | MACN-Or-ct1088 | FJ027341 |
| *Charadrius collaris* | KBAR520-06 | MACN-Or-ct1445 | FJ027342 |
| *Charadrius collaris* | KBAR521-06 | MACN-Or-ct1446 | FJ027343 |
| *Charadrius collaris* | KBARG045-07 | MACN-Or-ct1751 | FJ027344 |
| *Charadrius falklandicus* | KAARG008-07 | MACN-Or-ct144 | FJ027345 |
| *Charadrius falklandicus* | KAARG036-07 | MACN-Or-ct306 | FJ027346 |
| *Charadrius modestus* | KAARG370-07 | MACN-Or-ct3345 | FJ027347 |
| *Charadrius modestus* | KAARG371-07 | MACN-Or-ct3346 | FJ027348 |
| *Chiroxiphia caudata* | KBARG115-07 | MACN-Or-ct1980 | FJ027349 |
| *Chiroxiphia caudata* | KBARG147-07 | MACN-Or-ct1989 | FJ027350 |
| *Chiroxiphia caudata* | KBARG383-07 | MACN-Or-ct2979 | FJ027351 |
| *Chloephaga picta* | KBAR635-06 | MACN-Or-ct564 | FJ027352 |
| *Chloephaga picta* | KBAR642-06 | MACN-Or-ct652 | FJ027353 |
| *Chloephaga poliocephala* | KBAR723-06 | MACN-Or-ct1261 | FJ027354 |
| *Chloephaga poliocephala* | KBAR725-06 | MACN-Or-ct1264 | FJ027355 |
| *Chloroceryle amazona* | KBAR870-06 | MACN-Or-ct1635 | FJ027356 |
| *Chloroceryle amazona* | KBAR879-06 | MACN-Or-ct1647 | FJ027357 |
| *Chloroceryle amazona* | KBAR883-06 | MACN-Or-ct1651 | FJ027358 |
| *Chloroceryle americana* | KBAR503-06 | MACN-Or-ct1410 | FJ027359 |
| *Chloroceryle americana* | KBARG295-07 | MACN-Or-ct2916 | FJ027360 |
| *Chloroceryle americana* | KBARG370-07 | MACN-Or-ct2882 | FJ027361 |
| *Chlorospingus ophthalmicus* | KBAR238-06 | MACN-Or-ct713 | FJ027362 |
| *Chlorospingus ophthalmicus* | KBAR240-06 | MACN-Or-ct775 | FJ027363 |
| *Chlorospingus ophthalmicus* | KBAR340-06 | MACN-Or-ct944 | FJ027364 |
| *Chlorospingus ophthalmicus* | KBAR671-06 | MACN-Or-ct956 | FJ027365 |
| *Chlorostilbon aureoventris* | KBAR788-06 | MACN-Or-ct1449 | FJ027366 |
| *Chlorostilbon aureoventris* | KBAR934-06 | MACN-Or-ct1714 | FJ027367 |
| *Chlorostilbon aureoventris* | KBARG420-07 | MACN-Or-ct2976 | FJ027368 |
| *Chlorostilbon aureoventris* | KAARG539-07 | MACN-Or-ct3144 | FJ027369 |
| *Chlorostilbon aureoventris* | KAARG561-07 | MACN-Or-ct3174 | FJ027370 |
| *Chlorostilbon aureoventris* | KAARG291-07 | MACN-Or-ct2388 | FJ027371 |
| *Chrysomus ruficapillus* | KBAR570-06 | MACN-Or-ct1525 | FJ027372 |
| *Chrysomus ruficapillus* | KBAR571-06 | MACN-Or-ct1527 | FJ027373 |
| *Chrysomus ruficapillus* | KBAR572-06 | MACN-Or-ct1528 | FJ027374 |
| *Chrysomus ruficapillus* | KAARG362-07 | MACN-Or-ct3072 | FJ027375 |
| *Chrysomus ruficapillus* | KAARG173-07 | MACN-Or-ct2140 | FJ027376 |
| *Ciconia maguari* | KAARG268-07 | MACN-Or-ct2356 | FJ027377 |
| *Cinclodes atacamensis* | KBAR470-06 | MACN-Or-ct1304 | FJ027378 |
| *Cinclodes atacamensis* | KBAR461-06 | MACN-Or-ct1271 | FJ027379 |
| *Cinclodes atacamensis* | KBAR465-06 | MACN-Or-ct1287 | FJ027380 |
| *Cinclodes fuscus* | KBAR046-06 | MACN-Or-ct836 | FJ027381 |
| *Cinclodes fuscus* | KBAR047-06 | MACN-Or-ct843 | FJ027382 |
| *Cinclodes fuscus* | KBAR045-06 | MACN-Or-ct1076 | FJ027383 |
| *Cinclodes fuscus* | KBARG211-07 | MACN-Or-ct2594 | FJ027384 |
| *Cinclodes fuscus* | KBARG236-07 | MACN-Or-ct2649 | FJ027385 |
| *Cinclodes fuscus* | KAARG214-07 | MACN-Or-ct2186 | FJ027386 |
| *Cinclodes fuscus* | KAARG338-07 | MACN-Or-ct3020 | FJ027387 |
| *Cinclodes fuscus* | KAARG190-07 | MACN-Or-ct2158 | FJ027388 |
| *Cinclodes patagonicus* | KBARG198-07 | MACN-Or-ct2675 | FJ027389 |
| *Cinclodes patagonicus* | KBARG237-07 | MACN-Or-ct2681 | FJ027390 |
| *Cinclodes patagonicus* | KAARG349-07 | MACN-Or-ct3035 | FJ027391 |
| *Cinclodes patagonicus* | KAARG350-07 | MACN-Or-ct3036 | FJ027392 |
| *Circus buffoni* | KAARG125-07 | MACN-Or-ct2075 | FJ027393 |
| *Circus cinereus* | KAARG042-07 | MACN-Or-ct339 | FJ027394 |
| *Cistothorus platensis* | KBAR177-06 | MACN-Or-ct753 | FJ027395 |
| *Cistothorus platensis* | KBAR342-06 | MACN-Or-ct955 | FJ027396 |
| *Cistothorus platensis* | KBAR305-06 | MACN-Or-ct585 | FJ027397 |
| *Cnemotriccus fuscatus* | KBARG343-07 | MACN-Or-ct2928 | FJ027398 |
| *Coccyzus cinereus* | KAARG404-07 | MACN-Or-cp20 | FJ027399 |
| *Coccyzus melacoryphus* | KBARG144-07 | MACN-Or-ct1861 | FJ027400 |
| *Coccyzus melacoryphus* | KAARG280-07 | MACN-Or-ct2371 | FJ027401 |
| *Coccyzus melacoryphus* | KBARG152-07 | MACN-Or-ct1863 | FJ027402 |
| *Colaptes campestris* | KBAR514-06 | MACN-Or-ct1431 | FJ027403 |
| *Colaptes campestris* | KBAR558-06 | MACN-Or-ct1511 | FJ027404 |
| *Colaptes melanochloros* | KBAR492-06 | MACN-Or-ct1393 | FJ027405 |
| *Colaptes melanochloros* | KBAR508-06 | MACN-Or-ct1415 | FJ027406 |
| *Colaptes melanochloros* | KBAR832-06 | MACN-Or-ct1584 | FJ027407 |
| *Colaptes melanochloros* | KAARG579-07 | MACN-Or-ct3197 | FJ027408 |
| *Colaptes melanochloros* | KAARG148-07 | MACN-Or-ct2114 | FJ027409 |
| *Colaptes melanochloros* | KAARG109-07 | MACN-Or-ct1936 | FJ027410 |
| *Colaptes pitius* | KBARG101-07 | MACN-Or-ct2565 | FJ027411 |
| *Colaptes pitius* | KBARG258-07 | MACN-Or-ct2616 | FJ027412 |
| *Colaptes pitius* | KAARG298-07 | MACN-Or-ct2582 | FJ027413 |
| *Colaptes rupicola* | KBAR030-06 | MACN-Or-ct847 | FJ027414 |
| *Colibri coruscans* | KBAR659-06 | MACN-Or-ct814 | FJ027415 |
| *Colibri coruscans* | KBAR662-06 | MACN-Or-ct821 | FJ027416 |
| *Colibri coruscans* | KBAR663-06 | MACN-Or-ct838 | FJ027417 |
| *Colorhamphus parvirostris* | KBARG218-07 | MACN-Or-ct2598 | FJ027418 |
| *Colorhamphus parvirostris* | KBARG244-07 | MACN-Or-ct2652 | FJ027419 |
| *Colorhamphus parvirostris* | KBARG250-07 | MACN-Or-ct2614 | FJ027420 |
| *Columba livia* | KBARG055-07 | MACN-Or-ct379 | FJ027421 |
| *Columbina picui* | KBAR018-06 | MACN-Or-ct810 | FJ027422 |
| *Columbina picui* | KBAR598-06 | MACN-Or-ct008 | FJ027423 |
| *Columbina picui* | KBAR769-06 | MACN-Or-ct1392 | FJ027424 |
| *Columbina picui* | KBAR789-06 | MACN-Or-ct1451 | FJ027425 |
| *Columbina picui* | KAARG078-07 | MACN-Or-ct1897 | FJ027426 |
| *Columbina picui* | KAARG627-07 | MACN-Or-ct3277 | FJ027427 |
| *Columbina picui* | KAARG057-07 | MACN-Or-ct1484 | FJ027428 |
| *Columbina talpacoti* | KBAR581-06 | MACN-Or-ct1541 | FJ027429 |
| *Columbina talpacoti* | KBAR582-06 | MACN-Or-ct1542 | FJ027430 |
| *Columbina talpacoti* | KBAR583-06 | MACN-Or-ct1543 | FJ027431 |
| *Columbina talpacoti* | KAARG361-07 | MACN-Or-ct3071 | FJ027432 |
| *Conopophaga lineata* | KBARG162-07 | MACN-Or-ct1950 | FJ027433 |
| *Contopus fumigatus* | KBAR105-06 | MACN-Or-ct884 | FJ027434 |
| *Coragyps atratus* | KBAR793-06 | MACN-Or-ct1463 | FJ027435 |
| *Coragyps atratus* | KAARG283-07 | MACN-Or-ct2374 | FJ027436 |
| *Coryphistera alaudina* | KBARG176-07 | MACN-Or-ct1884 | FJ027437 |
| *Coryphistera alaudina* | KBARG161-07 | MACN-Or-ct1882 | FJ027438 |
| *Coryphistera alaudina* | KAARG145-07 | MACN-Or-ct2110 | FJ027439 |
| *Coryphospingus cucullatus* | KBAR526-06 | MACN-Or-ct1457 | FJ027440 |
| *Coryphospingus cucullatus* | KBAR528-06 | MACN-Or-ct1464 | FJ027441 |
| *Coryphospingus cucullatus* | KBAR539-06 | MACN-Or-ct1478 | FJ027442 |
| *Coryphospingus cucullatus* | KBARG377-07 | MACN-Or-ct2933 | FJ027443 |
| *Coryphospingus cucullatus* | KBARG385-07 | MACN-Or-ct2949 | FJ027444 |
| *Coryphospingus cucullatus* | KAARG600-07 | MACN-Or-ct3228 | FJ027445 |
| *Coryphospingus cucullatus* | KAARG096-07 | MACN-Or-ct1919 | FJ027446 |
| *Coryphospingus cucullatus* | KAARG033-07 | MACN-Or-ct294 | FJ027447 |
| *Corythopis delalandi* | KBARG123-07 | MACN-Or-ct1981 | FJ027448 |
| *Corythopis delalandi* | KBARG380-07 | MACN-Or-ct2965 | FJ027449 |
| *Corythopis delalandi* | KBARG394-07 | MACN-Or-ct2937 | FJ027450 |
| *Coscoroba coscoroba* | KBAR638-06 | MACN-Or-ct567 | FJ027451 |
| *Coscoroba coscoroba* | KBAR643-06 | MACN-Or-ct653 | FJ027452 |
| *Cranioleuca pyrrhophia* | KBAR603-06 | MACN-Or-ct028 | FJ027453 |
| *Cranioleuca pyrrhophia* | KBAR608-06 | MACN-Or-ct048 | FJ027454 |
| *Cranioleuca pyrrhophia* | KBAR604-06 | MACN-Or-ct031 | FJ027455 |
| *Cranioleuca pyrrhophia* | KBAR049-06 | MACN-Or-ct796 | FJ027456 |
| *Cranioleuca pyrrhophia* | KBAR050-06 | MACN-Or-ct999 | FJ027457 |
| *Cranioleuca pyrrhophia* | KBARG193-07 | MACN-Or-ct2751 | FJ027458 |
| *Crotophaga ani* | KBAR505-06 | MACN-Or-ct1412 | FJ027459 |
| *Crotophaga ani* | KBAR506-06 | MACN-Or-ct1413 | FJ027460 |
| *Crotophaga ani* | KAARG064-07 | MACN-Or-ct1776 | FJ027461 |
| *Crypturellus tataupa* | KAARG461-07 | MACN-Or-cp77 | FJ027462 |
| *Curaeus curaeus* | KBARG190-07 | MACN-Or-ct2671 | FJ027463 |
| *Curaeus curaeus* | KBARG204-07 | MACN-Or-ct2625 | FJ027464 |
| *Curaeus curaeus* | KBARG252-07 | MACN-Or-ct2658 | FJ027465 |
| *Cyanocompsa brissonii* | KBAR242-06 | MACN-Or-ct1150 | FJ027466 |
| *Cyanocompsa brissonii* | KBAR241-06 | MACN-Or-ct1142 | FJ027467 |
| *Cyanocompsa brissonii* | KBAR334-06 | MACN-Or-ct902 | FJ027468 |
| *Cyanocompsa brissonii* | KBAR766-06 | MACN-Or-ct1386 | FJ027469 |
| *Cyanocompsa brissonii* | KBAR792-06 | MACN-Or-ct1461 | FJ027470 |
| *Cyanocompsa brissonii* | KBARG027-07 | MACN-Or-ct1384 | FJ027471 |
| *Cyanocompsa brissonii* | KBARG090-07 | MACN-Or-ct1706 | FJ027472 |
| *Cyanocompsa brissonii* | KAARG138-07 | MACN-Or-ct2094 | FJ027473 |
| *Cyanocorax chrysops* | KBAR203-06 | MACN-Or-ct1137 | FJ027474 |
| *Cyanocorax chrysops* | KBAR771-06 | MACN-Or-ct1395 | FJ027475 |
| *Cyanocorax chrysops* | KBAR781-06 | MACN-Or-ct1430 | FJ027476 |
| *Cyanocorax chrysops* | KBAR797-06 | MACN-Or-ct1486 | FJ027477 |
| *Cyanocorax chrysops* | KAARG590-07 | MACN-Or-ct3215 | FJ027478 |
| *Cyanocorax cyanomelas* | KBAR901-06 | MACN-Or-ct1674 | FJ027479 |
| *Cyanocorax cyanomelas* | KBARG094-07 | MACN-Or-ct1814 | FJ027480 |
| *Cyanoliseus patagonus* | KBAR631-06 | MACN-Or-ct551 | FJ027481 |
| *Cyanoliseus patagonus* | KBARG191-07 | MACN-Or-ct2700 | FJ027482 |
| *Cyanoliseus patagonus* | KBARG207-07 | MACN-Or-ct2707 | FJ027483 |
| *Cyclarhis gujanensis* | KBAR765-06 | MACN-Or-ct1383 | FJ027484 |
| *Cyclarhis gujanensis* | KBAR784-06 | MACN-Or-ct1441 | FJ027485 |
| *Cyclarhis gujanensis* | KBAR796-06 | MACN-Or-ct1482 | FJ027486 |
| *Cyclarhis gujanensis* | KBAR204-06 | MACN-Or-ct1118 | FJ027487 |
| *Cygnus melancoryphus* | KBAR636-06 | MACN-Or-ct565 | FJ027488 |
| *Cygnus melancoryphus* | KBAR637-06 | MACN-Or-ct566 | FJ027489 |
| *Dendrocincla turdina* | KBARG281-07 | MACN-Or-ct2897 | FJ027490 |
| *Dendrocincla turdina* | KBARG402-07 | MACN-Or-ct2939 | FJ027491 |
| *Dendrocincla turdina* | KBARG409-07 | MACN-Or-ct2958 | FJ027492 |
| *Dendrocolaptes platyrostris* | KAARG543-07 | MACN-Or-ct3149 | FJ027493 |
| *Dendrocolaptes platyrostris* | KAARG615-07 | MACN-Or-ct3249 | FJ027494 |
| *Dendrocygna autumnalis* | KBAR614-06 | MACN-Or-ct245 | FJ027495 |
| *Dendrocygna autumnalis* | KAARG225-07 | MACN-Or-ct2206 | FJ027496 |
| *Dendrocygna viduata* | KBAR618-06 | MACN-Or-ct258 | FJ027497 |
| *Dendrocygna viduata* | KBAR619-06 | MACN-Or-ct259 | FJ027498 |
| *Dendrocygna viduata* | KBAR620-06 | MACN-Or-ct261 | FJ027499 |
| *Dendrocygna viduata* | KBARG097-07 | MACN-Or-ct1870 | FJ027500 |
| *Dendrocygna viduata* | KAARG219-07 | MACN-Or-ct2193 | FJ027501 |
| *Dendrocygna viduata* | KBARG105-07 | MACN-Or-ct1871 | FJ027502 |
| *Dendrocygna viduata* | KAARG229-07 | MACN-Or-ct2210 | FJ027503 |
| *Diglossa sittoides* | KBAR246-06 | MACN-Or-ct808 | FJ027504 |
| *Diglossa sittoides* | KBAR244-06 | MACN-Or-ct1012 | FJ027505 |
| *Diglossa sittoides* | KBAR344-06 | MACN-Or-ct979 | FJ027506 |
| *Diuca diuca* | KBAR443-06 | MACN-Or-ct654 | FJ027507 |
| *Diuca diuca* | KBAR441-06 | MACN-Or-ct647 | FJ027508 |
| *Diuca diuca* | KBAR448-06 | MACN-Or-ct693 | FJ027509 |
| *Diuca diuca* | KBARG245-07 | MACN-Or-ct2682 | FJ027510 |
| *Dolichonyx oryzivorus* | KAARG359-07 | MACN-Or-ct3068 | FJ027511 |
| *Dolichonyx oryzivorus* | KAARG360-07 | MACN-Or-ct3070 | FJ027512 |
| *Donacobius atricapilla* | KAARG622-07 | MACN-Or-ct3270 | FJ027513 |
| *Donacobius atricapilla* | KAARG588-07 | MACN-Or-ct3213 | FJ027514 |
| *Donacobius atricapilla* | KAARG581-07 | MACN-Or-ct3199 | FJ027515 |
| *Donacospiza albifrons* | KAARG199-07 | MACN-Or-ct2168 | FJ027516 |
| *Donacospiza albifrons* | KAARG209-07 | MACN-Or-ct2180 | FJ027517 |
| *Drymophila rubricollis* | KBARG282-07 | MACN-Or-ct2883 | FJ027518 |
| *Dryocopus lineatus* | KBAR407-06 | MACN-Or-ct481 | FJ027519 |
| *Dysithamnus mentalis* | KBARG331-07 | MACN-Or-ct2857 | FJ027520 |
| *Dysithamnus mentalis* | KBARG353-07 | MACN-Or-ct2894 | FJ027521 |
| *Dysithamnus mentalis* | KBARG355-07 | MACN-Or-ct2861 | FJ027522 |
| *Egretta caerulea* | KBAR628-06 | MACN-Or-ct390 | FJ027523 |
| *Egretta thula* | KBAR822-06 | MACN-Or-ct1567 | FJ027524 |
| *Egretta thula* | KBAR854-06 | MACN-Or-ct1615 | FJ027525 |
| *Egretta thula* | KBAR877-06 | MACN-Or-ct1645 | FJ027526 |
| *Elaenia albiceps* | KBAR106-06 | MACN-Or-ct712 | FJ027527 |
| *Elaenia albiceps* | KBAR304-06 | MACN-Or-ct557 | FJ027528 |
| *Elaenia albiceps* | KBAR306-06 | MACN-Or-ct611 | FJ027529 |
| *Elaenia albiceps* | KBAR648-06 | MACN-Or-ct704 | FJ027530 |
| *Elaenia obscura* | KBAR110-06 | MACN-Or-ct789 | FJ027531 |
| *Elaenia parvirostris* | KBAR111-06 | MACN-Or-ct890 | FJ027532 |
| *Elaenia parvirostris* | KBAR112-06 | MACN-Or-ct893 | FJ027533 |
| *Elaenia parvirostris* | KBAR496-06 | MACN-Or-ct1400 | FJ027534 |
| *Elaenia parvirostris* | KAARG086-07 | MACN-Or-ct1907 | FJ027535 |
| *Elaenia spectabilis* | KAARG085-07 | MACN-Or-ct1906 | FJ027536 |
| *Elaenia spectabilis* | KBARG153-07 | MACN-Or-ct1880 | FJ027537 |
| *Elaenia spectabilis* | KBARG096-07 | MACN-Or-ct1854 | FJ027538 |
| *Elaenia spectabilis* | KAARG140-07 | MACN-Or-ct2099 | FJ027539 |
| *Elaenia strepera* | KBAR114-06 | MACN-Or-ct1153 | FJ027540 |
| *Elaenia strepera* | KBAR113-06 | MACN-Or-ct1141 | FJ027541 |
| *Elaenia strepera* | KBAR115-06 | MACN-Or-ct731 | FJ027542 |
| *Elanus leucurus* | KBARG184-07 | MACN-Or-ct1885 | FJ027543 |
| *Eleothreptus anomalus* | KBARG168-07 | MACN-Or-ct1866 | FJ027544 |
| *Eleothreptus anomalus* | KBARG175-07 | MACN-Or-ct1867 | FJ027545 |
| *Eleothreptus anomalus* | KBARG160-07 | MACN-Or-ct1865 | FJ027546 |
| *Emberizoides herbicola* | KBAR532-06 | MACN-Or-ct1468 | FJ027547 |
| *Emberizoides herbicola* | KBAR812-06 | MACN-Or-ct1555 | FJ027548 |
| *Emberizoides ypiranganus* | KAARG417-07 | MACN-Or-cp33 | FJ027549 |
| *Emberizoides ypiranganus* | KAARG416-07 | MACN-Or-cp32 | FJ027550 |
| *Embernagra platensis* | KBAR814-06 | MACN-Or-ct1557 | FJ027551 |
| *Embernagra platensis* | KBAR852-06 | MACN-Or-ct1609 | FJ027552 |
| *Embernagra platensis* | KBAR827-06 | MACN-Or-ct1574 | FJ027553 |
| *Embernagra platensis* | KAARG213-07 | MACN-Or-ct2185 | FJ027554 |
| *Embernagra platensis* | KAARG191-07 | MACN-Or-ct2159 | FJ027555 |
| *Eremobius phoenicurus* | KBAR053-06 | MACN-Or-ct664 | FJ027556 |
| *Eremobius phoenicurus* | KBAR052-06 | MACN-Or-ct596 | FJ027557 |
| *Eremobius phoenicurus* | KBAR051-06 | MACN-Or-ct547 | FJ027558 |
| *Eremobius phoenicurus* | KBARG270-07 | MACN-Or-ct2728 | FJ027559 |
| *Eremobius phoenicurus* | KAARG314-07 | MACN-Or-ct2984 | FJ027560 |
| *Eremobius phoenicurus* | KAARG318-07 | MACN-Or-ct2992 | FJ027561 |
| *Eriocnemis glaucopoides* | KBAR653-06 | MACN-Or-ct764 | FJ027562 |
| *Eriocnemis glaucopoides* | KBAR674-06 | MACN-Or-ct962 | FJ027563 |
| *Eriocnemis glaucopoides* | KBAR675-06 | MACN-Or-ct978 | FJ027564 |
| *Eudromia elegans* | KBAR430-06 | MACN-Or-ct571 | FJ027565 |
| *Eudromia elegans* | KBARG210-07 | MACN-Or-ct2771 | FJ027566 |
| *Eudromia elegans* | KBARG263-07 | MACN-Or-ct2745 | FJ027567 |
| *Euphonia chlorotica* | KBAR819-06 | MACN-Or-ct1564 | FJ027568 |
| *Euphonia chlorotica* | KBARG312-07 | MACN-Or-ct2903 | FJ027569 |
| *Euphonia chlorotica* | KAARG597-07 | MACN-Or-ct3225 | FJ027570 |
| *Euphonia cyanocephala* | KBAR248-06 | MACN-Or-ct913 | FJ027571 |
| *Euphonia cyanocephala* | KBAR247-06 | MACN-Or-ct1149 | FJ027572 |
| *Euphonia pectoralis* | KBARG291-07 | MACN-Or-ct2866 | FJ027573 |
| *Euphonia pectoralis* | KBARG298-07 | MACN-Or-ct2867 | FJ027574 |
| *Euscarthmus meloryphus* | KAARG083-07 | MACN-Or-ct1904 | FJ027575 |
| *Falco femoralis* | KBAR899-06 | MACN-Or-ct1670 | FJ027576 |
| *Falco femoralis* | KBARG098-07 | MACN-Or-ct1886 | FJ027577 |
| *Falco femoralis* | KAARG274-07 | MACN-Or-ct2363 | FJ027578 |
| *Falco femoralis* | KAARG275-07 | MACN-Or-ct2364 | FJ027579 |
| *Falco peregrinus* | KAARG011-07 | MACN-Or-ct156 | FJ027580 |
| *Falco sparverius* | KBAR685-06 | MACN-Or-ct1040 | FJ027581 |
| *Falco sparverius* | KBAR815-06 | MACN-Or-ct1559 | FJ027582 |
| *Falco sparverius* | KBARG246-07 | MACN-Or-ct2722 | FJ027583 |
| *Forpus xanthopterygius* | KBARG416-07 | MACN-Or-ct2974 | FJ027584 |
| *Forpus xanthopterygius* | KBARG387-07 | MACN-Or-ct2980 | FJ027585 |
| *Fulica ardesiaca* | KBAR451-06 | MACN-Or-ct1166 | FJ027586 |
| *Fulica ardesiaca* | KBAR456-06 | MACN-Or-ct1206 | FJ027587 |
| *Fulica armillata* | KBAR445-06 | MACN-Or-ct658 | FJ027588 |
| *Fulica armillata* | KBAR453-06 | MACN-Or-ct1181 | FJ027589 |
| *Fulica armillata* | KBAR459-06 | MACN-Or-ct1218 | FJ027590 |
| *Fulica armillata* | KBAR460-06 | MACN-Or-ct1249 | FJ027591 |
| *Fulica cornuta* | KBAR455-06 | MACN-Or-ct1195 | FJ027592 |
| *Fulica gigantea* | KBAR452-06 | MACN-Or-ct1179 | FJ027593 |
| *Fulica rufifrons* | KBAR458-06 | MACN-Or-ct1217 | FJ027594 |
| *Furnarius rufus* | KBAR515-06 | MACN-Or-ct1433 | FJ027595 |
| *Furnarius rufus* | KBAR054-06 | MACN-Or-ct1033 | FJ027596 |
| *Furnarius rufus* | KBAR373-06 | MACN-Or-ct046 | FJ027597 |
| *Furnarius rufus* | KBAR482-06 | MACN-Or-ct1359 | FJ027598 |
| *Furnarius rufus* | KBAR517-06 | MACN-Or-ct1437 | FJ027599 |
| *Furnarius rufus* | KBARG302-07 | MACN-Or-ct2803 | FJ027600 |
| *Furnarius rufus* | KAARG554-07 | MACN-Or-ct3167 | FJ027601 |
| *Furnarius rufus* | KAARG546-07 | MACN-Or-ct3153 | FJ027602 |
| *Furnarius rufus* | KAARG111-07 | MACN-Or-ct1939 | FJ027603 |
| *Furnarius rufus* | KAARG212-07 | MACN-Or-ct2183 | FJ027604 |
| *Gallinago paraguaiae* | KBAR533-06 | MACN-Or-ct1469 | FJ027605 |
| *Gallinago paraguaiae* | KBAR551-06 | MACN-Or-ct1498 | FJ027606 |
| *Gallinago paraguaiae* | KBAR552-06 | MACN-Or-ct1499 | FJ027607 |
| *Gallinula chloropus* | KBAR507-06 | MACN-Or-ct1414 | FJ027608 |
| *Gallinula chloropus* | KBARG091-07 | MACN-Or-ct1788 | FJ027609 |
| *Gallinula chloropus* | KAARG113-07 | MACN-Or-ct1946 | FJ027610 |
| *Gallinula melanops* | KBAR917-06 | MACN-Or-ct1692 | FJ027611 |
| *Gallinula melanops* | KBARG114-07 | MACN-Or-ct3055 | FJ027612 |
| *Gampsonyx swainsonii* | KBAR776-06 | MACN-Or-ct1421 | FJ027613 |
| *Geositta cunicularia* | KAARG333-07 | MACN-Or-ct3013 | FJ027614 |
| *Geositta cunicularia* | KAARG340-07 | MACN-Or-ct3022 | FJ027615 |
| *Geositta cunicularia* | KAARG346-07 | MACN-Or-ct3029 | FJ027616 |
| *Geositta punensis* | KBAR353-06 | MACN-Or-ct1170 | FJ027617 |
| *Geositta punensis* | KBAR055-06 | MACN-Or-ct1098 | FJ027618 |
| *Geositta punensis* | KBAR057-06 | MACN-Or-ct1107 | FJ027619 |
| *Geositta punensis* | KBAR729-06 | MACN-Or-ct1269 | FJ027620 |
| *Geositta punensis* | KBAR742-06 | MACN-Or-ct1300 | FJ027621 |
| *Geothlypis aequinoctialis* | KBAR486-06 | MACN-Or-ct1374 | FJ027622 |
| *Geothlypis aequinoctialis* | KBAR577-06 | MACN-Or-ct1533 | FJ027623 |
| *Geothlypis aequinoctialis* | KAARG594-07 | MACN-Or-ct3220 | FJ027624 |
| *Geothlypis aequinoctialis* | KAARG549-07 | MACN-Or-ct3159 | FJ027625 |
| *Geranoaetus melanoleucus* | KAARG103-07 | MACN-Or-ct1927 | FJ027626 |
| *Geranospiza caerulescens* | KAARG398-07 | MACN-Or-cp14 | FJ027627 |
| *Glaucidium brasilianum* | KBARG002-07 | MACN-Or-ct1335 | FJ027628 |
| *Glaucidium brasilianum* | KBAR627-06 | MACN-Or-ct382 | FJ027629 |
| *Glaucidium brasilianum* | KAARG576-07 | MACN-Or-ct3193 | FJ027630 |
| *Glaucidium nanum* | KBARG188-07 | MACN-Or-ct2620 | FJ027631 |
| *Glaucidium nanum* | KBARG199-07 | MACN-Or-ct2701 | FJ027632 |
| *Gnorimopsar chopi* | KAARG070-07 | MACN-Or-ct1887 | FJ027633 |
| *Grallaria albigula* | KBAR089-06 | MACN-Or-ct1156 | FJ027634 |
| *Grallaria albigula* | KBAR090-06 | MACN-Or-ct910 | FJ027635 |
| *Gubernatrix cristata* | KBAR607-06 | MACN-Or-ct040 | FJ027636 |
| *Guira guira* | KAARG178-07 | MACN-Or-ct2145 | FJ027637 |
| *Habia rubica* | KBARG329-07 | MACN-Or-ct2890 | FJ027638 |
| *Habia rubica* | KBARG374-07 | MACN-Or-ct2977 | FJ027639 |
| *Haematopus ater* | KAARG040-07 | MACN-Or-ct335 | FJ027640 |
| *Haematopus leucopodus* | KAARG006-07 | MACN-Or-ct136 | FJ027641 |
| *Haplospiza unicolor* | KBARG396-07 | MACN-Or-ct2969 | FJ027642 |
| *Hemitriccus diops* | KBARG186-07 | MACN-Or-ct2039 | FJ027643 |
| *Hemitriccus margaritaceiventer* | KBAR479-06 | MACN-Or-ct1355 | FJ027644 |
| *Hemitriccus margaritaceiventer* | KBAR499-06 | MACN-Or-ct1405 | FJ027645 |
| *Hemitriccus margaritaceiventer* | KBAR547-06 | MACN-Or-ct1493 | FJ027646 |
| *Hemitriccus margaritaceiventer* | KAARG544-07 | MACN-Or-ct3150 | FJ027647 |
| *Hemitriccus margaritaceiventer* | KAARG566-07 | MACN-Or-ct3181 | FJ027648 |
| *Heteronetta atricapilla* | KAARG237-07 | MACN-Or-ct2228 | FJ027649 |
| *Himantopus melanurus* | KBAR855-06 | MACN-Or-ct1616 | FJ027650 |
| *Himantopus melanurus* | KBAR831-06 | MACN-Or-ct1580 | FJ027651 |
| *Himantopus melanurus* | KBAR853-06 | MACN-Or-ct1612 | FJ027652 |
| *Hirundo rustica* | KBARG143-07 | MACN-Or-ct1844 | FJ027653 |
| *Hirundo rustica* | KBARG135-07 | MACN-Or-ct1843 | FJ027654 |
| *Hirundo rustica* | KBARG151-07 | MACN-Or-ct1845 | FJ027655 |
| *Hydropsalis torquata* | KBAR491-06 | MACN-Or-ct1391 | FJ027656 |
| *Hydropsalis torquata* | KBAR846-06 | MACN-Or-ct1600 | FJ027657 |
| *Hydropsalis torquata* | KBAR873-06 | MACN-Or-ct1638 | FJ027658 |
| *Hydropsalis torquata* | KAARG098-07 | MACN-Or-ct1922 | FJ027659 |
| *Hydropsalis torquata* | KAARG097-07 | MACN-Or-ct1921 | FJ027660 |
| *Hylocharis chrysura* | KBARG307-07 | MACN-Or-ct2854 | FJ027661 |
| *Hylocharis chrysura* | KBARG322-07 | MACN-Or-ct2870 | FJ027662 |
| *Hylocharis chrysura* | KBAR757-06 | MACN-Or-ct1361 | FJ027663 |
| *Hylocharis chrysura* | KAARG580-07 | MACN-Or-ct3198 | FJ027664 |
| *Hylocharis chrysura* | KBARG113-07 | MACN-Or-ct1874 | FJ027665 |
| *Hylocharis chrysura* | KAARG540-07 | MACN-Or-ct3145 | FJ027666 |
| *Hymenops perspicillatus* | KBAR117-06 | MACN-Or-ct625 | FJ027667 |
| *Hymenops perspicillatus* | KBAR118-06 | MACN-Or-ct649 | FJ027668 |
| *Hymenops perspicillatus* | KBAR309-06 | MACN-Or-ct673 | FJ027669 |
| *Hymenops perspicillatus* | KBAR801-06 | MACN-Or-ct1503 | FJ027670 |
| *Hymenops perspicillatus* | KAARG175-07 | MACN-Or-ct2142 | FJ027671 |
| *Hymenops perspicillatus* | KAARG058-07 | MACN-Or-ct1603 | FJ027672 |
| *Hymenops perspicillatus* | KAARG208-07 | MACN-Or-ct2179 | FJ027673 |
| *Icterus cayanensis* | KBAR563-06 | MACN-Or-ct1516 | FJ027674 |
| *Icterus cayanensis* | KBAR542-06 | MACN-Or-ct1483 | FJ027675 |
| *Icterus cayanensis* | KBAR544-06 | MACN-Or-ct1489 | FJ027676 |
| *Icterus cayanensis* | KAARG562-07 | MACN-Or-ct3175 | FJ027677 |
| *Icterus cayanensis* | KAARG558-07 | MACN-Or-ct3171 | FJ027678 |
| *Icterus cayanensis* | KAARG152-07 | MACN-Or-ct2118 | FJ027679 |
| *Icterus cayanensis* | KAARG154-07 | MACN-Or-ct2120 | FJ027680 |
| *Ictinia mississippiensis* | KAARG266-07 | MACN-Or-ct2351 | FJ027681 |
| *Ictinia mississippiensis* | KAARG265-07 | MACN-Or-ct2350 | FJ027682 |
| *Ictinia plumbea* | KBARG068-07 | MACN-Or-ct1766 | FJ027683 |
| *Ictinia plumbea* | KAARG127-07 | MACN-Or-ct2077 | FJ027684 |
| *Ictinia plumbea* | KAARG126-07 | MACN-Or-ct2076 | FJ027685 |
| *Ixobrychus involucris* | KAARG389-07 | MACN-Or-cp5 | FJ027686 |
| *Jacana jacana* | KBAR525-06 | MACN-Or-ct1455 | FJ027687 |
| *Jacana jacana* | KBAR902-06 | MACN-Or-ct1675 | FJ027688 |
| *Knipolegus aterrimus* | KBAR751-06 | MACN-Or-ct1323 | FJ027689 |
| *Knipolegus aterrimus* | KBARG254-07 | MACN-Or-ct2724 | FJ027690 |
| *Knipolegus aterrimus* | KBAR316-06 | MACN-Or-ct824 | FJ027691 |
| *Knipolegus aterrimus* | KAARG053-07 | MACN-Or-ct841 | FJ027692 |
| *Knipolegus aterrimus* | KBAR120-06 | MACN-Or-ct1011 | FJ027693 |
| *Knipolegus aterrimus* | KBAR121-06 | MACN-Or-ct799 | FJ027694 |
| *Knipolegus aterrimus* | KAARG052-07 | MACN-Or-ct826 | FJ027695 |
| *Knipolegus cyanirostris* | KBAR843-06 | MACN-Or-ct1597 | FJ027696 |
| *Knipolegus cyanirostris* | KBAR875-06 | MACN-Or-ct1641 | FJ027697 |
| *Knipolegus cyanirostris* | KBAR878-06 | MACN-Or-ct1646 | FJ027698 |
| *Knipolegus hudsoni* | KBARG240-07 | MACN-Or-ct2760 | FJ027699 |
| *Knipolegus hudsoni* | KBARG271-07 | MACN-Or-ct2746 | FJ027700 |
| *Knipolegus hudsoni* | KBAR359-06 | MACN-Or-ct012 | FJ027701 |
| *Knipolegus signatus* | KBAR122-06 | MACN-Or-ct710 | FJ027702 |
| *Knipolegus signatus* | KBAR123-06 | MACN-Or-ct732 | FJ027703 |
| *Knipolegus signatus* | KBAR124-06 | MACN-Or-ct737 | FJ027704 |
| *Knipolegus striaticeps* | KBAR829-06 | MACN-Or-ct1577 | FJ027705 |
| *Larus dominicanus* | KBARG031-07 | MACN-Or-ct395 | FJ027706 |
| *Larus dominicanus* | KBARG033-07 | MACN-Or-ct1340 | FJ027707 |
| *Larus scoresbii* | KAARG005-07 | MACN-Or-ct131 | FJ027708 |
| *Larus scoresbii* | KAARG004-07 | MACN-Or-ct130 | FJ027709 |
| *Lathrotriccus euleri* | KBAR126-06 | MACN-Or-ct1138 | FJ027710 |
| *Lathrotriccus euleri* | KBAR127-06 | MACN-Or-ct905 | FJ027711 |
| *Lathrotriccus euleri* | KBAR125-06 | MACN-Or-ct1126 | FJ027712 |
| *Lepidocolaptes angustirostris* | KBAR478-06 | MACN-Or-ct1351 | FJ027713 |
| *Lepidocolaptes angustirostris* | KBAR498-06 | MACN-Or-ct1404 | FJ027714 |
| *Lepidocolaptes angustirostris* | KAARG575-07 | MACN-Or-ct3191 | FJ027715 |
| *Lepidocolaptes angustirostris* | KAARG169-07 | MACN-Or-ct2136 | FJ027716 |
| *Lepidocolaptes angustirostris* | KAARG151-07 | MACN-Or-ct2117 | FJ027717 |
| *Lepidocolaptes angustirostris* | KAARG626-07 | MACN-Or-ct3276 | FJ027718 |
| *Lepidocolaptes angustirostris* | KBAR894-06 | MACN-Or-ct1665 | FJ027719 |
| *Lepidocolaptes falcinellus* | KAARG428-07 | MACN-Or-cp44 | FJ027720 |
| *Leptasthenura aegithaloides* | KBARG277-07 | MACN-Or-ct2693 | FJ027721 |
| *Leptasthenura aegithaloides* | KBAR059-06 | MACN-Or-ct818 | FJ027722 |
| *Leptasthenura aegithaloides* | KBAR058-06 | MACN-Or-ct1073 | FJ027723 |
| *Leptasthenura aegithaloides* | KBAR060-06 | MACN-Or-ct573 | FJ027724 |
| *Leptasthenura aegithaloides* | KBAR640-06 | MACN-Or-ct604 | FJ027725 |
| *Leptasthenura aegithaloides* | KAARG319-07 | MACN-Or-ct2993 | FJ027726 |
| *Leptasthenura aegithaloides* | KAARG321-07 | MACN-Or-ct2995 | FJ027727 |
| *Leptasthenura aegithaloides* | KAARG322-07 | MACN-Or-ct2998 | FJ027728 |
| *Leptasthenura aegithaloides* | KAARG332-07 | MACN-Or-ct3012 | FJ027729 |
| *Leptasthenura aegithaloides* | KAARG341-07 | MACN-Or-ct3023 | FJ027730 |
| *Leptasthenura fuliginiceps* | KBAR062-06 | MACN-Or-ct1047 | FJ027731 |
| *Leptasthenura fuliginiceps* | KBAR061-06 | MACN-Or-ct1024 | FJ027732 |
| *Leptasthenura fuliginiceps* | KBAR063-06 | MACN-Or-ct1052 | FJ027733 |
| *Leptasthenura platensis* | KAARG030-07 | MACN-Or-ct282 | FJ027734 |
| *Leptopogon amaurocephalus* | KBARG386-07 | MACN-Or-ct2935 | FJ027735 |
| *Leptopogon amaurocephalus* | KBARG414-07 | MACN-Or-ct2943 | FJ027736 |
| *Leptopogon amaurocephalus* | KBARG417-07 | MACN-Or-ct2963 | FJ027737 |
| *Leptopogon amaurocephalus* | KBARG139-07 | MACN-Or-ct1983 | FJ027738 |
| *Leptopogon amaurocephalus* | KAARG376-07 | MACN-Or-ct1979 | FJ027739 |
| *Leptopogon amaurocephalus* | KAARG377-07 | MACN-Or-ct2025 | FJ027740 |
| *Leptotila megalura* | KBAR019-06 | MACN-Or-ct914 | FJ027741 |
| *Leptotila rufaxilla* | KAARG537-07 | MACN-Or-ct2981 | FJ027742 |
| *Leptotila verreauxi* | KAARG545-07 | MACN-Or-ct3152 | FJ027743 |
| *Leptotila verreauxi* | KAARG623-07 | MACN-Or-ct3271 | FJ027744 |
| *Leptotila verreauxi* | KBARG413-07 | MACN-Or-ct2962 | FJ027745 |
| *Leptotila verreauxi* | KBAR488-06 | MACN-Or-ct1376 | FJ027746 |
| *Leptotila verreauxi* | KBAR509-06 | MACN-Or-ct1417 | FJ027747 |
| *Leptotila verreauxi* | KBAR524-06 | MACN-Or-ct1454 | FJ027748 |
| *Lessonia oreas* | KBAR128-06 | MACN-Or-ct1083 | FJ027749 |
| *Lessonia oreas* | KBAR129-06 | MACN-Or-ct1087 | FJ027750 |
| *Lessonia oreas* | KBAR323-06 | MACN-Or-ct862 | FJ027751 |
| *Lessonia oreas* | KBAR731-06 | MACN-Or-ct1278 | FJ027752 |
| *Lessonia oreas* | KBAR733-06 | MACN-Or-ct1281 | FJ027753 |
| *Lessonia oreas* | KBAR737-06 | MACN-Or-ct1290 | FJ027754 |
| *Lessonia rufa* | KBAR132-06 | MACN-Or-ct570 | FJ027755 |
| *Lessonia rufa* | KBARG187-07 | MACN-Or-ct2591 | FJ027756 |
| *Lessonia rufa* | KBAR131-06 | MACN-Or-ct560 | FJ027757 |
| *Lessonia rufa* | KBAR133-06 | MACN-Or-ct629 | FJ027758 |
| *Lessonia rufa* | KAARG301-07 | MACN-Or-ct2651 | FJ027759 |
| *Lessonia rufa* | KAARG303-07 | MACN-Or-ct2710 | FJ027760 |
| *Leucippus chionogaster* | KBAR210-06 | MACN-Or-ct723 | FJ027761 |
| *Leucippus chionogaster* | KBAR656-06 | MACN-Or-ct782 | FJ027762 |
| *Leucippus chionogaster* | KBAR667-06 | MACN-Or-ct911 | FJ027763 |
| *Leucippus chionogaster* | KBAR668-06 | MACN-Or-ct934 | FJ027764 |
| *Machetornis rixosa* | KAARG605-07 | MACN-Or-ct3234 | FJ027765 |
| *Machetornis rixosa* | KBAR921-06 | MACN-Or-ct1696 | FJ027766 |
| *Machetornis rixosa* | KBAR803-06 | MACN-Or-ct1506 | FJ027767 |
| *Macronectes giganteus* | KAARG249-07 | MACN-Or-ct2295 | FJ027768 |
| *Manacus manacus* | KBARG354-07 | MACN-Or-ct2880 | FJ027769 |
| *Manacus manacus* | KBARG371-07 | MACN-Or-ct2863 | FJ027770 |
| *Manacus manacus* | KBARG395-07 | MACN-Or-ct3042 | FJ027771 |
| *Manacus manacus* | KAARG353-07 | MACN-Or-ct3046 | FJ027772 |
| *Manacus manacus* | KAARG312-07 | MACN-Or-ct2876 | FJ027773 |
| *Manacus manacus* | KAARG313-07 | MACN-Or-ct2877 | FJ027774 |
| *Mecocerculus leucophrys* | KBAR138-06 | MACN-Or-ct735 | FJ027775 |
| *Mecocerculus leucophrys* | KBAR139-06 | MACN-Or-ct740 | FJ027776 |
| *Mecocerculus leucophrys* | KBAR338-06 | MACN-Or-ct921 | FJ027777 |
| *Megaceryle torquata* | KBAR886-06 | MACN-Or-ct1654 | FJ027778 |
| *Megaceryle torquata* | KBAR938-06 | MACN-Or-ct1718 | FJ027779 |
| *Megaceryle torquata* | KBAR502-06 | MACN-Or-ct1409 | FJ027780 |
| *Megaceryle torquata* | KAARG162-07 | MACN-Or-ct2129 | FJ027781 |
| *Megascops choliba* | KBARG130-07 | MACN-Or-ct3057 | FJ027782 |
| *Megascops choliba* | KBARG411-07 | MACN-Or-ct3047 | FJ027783 |
| *Megascops choliba* | KAARG611-07 | MACN-Or-ct3244 | FJ027784 |
| *Megascops choliba* | KBAR900-06 | MACN-Or-ct1672 | FJ027785 |
| *Megascops hoyi* | KBAR654-06 | MACN-Or-ct767 | FJ027786 |
| *Melanerpes cactorum* | KAARG552-07 | MACN-Or-ct3164 | FJ027787 |
| *Melanerpes cactorum* | KAARG108-07 | MACN-Or-ct1935 | FJ027788 |
| *Melanerpes cactorum* | KAARG596-07 | MACN-Or-ct3224 | FJ027789 |
| *Melanerpes cactorum* | KAARG107-07 | MACN-Or-ct1934 | FJ027790 |
| *Melanerpes candidus* | KBAR884-06 | MACN-Or-ct1652 | FJ027791 |
| *Melanerpes candidus* | KBAR935-06 | MACN-Or-ct1715 | FJ027792 |
| *Melanerpes candidus* | KBAR936-06 | MACN-Or-ct1716 | FJ027793 |
| *Melanerpes candidus* | KAARG172-07 | MACN-Or-ct2139 | FJ027794 |
| *Melanerpes candidus* | KAARG170-07 | MACN-Or-ct2137 | FJ027795 |
| *Melanodera melanodera* | KAARG021-07 | MACN-Or-ct209 | FJ027796 |
| *Melanodera melanodera* | KAARG020-07 | MACN-Or-ct208 | FJ027797 |
| *Metriopelia aymara* | KBAR021-06 | MACN-Or-ct1100 | FJ027798 |
| *Metriopelia aymara* | KBAR020-06 | MACN-Or-ct1063 | FJ027799 |
| *Metriopelia aymara* | KBAR022-06 | MACN-Or-ct859 | FJ027800 |
| *Metriopelia aymara* | KBAR730-06 | MACN-Or-ct1274 | FJ027801 |
| *Metriopelia aymara* | KBAR754-06 | MACN-Or-ct1328 | FJ027802 |
| *Metriopelia melanoptera* | KBAR024-06 | MACN-Or-ct855 | FJ027803 |
| *Metriopelia melanoptera* | KBAR023-06 | MACN-Or-ct1068 | FJ027804 |
| *Milvago chimachima* | KBAR850-06 | MACN-Or-ct1607 | FJ027805 |
| *Milvago chimachima* | KBAR821-06 | MACN-Or-ct1566 | FJ027806 |
| *Milvago chimachima* | KBAR865-06 | MACN-Or-ct1628 | FJ027807 |
| *Milvago chimachima* | KBAR864-06 | MACN-Or-ct1627 | FJ027808 |
| *Mimus dorsalis* | KBAR188-06 | MACN-Or-ct1082 | FJ027809 |
| *Mimus dorsalis* | KBAR187-06 | MACN-Or-ct1028 | FJ027810 |
| *Mimus dorsalis* | KBAR315-06 | MACN-Or-ct817 | FJ027811 |
| *Mimus patagonicus* | KBAR466-06 | MACN-Or-ct1289 | FJ027812 |
| *Mimus patagonicus* | KBARG215-07 | MACN-Or-ct2709 | FJ027813 |
| *Mimus patagonicus* | KBARG239-07 | MACN-Or-ct2739 | FJ027814 |
| *Mimus patagonicus* | KBARG262-07 | MACN-Or-ct2726 | FJ027815 |
| *Mimus patagonicus* | KBARG326-07 | MACN-Or-ct2807 | FJ027816 |
| *Mimus saturninus* | KBAR518-06 | MACN-Or-ct1439 | FJ027817 |
| *Mimus saturninus* | KBAR386-06 | MACN-Or-ct256 | FJ027818 |
| *Mimus saturninus* | KBAR387-06 | MACN-Or-ct268 | FJ027819 |
| *Mimus saturninus* | KBAR367-06 | MACN-Or-ct036 | FJ027820 |
| *Mimus saturninus* | KBAR510-06 | MACN-Or-ct1422 | FJ027821 |
| *Mimus saturninus* | KAARG181-07 | MACN-Or-ct2148 | FJ027822 |
| *Mimus triurus* | KBAR361-06 | MACN-Or-ct024 | FJ027823 |
| *Mimus triurus* | KBAR580-06 | MACN-Or-ct1540 | FJ027824 |
| *Mimus triurus* | KBAR851-06 | MACN-Or-ct1608 | FJ027825 |
| *Mimus triurus* | KBAR906-06 | MACN-Or-ct1680 | FJ027826 |
| *Mimus triurus* | KBARG285-07 | MACN-Or-ct2836 | FJ027827 |
| *Mimus triurus* | KBARG334-07 | MACN-Or-ct2808 | FJ027828 |
| *Mimus triurus* | KAARG197-07 | MACN-Or-ct2166 | FJ027829 |
| *Mimus triurus* | KAARG200-07 | MACN-Or-ct2169 | FJ027830 |
| *Mionectes rufiventris* | KBARG290-07 | MACN-Or-ct2884 | FJ027831 |
| *Mionectes rufiventris* | KBARG328-07 | MACN-Or-ct2905 | FJ027832 |
| *Mionectes rufiventris* | KBARG336-07 | MACN-Or-ct2906 | FJ027833 |
| *Mionectes rufiventris* | KBARG164-07 | MACN-Or-ct2045 | FJ027834 |
| *Molothrus bonariensis* | KBAR298-06 | MACN-Or-ct536 | FJ027835 |
| *Molothrus bonariensis* | KBAR575-06 | MACN-Or-ct1531 | FJ027836 |
| *Molothrus bonariensis* | KBAR798-06 | MACN-Or-ct1488 | FJ027837 |
| *Molothrus bonariensis* | KBAR802-06 | MACN-Or-ct1505 | FJ027838 |
| *Molothrus bonariensis* | KBARG217-07 | MACN-Or-ct2779 | FJ027839 |
| *Molothrus bonariensis* | KBARG318-07 | MACN-Or-ct2806 | FJ027840 |
| *Molothrus bonariensis* | KAARG619-07 | MACN-Or-ct3266 | FJ027841 |
| *Molothrus bonariensis* | KAARG354-07 | MACN-Or-ct3062 | FJ027842 |
| *Molothrus rufoaxillaris* | KAARG135-07 | MACN-Or-ct2091 | FJ027843 |
| *Molothrus rufoaxillaris* | KAARG185-07 | MACN-Or-ct2152 | FJ027844 |
| *Molothrus rufoaxillaris* | KAARG186-07 | MACN-Or-ct2153 | FJ027845 |
| *Muscisaxicola albilora* | KBAR140-06 | MACN-Or-ct852 | FJ027846 |
| *Muscisaxicola albilora* | KBAR735-06 | MACN-Or-ct1285 | FJ027847 |
| *Muscisaxicola capistratus* | KAARG045-07 | MACN-Or-ct362 | FJ027848 |
| *Muscisaxicola capistratus* | KAARG046-07 | MACN-Or-ct363 | FJ027849 |
| *Muscisaxicola cinereus* | KBAR464-06 | MACN-Or-ct1280 | FJ027850 |
| *Muscisaxicola flavinucha* | KBAR469-06 | MACN-Or-ct1301 | FJ027851 |
| *Muscisaxicola flavinucha* | KBAR462-06 | MACN-Or-ct1276 | FJ027852 |
| *Muscisaxicola flavinucha* | KBAR471-06 | MACN-Or-ct1305 | FJ027853 |
| *Muscisaxicola frontalis* | KBAR474-06 | MACN-Or-ct1316 | FJ027854 |
| *Muscisaxicola frontalis* | KBAR476-06 | MACN-Or-ct1324 | FJ027855 |
| *Muscisaxicola maclovianus* | KBARG220-07 | MACN-Or-ct2645 | FJ027856 |
| *Muscisaxicola maclovianus* | KBARG179-07 | MACN-Or-ct2562 | FJ027857 |
| *Muscisaxicola maculirostris* | KBAR141-06 | MACN-Or-ct591 | FJ027858 |
| *Muscisaxicola maculirostris* | KBAR142-06 | MACN-Or-ct592 | FJ027859 |
| *Muscisaxicola maculirostris* | KBAR732-06 | MACN-Or-ct1279 | FJ027860 |
| *Muscisaxicola maculirostris* | KBAR734-06 | MACN-Or-ct1283 | FJ027861 |
| *Muscisaxicola maculirostris* | KBAR753-06 | MACN-Or-ct1327 | FJ027862 |
| *Muscisaxicola rufivertex* | KBAR468-06 | MACN-Or-ct1299 | FJ027863 |
| *Muscisaxicola rufivertex* | KBAR472-06 | MACN-Or-ct1310 | FJ027864 |
| *Mycteria americana* | KBARG112-07 | MACN-Or-ct1856 | FJ027865 |
| *Myiarchus swainsoni* | KBAR358-06 | MACN-Or-ct009 | FJ027866 |
| *Myiarchus swainsoni* | KBARG365-07 | MACN-Or-ct2835 | FJ027867 |
| *Myiarchus tuberculifer* | KBAR143-06 | MACN-Or-ct742 | FJ027868 |
| *Myiarchus tuberculifer* | KBAR144-06 | MACN-Or-ct889 | FJ027869 |
| *Myiarchus tuberculifer* | KBAR145-06 | MACN-Or-ct951 | FJ027870 |
| *Myiarchus tyrannulus* | KBAR146-06 | MACN-Or-ct707 | FJ027871 |
| *Myiarchus tyrannulus* | KBAR519-06 | MACN-Or-ct1444 | FJ027872 |
| *Myiarchus tyrannulus* | KBAR887-06 | MACN-Or-ct1657 | FJ027873 |
| *Myiarchus tyrannulus* | KBAR892-06 | MACN-Or-ct1662 | FJ027874 |
| *Myiarchus tyrannulus* | KAARG625-07 | MACN-Or-ct3275 | FJ027875 |
| *Myioborus brunniceps* | KBAR214-06 | MACN-Or-ct750 | FJ027876 |
| *Myioborus brunniceps* | KBAR213-06 | MACN-Or-ct706 | FJ027877 |
| *Myioborus brunniceps* | KBAR339-06 | MACN-Or-ct943 | FJ027878 |
| *Myiodynastes maculatus* | KBAR147-06 | MACN-Or-ct1119 | FJ027879 |
| *Myiodynastes maculatus* | KBAR149-06 | MACN-Or-ct903 | FJ027880 |
| *Myiodynastes maculatus* | KBAR343-06 | MACN-Or-ct977 | FJ027881 |
| *Myiodynastes maculatus* | KBARG028-07 | MACN-Or-ct1787 | FJ027882 |
| *Myiodynastes maculatus* | KBARG052-07 | MACN-Or-ct1803 | FJ027883 |
| *Myiopagis viridicata* | KBARG292-07 | MACN-Or-ct2852 | FJ027884 |
| *Myiophobus fasciatus* | KBAR151-06 | MACN-Or-ct835 | FJ027885 |
| *Myiophobus fasciatus* | KBAR150-06 | MACN-Or-ct1114 | FJ027886 |
| *Myiophobus fasciatus* | KBAR762-06 | MACN-Or-ct1379 | FJ027887 |
| *Myiophobus fasciatus* | KAARG142-07 | MACN-Or-ct2104 | FJ027888 |
| *Myiopsitta monachus* | KBAR937-06 | MACN-Or-ct1717 | FJ027889 |
| *Myiopsitta monachus* | KBAR919-06 | MACN-Or-ct1694 | FJ027890 |
| *Myiopsitta monachus* | KAARG233-07 | MACN-Or-ct2220 | FJ027891 |
| *Myiopsitta monachus* | KAARG048-07 | MACN-Or-ct380 | FJ027892 |
| *Myiopsitta monachus* | KAARG234-07 | MACN-Or-ct2221 | FJ027893 |
| *Myiopsitta monachus* | KBARG167-07 | MACN-Or-ct1851 | FJ027894 |
| *Myiopsitta monachus* | KAARG230-07 | MACN-Or-ct2211 | FJ027895 |
| *Myiopsitta monachus* | KAARG231-07 | MACN-Or-ct2212 | FJ027896 |
| *Myiozetetes similis* | KBARG319-07 | MACN-Or-ct2921 | FJ027897 |
| *Neoxolmis rufiventris* | KAARG037-07 | MACN-Or-ct316 | FJ027898 |
| *Netta peposaca* | KBAR617-06 | MACN-Or-ct252 | FJ027899 |
| *Netta peposaca* | KAARG236-07 | MACN-Or-ct2227 | FJ027900 |
| *Netta peposaca* | KAARG223-07 | MACN-Or-ct2203 | FJ027901 |
| *Netta peposaca* | KAARG238-07 | MACN-Or-ct2230 | FJ027902 |
| *Netta peposaca* | KAARG239-07 | MACN-Or-ct2233 | FJ027903 |
| *Netta peposaca* | KAARG243-07 | MACN-Or-ct2238 | FJ027904 |
| *Nomonyx dominica* | KBARG106-07 | MACN-Or-ct3054 | FJ027905 |
| *Nonnula rubecula* | KBARG398-07 | MACN-Or-ct2938 | FJ027906 |
| *Nothoprocta cinerascens* | KAARG463-07 | MACN-Or-cp79 | FJ027907 |
| *Nothura darwinii* | KBAR001-06 | MACN-Or-ct1229 | FJ027908 |
| *Nothura maculosa* | KBAR557-06 | MACN-Or-ct1510 | FJ027909 |
| *Nothura maculosa* | KBAR842-06 | MACN-Or-ct1596 | FJ027910 |
| *Nothura maculosa* | KBAR849-06 | MACN-Or-ct1606 | FJ027911 |
| *Nothura maculosa* | KAARG187-07 | MACN-Or-ct2154 | FJ027912 |
| *Nyctibius griseus* | KBARG103-07 | MACN-Or-ct1837 | FJ027913 |
| *Nycticorax nycticorax* | KBAR939-06 | MACN-Or-ct1719 | FJ027914 |
| *Nyctidromus albicollis* | KBAR891-06 | MACN-Or-ct1661 | FJ027915 |
| *Nystalus chacuru* | KAARG393-07 | MACN-Or-cp9 | FJ027916 |
| *Ochetorhynchus certhioides* | KBAR354-06 | MACN-Or-ct003 | FJ027917 |
| *Ochetorhynchus certhioides* | KBAR370-06 | MACN-Or-ct042 | FJ027918 |
| *Ochetorhynchus certhioides* | KBARG257-07 | MACN-Or-ct2789 | FJ027919 |
| *Ochetorhynchus certhioides* | KAARG039-07 | MACN-Or-ct327 | FJ027920 |
| *Ochetorhynchus certhioides* | KAARG084-07 | MACN-Or-ct1905 | FJ027921 |
| *Ochthoeca oenanthoides* | KBAR152-06 | MACN-Or-ct1078 | FJ027922 |
| *Oreopholus ruficollis* | KAARG102-07 | MACN-Or-ct1926 | FJ027923 |
| *Oreotrochilus leucopleurus* | KBAR660-06 | MACN-Or-ct815 | FJ027924 |
| *Oreotrochilus leucopleurus* | KBAR681-06 | MACN-Or-ct1007 | FJ027925 |
| *Oreotrochilus leucopleurus* | KBAR689-06 | MACN-Or-ct1085 | FJ027926 |
| *Oreotrochilus leucopleurus* | KBARG242-07 | MACN-Or-ct2613 | FJ027927 |
| *Oryzoborus angolensis* | KBARG288-07 | MACN-Or-ct2915 | FJ027928 |
| *Oryzoborus angolensis* | KBARG313-07 | MACN-Or-ct2888 | FJ027929 |
| *Oryzoborus angolensis* | KBARG418-07 | MACN-Or-ct2944 | FJ027930 |
| *Oxyura ferruginea* | KBAR717-06 | MACN-Or-ct1238 | FJ027931 |
| *Pachyramphus polychopterus* | KAARG122-07 | MACN-Or-ct2071 | FJ027932 |
| *Pachyramphus polychopterus* | KAARG133-07 | MACN-Or-ct2087 | FJ027933 |
| *Pachyramphus validus* | KBAR154-06 | MACN-Or-ct1146 | FJ027934 |
| *Pachyramphus validus* | KBAR153-06 | MACN-Or-ct1131 | FJ027935 |
| *Pachyramphus validus* | KBAR155-06 | MACN-Or-ct717 | FJ027936 |
| *Pachyramphus viridis* | KAARG608-07 | MACN-Or-ct3239 | FJ027937 |
| *Pachyramphus viridis* | KAARG277-07 | MACN-Or-ct2368 | FJ027938 |
| *Parabuteo unicinctus* | KBAR612-06 | MACN-Or-ct147 | FJ027939 |
| *Pardirallus sanguinolentus* | KAARG194-07 | MACN-Or-ct2162 | FJ027940 |
| *Paroaria capitata* | KBAR774-06 | MACN-Or-ct1406 | FJ027941 |
| *Paroaria capitata* | KBAR787-06 | MACN-Or-ct1447 | FJ027942 |
| *Paroaria capitata* | KBAR790-06 | MACN-Or-ct1452 | FJ027943 |
| *Paroaria capitata* | KAARG167-07 | MACN-Or-ct2134 | FJ027944 |
| *Paroaria capitata* | KAARG146-07 | MACN-Or-ct2112 | FJ027945 |
| *Paroaria capitata* | KAARG592-07 | MACN-Or-ct3217 | FJ027946 |
| *Paroaria capitata* | KAARG591-07 | MACN-Or-ct3216 | FJ027947 |
| *Paroaria coronata* | KBAR755-06 | MACN-Or-ct1353 | FJ027948 |
| *Paroaria coronata* | KBAR775-06 | MACN-Or-ct1418 | FJ027949 |
| *Paroaria coronata* | KBAR799-06 | MACN-Or-ct1490 | FJ027950 |
| *Paroaria coronata* | KAARG572-07 | MACN-Or-ct3188 | FJ027951 |
| *Paroaria coronata* | KAARG573-07 | MACN-Or-ct3189 | FJ027952 |
| *Parula pitiayumi* | KBAR216-06 | MACN-Or-ct1001 | FJ027953 |
| *Parula pitiayumi* | KBAR218-06 | MACN-Or-ct904 | FJ027954 |
| *Parula pitiayumi* | KBAR348-06 | MACN-Or-ct997 | FJ027955 |
| *Parula pitiayumi* | KBAR904-06 | MACN-Or-ct1677 | FJ027956 |
| *Parula pitiayumi* | KBAR932-06 | MACN-Or-ct1711 | FJ027957 |
| *Parula pitiayumi* | KAARG560-07 | MACN-Or-ct3173 | FJ027958 |
| *Parula pitiayumi* | KAARG613-07 | MACN-Or-ct3247 | FJ027959 |
| *Passer domesticus* | KBARG035-07 | MACN-Or-ct1334 | FJ027960 |
| *Passer domesticus* | KAARG049-07 | MACN-Or-ct624 | FJ027961 |
| *Passer domesticus* | KAARG050-07 | MACN-Or-ct669 | FJ027962 |
| *Passer domesticus* | KAARG066-07 | MACN-Or-ct1826 | FJ027963 |
| *Passer domesticus* | KAARG067-07 | MACN-Or-ct1829 | FJ027964 |
| *Passer domesticus* | KAARG077-07 | MACN-Or-ct1896 | FJ027965 |
| *Passer domesticus* | KAARG001-07 | MACN-Or-ct53 | FJ027966 |
| *Patagioenas araucana* | KBAR434-06 | MACN-Or-ct607 | FJ027967 |
| *Patagioenas araucana* | KBARG260-07 | MACN-Or-ct2664 | FJ027968 |
| *Patagioenas araucana* | KBARG117-07 | MACN-Or-ct2567 | FJ027969 |
| *Patagioenas cayennensis* | KAARG118-07 | MACN-Or-ct2066 | FJ027970 |
| *Patagioenas maculosa* | KBAR918-06 | MACN-Or-ct1693 | FJ027971 |
| *Patagioenas maculosa* | KBARG248-07 | MACN-Or-ct2761 | FJ027972 |
| *Patagioenas maculosa* | KBARG256-07 | MACN-Or-ct2762 | FJ027973 |
| *Patagioenas picazuro* | KBAR574-06 | MACN-Or-ct1530 | FJ027974 |
| *Patagioenas picazuro* | KBAR567-06 | MACN-Or-ct1520 | FJ027975 |
| *Patagioenas picazuro* | KBAR584-06 | MACN-Or-ct1544 | FJ027976 |
| *Patagioenas picazuro* | KBARG069-07 | MACN-Or-ct399 | FJ027977 |
| *Patagioenas picazuro* | KAARG009-07 | MACN-Or-ct148 | FJ027978 |
| *Patagioenas picazuro* | KAARG115-07 | MACN-Or-ct1949 | FJ027979 |
| *Patagona gigas* | KBAR658-06 | MACN-Or-ct805 | FJ027980 |
| *Patagona gigas* | KBAR687-06 | MACN-Or-ct1055 | FJ027981 |
| *Patagona gigas* | KBAR688-06 | MACN-Or-ct1062 | FJ027982 |
| *Pelecanoides georgicus* | KAARG100-07 | MACN-Or-ct1924 | FJ027983 |
| *Pelecanoides georgicus* | KAARG101-07 | MACN-Or-ct1925 | FJ027984 |
| *Pelecanoides magellani* | KAARG032-07 | MACN-Or-ct289 | FJ027985 |
| *Phacellodomus maculipectus* | KBAR064-06 | MACN-Or-ct757 | FJ027986 |
| *Phacellodomus ruber* | KBAR594-06 | MACN-Or-ct1362 | FJ027987 |
| *Phacellodomus ruber* | KBAR595-06 | MACN-Or-ct1377 | FJ027988 |
| *Phacellodomus ruber* | KBAR596-06 | MACN-Or-ct1420 | FJ027989 |
| *Phacellodomus ruber* | KBAR764-06 | MACN-Or-ct1382 | FJ027990 |
| *Phacellodomus ruber* | KBAR859-06 | MACN-Or-ct1620 | FJ027991 |
| *Phacellodomus ruber* | KAARG548-07 | MACN-Or-ct3158 | FJ027992 |
| *Phacellodomus ruber* | KBARG095-07 | MACN-Or-ct1836 | FJ027993 |
| *Phacellodomus ruber* | KAARG604-07 | MACN-Or-ct3233 | FJ027994 |
| *Phacellodomus rufifrons* | KBAR065-06 | MACN-Or-ct794 | FJ027995 |
| *Phacellodomus striaticeps* | KBAR066-06 | MACN-Or-ct1015 | FJ027996 |
| *Phacellodomus striaticollis* | KAARG205-07 | MACN-Or-ct2176 | FJ027997 |
| *Phacellodomus striaticollis* | KAARG215-07 | MACN-Or-ct2187 | FJ027998 |
| *Phacellodomus striaticollis* | KAARG184-07 | MACN-Or-ct2151 | FJ027999 |
| *Phaeomyias murina* | KBAR169-06 | MACN-Or-ct998 | FJ028000 |
| *Phaeomyias murina* | KBAR156-06 | MACN-Or-ct795 | FJ028001 |
| *Phaethornis eurynome* | KBARG400-07 | MACN-Or-ct2970 | FJ028002 |
| *Phaethornis eurynome* | KBARG410-07 | MACN-Or-ct2941 | FJ028003 |
| *Phaetusa simplex* | KBAR858-06 | MACN-Or-ct1619 | FJ028004 |
| *Phalacrocorax atriceps* | KBARG023-07 | MACN-Or-ct1342 | FJ028005 |
| *Phalacrocorax atriceps* | KBARG086-07 | MACN-Or-ct1343 | FJ028006 |
| *Phalacrocorax brasilianus* | KBAR392-06 | MACN-Or-ct383 | FJ028007 |
| *Phalacrocorax brasilianus* | KBARG019-07 | MACN-Or-ct1739 | FJ028008 |
| *Phalacrocorax brasilianus* | KBARG071-07 | MACN-Or-ct1796 | FJ028009 |
| *Pheucticus aureoventris* | KBAR332-06 | MACN-Or-ct899 | FJ028010 |
| *Pheucticus aureoventris* | KBAR250-06 | MACN-Or-ct727 | FJ028011 |
| *Pheucticus aureoventris* | KBAR346-06 | MACN-Or-ct990 | FJ028012 |
| *Philydor atricapillus* | KBARG356-07 | MACN-Or-ct2847 | FJ028013 |
| *Philydor atricapillus* | KBARG397-07 | MACN-Or-ct2955 | FJ028014 |
| *Philydor lichtensteini* | KAARG378-07 | MACN-Or-ct2033 | FJ028015 |
| *Phimosus infuscatus* | KBAR523-06 | MACN-Or-ct1453 | FJ028016 |
| *Phimosus infuscatus* | KBAR550-06 | MACN-Or-ct1497 | FJ028017 |
| *Phimosus infuscatus* | KBAR861-06 | MACN-Or-ct1622 | FJ028018 |
| *Phleocryptes melanops* | KBAR067-06 | MACN-Or-ct572 | FJ028019 |
| *Phleocryptes melanops* | KBAR069-06 | MACN-Or-ct694 | FJ028020 |
| *Phleocryptes melanops* | KBAR068-06 | MACN-Or-ct677 | FJ028021 |
| *Phleocryptes melanops* | KAARG193-07 | MACN-Or-ct2161 | FJ028022 |
| *Phleocryptes melanops* | KAARG192-07 | MACN-Or-ct2160 | FJ028023 |
| *Phoenicopterus andinus* | KBAR707-06 | MACN-Or-ct1198 | FJ028024 |
| *Phoenicopterus andinus* | KBAR711-06 | MACN-Or-ct1216 | FJ028025 |
| *Phoenicopterus chilensis* | KBAR666-06 | MACN-Or-ct867 | FJ028026 |
| *Phoenicopterus chilensis* | KBAR705-06 | MACN-Or-ct1196 | FJ028027 |
| *Phoenicopterus chilensis* | KBAR706-06 | MACN-Or-ct1197 | FJ028028 |
| *Phoenicopterus jamesi* | KBAR702-06 | MACN-Or-ct1188 | FJ028029 |
| *Phoenicopterus jamesi* | KBAR710-06 | MACN-Or-ct1215 | FJ028030 |
| *Phrygilus alaudinus* | KBAR253-06 | MACN-Or-ct1029 | FJ028031 |
| *Phrygilus alaudinus* | KBAR252-06 | MACN-Or-ct1017 | FJ028032 |
| *Phrygilus alaudinus* | KBAR254-06 | MACN-Or-ct823 | FJ028033 |
| *Phrygilus atriceps* | KBAR256-06 | MACN-Or-ct1074 | FJ028034 |
| *Phrygilus atriceps* | KBAR255-06 | MACN-Or-ct1064 | FJ028035 |
| *Phrygilus atriceps* | KBAR327-06 | MACN-Or-ct878 | FJ028036 |
| *Phrygilus atriceps* | KBAR352-06 | MACN-Or-ct1169 | FJ028037 |
| *Phrygilus atriceps* | KBAR696-06 | MACN-Or-ct1173 | FJ028038 |
| *Phrygilus carbonarius* | KBARG231-07 | MACN-Or-ct2738 | FJ028039 |
| *Phrygilus carbonarius* | KBARG247-07 | MACN-Or-ct2740 | FJ028040 |
| *Phrygilus carbonarius* | KBARG200-07 | MACN-Or-ct2732 | FJ028041 |
| *Phrygilus fruticeti* | KBAR258-06 | MACN-Or-ct1031 | FJ028042 |
| *Phrygilus fruticeti* | KBAR326-06 | MACN-Or-ct876 | FJ028043 |
| *Phrygilus fruticeti* | KBAR307-06 | MACN-Or-ct661 | FJ028044 |
| *Phrygilus fruticeti* | KBAR644-06 | MACN-Or-ct662 | FJ028045 |
| *Phrygilus fruticeti* | KBAR747-06 | MACN-Or-ct1317 | FJ028046 |
| *Phrygilus gayi* | KBAR261-06 | MACN-Or-ct1018 | FJ028047 |
| *Phrygilus gayi* | KBAR262-06 | MACN-Or-ct1022 | FJ028048 |
| *Phrygilus gayi* | KBAR752-06 | MACN-Or-ct1325 | FJ028049 |
| *Phrygilus patagonicus* | KBAR436-06 | MACN-Or-ct615 | FJ028050 |
| *Phrygilus patagonicus* | KBAR437-06 | MACN-Or-ct617 | FJ028051 |
| *Phrygilus patagonicus* | KBARG165-07 | MACN-Or-ct2585 | FJ028052 |
| *Phrygilus plebejus* | KBAR264-06 | MACN-Or-ct1069 | FJ028053 |
| *Phrygilus plebejus* | KBAR266-06 | MACN-Or-ct869 | FJ028054 |
| *Phrygilus plebejus* | KBAR265-06 | MACN-Or-ct1089 | FJ028055 |
| *Phrygilus plebejus* | KBAR746-06 | MACN-Or-ct1315 | FJ028056 |
| *Phrygilus unicolor* | KBAR743-06 | MACN-Or-ct1302 | FJ028057 |
| *Phyllomyias sclateri* | KBAR157-06 | MACN-Or-ct1148 | FJ028058 |
| *Phyllomyias sclateri* | KBAR159-06 | MACN-Or-ct896 | FJ028059 |
| *Phyllomyias sclateri* | KBAR158-06 | MACN-Or-ct888 | FJ028060 |
| *Phylloscartes ventralis* | KBARG037-07 | MACN-Or-ct769 | FJ028061 |
| *Phylloscartes ventralis* | KBAR161-06 | MACN-Or-ct981 | FJ028062 |
| *Phytotoma rara* | KBARG197-07 | MACN-Or-ct2643 | FJ028063 |
| *Phytotoma rara* | KBARG243-07 | MACN-Or-ct2631 | FJ028064 |
| *Phytotoma rara* | KBARG214-07 | MACN-Or-ct2677 | FJ028065 |
| *Phytotoma rutila* | KBAR357-06 | MACN-Or-ct007 | FJ028066 |
| *Phytotoma rutila* | KBARG225-07 | MACN-Or-ct2780 | FJ028067 |
| *Phytotoma rutila* | KBARG300-07 | MACN-Or-ct2838 | FJ028068 |
| *Phytotoma rutila* | KBAR360-06 | MACN-Or-ct015 | FJ028069 |
| *Phytotoma rutila* | KBAR379-06 | MACN-Or-ct058 | FJ028070 |
| *Phytotoma rutila* | KAARG217-07 | MACN-Or-ct2189 | FJ028071 |
| *Phytotoma rutila* | KAARG180-07 | MACN-Or-ct2147 | FJ028072 |
| *Piaya cayana* | KBAR484-06 | MACN-Or-ct1365 | FJ028073 |
| *Piaya cayana* | KBAR485-06 | MACN-Or-ct1366 | FJ028074 |
| *Piaya cayana* | KBAR834-06 | MACN-Or-ct1586 | FJ028075 |
| *Picoides lignarius* | KBARG196-07 | MACN-Or-ct2623 | FJ028076 |
| *Picoides lignarius* | KBARG234-07 | MACN-Or-ct2611 | FJ028077 |
| *Picoides mixtus* | KBAR915-06 | MACN-Or-ct1690 | FJ028078 |
| *Picoides mixtus* | KAARG150-07 | MACN-Or-ct2116 | FJ028079 |
| *Picoides mixtus* | KAARG585-07 | MACN-Or-ct3206 | FJ028080 |
| *Picoides mixtus* | KAARG621-07 | MACN-Or-ct3268 | FJ028081 |
| *Picoides mixtus* | KAARG159-07 | MACN-Or-ct2126 | FJ028082 |
| *Picoides mixtus* | KAARG284-07 | MACN-Or-ct2376 | FJ028083 |
| *Piculus chrysochloros* | KBAR512-06 | MACN-Or-ct1427 | FJ028084 |
| *Piculus chrysochloros* | KBARG142-07 | MACN-Or-ct1825 | FJ028085 |
| *Piculus chrysochloros* | KAARG281-07 | MACN-Or-ct2372 | FJ028086 |
| *Picumnus cirratus* | KBAR033-06 | MACN-Or-ct886 | FJ028087 |
| *Picumnus cirratus* | KAARG582-07 | MACN-Or-ct3200 | FJ028088 |
| *Picumnus cirratus* | KBAR031-06 | MACN-Or-ct719 | FJ028089 |
| *Picumnus cirratus* | KBAR032-06 | MACN-Or-ct883 | FJ028090 |
| *Picumnus cirratus* | KBAR760-06 | MACN-Or-ct1372 | FJ028091 |
| *Picumnus cirratus* | KBAR907-06 | MACN-Or-ct1681 | FJ028092 |
| *Picumnus temminckii* | KBARG178-07 | MACN-Or-ct2038 | FJ028093 |
| *Picumnus temminckii* | KBARG311-07 | MACN-Or-ct2919 | FJ028094 |
| *Pionus maximiliani* | KAARG447-07 | MACN-Or-cp63 | FJ028095 |
| *Pionus maximiliani* | KAARG446-07 | MACN-Or-cp62 | FJ028096 |
| *Pipraeidea melanonota* | KBAR268-06 | MACN-Or-ct918 | FJ028097 |
| *Pipraeidea melanonota* | KBAR267-06 | MACN-Or-ct1147 | FJ028098 |
| *Pipra fasciicauda* | KBARG306-07 | MACN-Or-ct2868 | FJ028099 |
| *Pipra fasciicauda* | KBARG406-07 | MACN-Or-ct2940 | FJ028100 |
| *Pipra fasciicauda* | KAARG538-07 | MACN-Or-ct3041 | FJ028101 |
| *Piranga flava* | KAARG177-07 | MACN-Or-ct2144 | FJ028102 |
| *Pitangus sulphuratus* | KBAR555-06 | MACN-Or-ct1504 | FJ028103 |
| *Pitangus sulphuratus* | KBAR381-06 | MACN-Or-ct063 | FJ028104 |
| *Pitangus sulphuratus* | KBARG320-07 | MACN-Or-ct2904 | FJ028105 |
| *Pitangus sulphuratus* | KBARG368-07 | MACN-Or-ct2913 | FJ028106 |
| *Pitangus sulphuratus* | KAARG551-07 | MACN-Or-ct3163 | FJ028107 |
| *Pitangus sulphuratus* | KBAR554-06 | MACN-Or-ct1501 | FJ028108 |
| *Pitangus sulphuratus* | KBAR888-06 | MACN-Or-ct1658 | FJ028109 |
| *Pitangus sulphuratus* | KAARG603-07 | MACN-Or-ct3231 | FJ028110 |
| *Platalea ajaja* | KBARG024-07 | MACN-Or-ct1761 | FJ028111 |
| *Platalea ajaja* | KBARG056-07 | MACN-Or-ct1794 | FJ028112 |
| *Platyrinchus mystaceus* | KBARG347-07 | MACN-Or-ct2859 | FJ028113 |
| *Platyrinchus mystaceus* | KBARG384-07 | MACN-Or-ct2966 | FJ028114 |
| *Platyrinchus mystaceus* | KAARG536-07 | MACN-Or-ct2960 | FJ028115 |
| *Plegadis chihi* | KBAR579-06 | MACN-Or-ct1536 | FJ028116 |
| *Plegadis chihi* | KBAR573-06 | MACN-Or-ct1529 | FJ028117 |
| *Plegadis chihi* | KBAR860-06 | MACN-Or-ct1621 | FJ028118 |
| *Pluvialis dominica* | KAARG440-07 | MACN-Or-cp56 | FJ028119 |
| *Pluvianellus socialis* | KAARG023-07 | MACN-Or-ct211 | FJ028120 |
| *Pluvianellus socialis* | KAARG022-07 | MACN-Or-ct210 | FJ028121 |
| *Podager nacunda* | KBAR493-06 | MACN-Or-ct1396 | FJ028122 |
| *Podager nacunda* | KBAR494-06 | MACN-Or-ct1397 | FJ028123 |
| *Podager nacunda* | KBAR838-06 | MACN-Or-ct1591 | FJ028124 |
| *Podiceps gallardoi* | KBAR942-06 | PG2 | FJ028125 |
| *Podiceps gallardoi* | KBAR943-06 | PG3 | FJ028126 |
| *Podiceps major* | KAARG157-07 | MACN-Or-ct2123 | FJ028127 |
| *Podilymbus podiceps* | KBAR380-06 | MACN-Or-ct062 | FJ028128 |
| *Polioptila dumicola* | KAARG080-07 | MACN-Or-ct1901 | FJ028129 |
| *Polioptila dumicola* | KBAR825-06 | MACN-Or-ct1570 | FJ028130 |
| *Polioptila dumicola* | KBAR826-06 | MACN-Or-ct1573 | FJ028131 |
| *Polioptila dumicola* | KBAR828-06 | MACN-Or-ct1576 | FJ028132 |
| *Polioptila dumicola* | KAARG607-07 | MACN-Or-ct3236 | FJ028133 |
| *Polioptila dumicola* | KAARG198-07 | MACN-Or-ct2167 | FJ028134 |
| *Polioptila dumicola* | KAARG202-07 | MACN-Or-ct2172 | FJ028135 |
| *Poospiza baeri* | KBAR317-06 | MACN-Or-ct828 | FJ028136 |
| *Poospiza baeri* | KBAR269-06 | MACN-Or-ct1006 | FJ028137 |
| *Poospiza baeri* | KBAR270-06 | MACN-Or-ct1008 | FJ028138 |
| *Poospiza erythrophrys* | KBAR272-06 | MACN-Or-ct709 | FJ028139 |
| *Poospiza erythrophrys* | KBAR273-06 | MACN-Or-ct714 | FJ028140 |
| *Poospiza erythrophrys* | KBAR337-06 | MACN-Or-ct920 | FJ028141 |
| *Poospiza hypochondria* | KBAR318-06 | MACN-Or-ct831 | FJ028142 |
| *Poospiza hypochondria* | KBAR277-06 | MACN-Or-ct1061 | FJ028143 |
| *Poospiza hypochondria* | KBAR275-06 | MACN-Or-ct1010 | FJ028144 |
| *Poospiza melanoleuca* | KBAR868-06 | MACN-Or-ct1632 | FJ028145 |
| *Poospiza melanoleuca* | KBAR876-06 | MACN-Or-ct1644 | FJ028146 |
| *Poospiza melanoleuca* | KAARG564-07 | MACN-Or-ct3178 | FJ028147 |
| *Poospiza melanoleuca* | KAARG567-07 | MACN-Or-ct3182 | FJ028148 |
| *Poospiza nigrorufa* | KAARG165-07 | MACN-Or-ct2132 | FJ028149 |
| *Poospiza nigrorufa* | KAARG211-07 | MACN-Or-ct2182 | FJ028150 |
| *Poospiza nigrorufa* | KAARG153-07 | MACN-Or-ct2119 | FJ028151 |
| *Poospiza ornata* | KBAR384-06 | MACN-Or-ct190 | FJ028152 |
| *Poospiza ornata* | KBARG273-07 | MACN-Or-ct2795 | FJ028153 |
| *Poospiza ornata* | KBARG279-07 | MACN-Or-ct2750 | FJ028154 |
| *Poospiza torquata* | KBARG216-07 | MACN-Or-ct2755 | FJ028155 |
| *Poospiza torquata* | KBARG293-07 | MACN-Or-ct2837 | FJ028156 |
| *Poospiza torquata* | KBARG317-07 | MACN-Or-ct2824 | FJ028157 |
| *Porphyrio flavirostris* | KAARG454-07 | MACN-Or-cp70 | FJ028158 |
| *Psarocolius decumanus* | KBAR299-06 | MACN-Or-ct1130 | FJ028159 |
| *Pseudocolopteryx flaviventris* | KAARG161-07 | MACN-Or-ct2128 | FJ028160 |
| *Pseudocolopteryx sclateri* | KBAR586-06 | MACN-Or-ct1546 | FJ028161 |
| *Pseudocolopteryx sclateri* | KBAR587-06 | MACN-Or-ct1547 | FJ028162 |
| *Pseudoleistes guirahuro* | KAARG434-07 | MACN-Or-cp50 | FJ028163 |
| *Pseudoleistes virescens* | KAARG147-07 | MACN-Or-ct2113 | FJ028164 |
| *Pseudoleistes virescens* | KAARG176-07 | MACN-Or-ct2143 | FJ028165 |
| *Pseudoscops clamator* | KBAR613-06 | MACN-Or-ct206 | FJ028166 |
| *Pseudoseisura gutturalis* | KBARG222-07 | MACN-Or-ct2713 | FJ028167 |
| *Pseudoseisura gutturalis* | KBARG230-07 | MACN-Or-ct2716 | FJ028168 |
| *Pseudoseisura gutturalis* | KBARG238-07 | MACN-Or-ct2721 | FJ028169 |
| *Pseudoseisura gutturalis* | KAARG324-07 | MACN-Or-ct3000 | FJ028170 |
| *Pseudoseisura gutturalis* | KAARG336-07 | MACN-Or-ct3016 | FJ028171 |
| *Psilopsiagon aurifrons* | KBAR664-06 | MACN-Or-ct848 | FJ028172 |
| *Psilopsiagon aurifrons* | KBAR665-06 | MACN-Or-ct853 | FJ028173 |
| *Psilopsiagon aymara* | KBAR661-06 | MACN-Or-ct819 | FJ028174 |
| *Psilopsiagon aymara* | KBAR682-06 | MACN-Or-ct1019 | FJ028175 |
| *Psilopsiagon aymara* | KBAR683-06 | MACN-Or-ct1020 | FJ028176 |
| *Pteroglossus castanotis* | KBARG337-07 | MACN-Or-ct2892 | FJ028177 |
| *Pteroglossus castanotis* | KBARG299-07 | MACN-Or-ct2853 | FJ028178 |
| *Pteroptochos tarnii* | KBARG235-07 | MACN-Or-ct2629 | FJ028179 |
| *Pygarrhichas albogularis* | KBARG269-07 | MACN-Or-ct2686 | FJ028180 |
| *Pygarrhichas albogularis* | KBAR070-06 | MACN-Or-ct616 | FJ028181 |
| *Pygarrhichas albogularis* | KBAR071-06 | MACN-Or-ct614 | FJ028182 |
| *Pygarrhichas albogularis* | KAARG343-07 | MACN-Or-ct3025 | FJ028183 |
| *Pygarrhichas albogularis* | KAARG347-07 | MACN-Or-ct3032 | FJ028184 |
| *Pygoscelis papua* | KBAR394-06 | MACN-Or-ct386 | FJ028185 |
| *Pyriglena leucoptera* | KBARG156-07 | MACN-Or-ct2027 | FJ028186 |
| *Pyriglena leucoptera* | KBARG108-07 | MACN-Or-ct1959 | FJ028187 |
| *Pyriglena leucoptera* | KBARG116-07 | MACN-Or-ct1973 | FJ028188 |
| *Pyrocephalus rubinus* | KBAR368-06 | MACN-Or-ct037 | FJ028189 |
| *Pyrocephalus rubinus* | KBAR378-06 | MACN-Or-ct054 | FJ028190 |
| *Pyrocephalus rubinus* | KBARG064-07 | MACN-Or-ct1775 | FJ028191 |
| *Pyrocephalus rubinus* | KBARG333-07 | MACN-Or-ct2827 | FJ028192 |
| *Pyrocephalus rubinus* | KBAR490-06 | MACN-Or-ct1390 | FJ028193 |
| *Pyrocephalus rubinus* | KAARG278-07 | MACN-Or-ct2369 | FJ028194 |
| *Pyrrhocoma ruficeps* | KBARG107-07 | MACN-Or-ct1976 | FJ028195 |
| *Pyrrhocoma ruficeps* | KBARG408-07 | MACN-Or-ct2972 | FJ028196 |
| *Pyrrhomyias cinnamomeus* | KBAR162-06 | MACN-Or-ct748 | FJ028197 |
| *Pyrrhura molinae* | KBAR651-06 | MACN-Or-ct733 | FJ028198 |
| *Pyrrhura molinae* | KBAR692-06 | MACN-Or-ct1117 | FJ028199 |
| *Ramphastos dicolorus* | KBARG378-07 | MACN-Or-ct3051 | FJ028200 |
| *Ramphastos toco* | KBARG419-07 | MACN-Or-ct3049 | FJ028201 |
| *Recurvirostra andina* | KBAR006-06 | MACN-Or-ct1190 | FJ028202 |
| *Recurvirostra andina* | KBAR005-06 | MACN-Or-ct864 | FJ028203 |
| *Recurvirostra andina* | KBAR739-06 | MACN-Or-ct1296 | FJ028204 |
| *Recurvirostra andina* | KBAR740-06 | MACN-Or-ct1297 | FJ028205 |
| *Rhynchotus rufescens* | KAARG094-07 | MACN-Or-ct1917 | FJ028206 |
| *Rhynchotus rufescens* | KAARG116-07 | MACN-Or-ct2062 | FJ028207 |
| *Rhynchotus rufescens* | KAARG123-07 | MACN-Or-ct2073 | FJ028208 |
| *Rhynchotus rufescens* | KAARG124-07 | MACN-Or-ct2074 | FJ028209 |
| *Rollandia rolland* | KAARG195-07 | MACN-Or-ct2163 | FJ028210 |
| *Rostratula semicollaris* | KBAR564-06 | MACN-Or-ct1517 | FJ028211 |
| *Rostratula semicollaris* | KBAR566-06 | MACN-Or-ct1519 | FJ028212 |
| *Rostratula semicollaris* | KBAR585-06 | MACN-Or-ct1545 | FJ028213 |
| *Rostrhamus sociabilis* | KBAR783-06 | MACN-Or-ct1440 | FJ028214 |
| *Rostrhamus sociabilis* | KBAR810-06 | MACN-Or-ct1553 | FJ028215 |
| *Rynchops niger* | KAARG119-07 | MACN-Or-ct2068 | FJ028216 |
| *Rynchops niger* | KAARG120-07 | MACN-Or-ct2069 | FJ028217 |
| *Rynchops niger* | KAARG121-07 | MACN-Or-ct2070 | FJ028218 |
| *Saltator aurantiirostris* | KBARG249-07 | MACN-Or-ct2787 | FJ028219 |
| *Saltator aurantiirostris* | KAARG589-07 | MACN-Or-ct3214 | FJ028220 |
| *Saltator aurantiirostris* | KBAR278-06 | MACN-Or-ct1003 | FJ028221 |
| *Saltator aurantiirostris* | KAARG104-07 | MACN-Or-ct1928 | FJ028222 |
| *Saltator aurantiirostris* | KAARG105-07 | MACN-Or-ct1929 | FJ028223 |
| *Saltator aurantiirostris* | KAARG297-07 | MACN-Or-ct2411 | FJ028224 |
| *Saltator aurantiirostris* | KAARG308-07 | MACN-Or-ct2790 | FJ028225 |
| *Saltator coerulescens* | KBARG040-07 | MACN-Or-ct295 | FJ028226 |
| *Saltator coerulescens* | KAARG568-07 | MACN-Or-ct3183 | FJ028227 |
| *Saltator coerulescens* | KBAR477-06 | MACN-Or-ct1350 | FJ028228 |
| *Saltator coerulescens* | KBAR483-06 | MACN-Or-ct1364 | FJ028229 |
| *Saltator coerulescens* | KAARG569-07 | MACN-Or-ct3184 | FJ028230 |
| *Saltator similis* | KBARG361-07 | MACN-Or-ct2895 | FJ028231 |
| *Saltator similis* | KBAR500-06 | MACN-Or-ct1407 | FJ028232 |
| *Saltator similis* | KAARG295-07 | MACN-Or-ct2397 | FJ028233 |
| *Sappho sparganura* | KBAR650-06 | MACN-Or-ct725 | FJ028234 |
| *Sappho sparganura* | KBAR657-06 | MACN-Or-ct792 | FJ028235 |
| *Sappho sparganura* | KBAR669-06 | MACN-Or-ct935 | FJ028236 |
| *Sarkidiornis melanotos* | KBAR940-06 | MACN-Or-ct1721 | FJ028237 |
| *Satrapa icterophrys* | KBAR166-06 | MACN-Or-ct917 | FJ028238 |
| *Satrapa icterophrys* | KBAR165-06 | MACN-Or-ct1158 | FJ028239 |
| *Satrapa icterophrys* | KBAR759-06 | MACN-Or-ct1369 | FJ028240 |
| *Satrapa icterophrys* | KBAR905-06 | MACN-Or-ct1679 | FJ028241 |
| *Satrapa icterophrys* | KAARG160-07 | MACN-Or-ct2127 | FJ028242 |
| *Scelorchilus rubecula* | KBARG259-07 | MACN-Or-ct2634 | FJ028243 |
| *Scelorchilus rubecula* | KBARG266-07 | MACN-Or-ct2618 | FJ028244 |
| *Schiffornis virescens* | KBARG388-07 | MACN-Or-ct2967 | FJ028245 |
| *Schiffornis virescens* | KBARG412-07 | MACN-Or-ct2973 | FJ028246 |
| *Schiffornis virescens* | KBARG185-07 | MACN-Or-ct1960 | FJ028247 |
| *Schoeniophylax phryganophilus* | KAARG188-07 | MACN-Or-ct2156 | FJ028248 |
| *Schoeniophylax phryganophilus* | KBAR538-06 | MACN-Or-ct1477 | FJ028249 |
| *Schoeniophylax phryganophilus* | KBAR823-06 | MACN-Or-ct1568 | FJ028250 |
| *Schoeniophylax phryganophilus* | KAARG628-07 | MACN-Or-ct3371 | FJ028251 |
| *Scytalopus magellanicus* | KBARG267-07 | MACN-Or-ct2635 | FJ028252 |
| *Scytalopus magellanicus* | KBARG189-07 | MACN-Or-ct2636 | FJ028253 |
| *Scytalopus magellanicus* | KBARG141-07 | MACN-Or-ct2574 | FJ028254 |
| *Scytalopus superciliaris* | KBAR097-06 | MACN-Or-ct939 | FJ028255 |
| *Scytalopus superciliaris* | KBAR096-06 | MACN-Or-ct786 | FJ028256 |
| *Scytalopus superciliaris* | KBAR095-06 | MACN-Or-ct729 | FJ028257 |
| *Sephanoides sephaniodes* | KBAR641-06 | MACN-Or-ct610 | FJ028258 |
| *Sephanoides sephaniodes* | KBAR646-06 | MACN-Or-ct701 | FJ028259 |
| *Sephanoides sephaniodes* | KBAR647-06 | MACN-Or-ct702 | FJ028260 |
| *Serpophaga subcristata* | KBARG079-07 | MACN-Or-ct1780 | FJ028261 |
| *Serpophaga subcristata* | KBARG287-07 | MACN-Or-ct2801 | FJ028262 |
| *Serpophaga subcristata* | KBAR363-06 | MACN-Or-ct026 | FJ028263 |
| *Serpophaga subcristata* | KBAR895-06 | MACN-Or-ct1666 | FJ028264 |
| *Serpophaga subcristata* | KAARG307-07 | MACN-Or-ct2774 | FJ028265 |
| *Sicalis flaveola* | KBAR625-06 | MACN-Or-ct347 | FJ028266 |
| *Sicalis flaveola* | KAARG602-07 | MACN-Or-ct3230 | FJ028267 |
| *Sicalis flaveola* | KBAR281-06 | MACN-Or-ct634 | FJ028268 |
| *Sicalis flaveola* | KBAR624-06 | MACN-Or-ct345 | FJ028269 |
| *Sicalis flaveola* | KBAR785-06 | MACN-Or-ct1442 | FJ028270 |
| *Sicalis flaveola* | KBAR786-06 | MACN-Or-ct1443 | FJ028271 |
| *Sicalis luteocephala* | KBAR391-06 | MACN-Or-ct354 | FJ028272 |
| *Sicalis luteola* | KBAR811-06 | MACN-Or-ct1554 | FJ028273 |
| *Sicalis luteola* | KBAR283-06 | MACN-Or-ct627 | FJ028274 |
| *Sicalis luteola* | KBAR282-06 | MACN-Or-ct561 | FJ028275 |
| *Sicalis luteola* | KBAR310-06 | MACN-Or-ct674 | FJ028276 |
| *Sicalis luteola* | KBAR600-06 | MACN-Or-ct017 | FJ028277 |
| *Sicalis luteola* | KBAR816-06 | MACN-Or-ct1560 | FJ028278 |
| *Sicalis luteola* | KBARG358-07 | MACN-Or-ct2818 | FJ028279 |
| *Sicalis olivascens* | KAARG051-07 | MACN-Or-ct822 | FJ028280 |
| *Sicalis olivascens* | KAARG055-07 | MACN-Or-ct1303 | FJ028281 |
| *Sicalis olivascens* | KBAR285-06 | MACN-Or-ct1077 | FJ028282 |
| *Sicalis olivascens* | KBAR280-06 | MACN-Or-ct1054 | FJ028283 |
| *Sicalis olivascens* | KBAR279-06 | MACN-Or-ct1025 | FJ028284 |
| *Sicalis olivascens* | KBAR744-06 | MACN-Or-ct1307 | FJ028285 |
| *Sicalis olivascens* | KBAR745-06 | MACN-Or-ct1311 | FJ028286 |
| *Sittasomus griseicapillus* | KAARG556-07 | MACN-Or-ct3169 | FJ028287 |
| *Sittasomus griseicapillus* | KAARG593-07 | MACN-Or-ct3219 | FJ028288 |
| *Sittasomus griseicapillus* | KBAR084-06 | MACN-Or-ct1124 | FJ028289 |
| *Sittasomus griseicapillus* | KBAR085-06 | MACN-Or-ct907 | FJ028290 |
| *Sittasomus griseicapillus* | KBAR086-06 | MACN-Or-ct908 | FJ028291 |
| *Sittasomus griseicapillus* | KBARG099-07 | MACN-Or-ct1970 | FJ028292 |
| *Sittasomus griseicapillus* | KAARG293-07 | MACN-Or-ct2392 | FJ028293 |
| *Spartonoica maluroides* | KBAR412-06 | MACN-Or-ct493 | FJ028294 |
| *Spheniscus magellanicus* | KBARG029-07 | MACN-Or-ct1345 | FJ028295 |
| *Spheniscus magellanicus* | KBARG061-07 | MACN-Or-ct1344 | FJ028296 |
| *Spheniscus magellanicus* | KBARG092-07 | MACN-Or-ct1346 | FJ028297 |
| *Sporophila caerulescens* | KBAR481-06 | MACN-Or-ct1358 | FJ028298 |
| *Sporophila caerulescens* | KBAR527-06 | MACN-Or-ct1462 | FJ028299 |
| *Sporophila caerulescens* | KBAR578-06 | MACN-Or-ct1535 | FJ028300 |
| *Sporophila caerulescens* | KAARG366-07 | MACN-Or-ct3192 | FJ028301 |
| *Sporophila caerulescens* | KAARG368-07 | MACN-Or-ct3211 | FJ028302 |
| *Sporophila caerulescens* | KAARG372-07 | MACN-Or-ct3232 | FJ028303 |
| *Sporophila cinnamomea* | KAARG383-07 | MACN-Or-ct3122 | FJ028304 |
| *Sporophila cinnamomea* | KAARG382-07 | MACN-Or-ct3121 | FJ028305 |
| *Sporophila collaris* | KBAR908-06 | MACN-Or-ct1682 | FJ028306 |
| *Sporophila collaris* | KAARG367-07 | MACN-Or-ct3202 | FJ028307 |
| *Sporophila collaris* | KBAR911-06 | MACN-Or-ct1685 | FJ028308 |
| *Sporophila collaris* | KBAR909-06 | MACN-Or-ct1683 | FJ028309 |
| *Sporophila collaris* | KAARG365-07 | MACN-Or-ct3185 | FJ028310 |
| *Sporophila hypochroma* | KAARG387-07 | MACN-Or-ct3131 | FJ028311 |
| *Sporophila hypoxantha* | KAARG363-07 | MACN-Or-ct3073 | FJ028312 |
| *Sporophila hypoxantha* | KAARG373-07 | MACN-Or-ct3252 | FJ028313 |
| *Sporophila hypoxantha* | KAARG374-07 | MACN-Or-ct3253 | FJ028314 |
| *Sporophila hypoxantha* | KAARG375-07 | MACN-Or-ct3272 | FJ028315 |
| *Sporophila leucoptera* | KAARG369-07 | MACN-Or-ct3212 | FJ028316 |
| *Sporophila palustris* | KAARG380-07 | MACN-Or-ct3117 | FJ028317 |
| *Sporophila palustris* | KAARG388-07 | MACN-Or-ct3372 | FJ028318 |
| *Sporophila ruficollis* | KAARG134-07 | MACN-Or-ct2088 | FJ028319 |
| *Sporophila ruficollis* | KAARG132-07 | MACN-Or-ct2086 | FJ028320 |
| *Sporophila ruficollis* | KAARG386-07 | MACN-Or-ct3130 | FJ028321 |
| *Sporophila zelichi* | KAARG516-07 | MACN-Or-ct3132 | FJ028322 |
| *Stephanoxis lalandi* | KBARG369-07 | MACN-Or-ct2896 | FJ028323 |
| *Stercorarius antarcticus* | KAARG003-07 | MACN-Or-ct127 | FJ028324 |
| *Sterna trudeaui* | KAARG034-07 | MACN-Or-ct301 | FJ028325 |
| *Sterna trudeaui* | KAARG035-07 | MACN-Or-ct302 | FJ028326 |
| *Sternula superciliaris* | KBARG003-07 | MACN-Or-ct1768 | FJ028327 |
| *Sternula superciliaris* | KBARG054-07 | MACN-Or-ct1782 | FJ028328 |
| *Stigmatura budytoides* | KBARG294-07 | MACN-Or-ct2802 | FJ028329 |
| *Stigmatura budytoides* | KBARG201-07 | MACN-Or-ct2752 | FJ028330 |
| *Stigmatura budytoides* | KBAR388-06 | MACN-Or-ct281 | FJ028331 |
| *Strix rufipes* | KBARG227-07 | MACN-Or-ct2628 | FJ028332 |
| *Sturnella loyca* | KBAR611-06 | MACN-Or-ct056 | FJ028333 |
| *Sturnella loyca* | KAARG355-07 | MACN-Or-ct3063 | FJ028334 |
| *Sturnella loyca* | KBAR300-06 | MACN-Or-ct549 | FJ028335 |
| *Sturnella loyca* | KBAR302-06 | MACN-Or-ct678 | FJ028336 |
| *Sturnella loyca* | KBAR301-06 | MACN-Or-ct597 | FJ028337 |
| *Sturnella superciliaris* | KBAR531-06 | MACN-Or-ct1467 | FJ028338 |
| *Sturnella superciliaris* | KBAR553-06 | MACN-Or-ct1500 | FJ028339 |
| *Sturnella superciliaris* | KBAR529-06 | MACN-Or-ct1465 | FJ028340 |
| *Sublegatus modestus* | KBAR356-06 | MACN-Or-ct005 | FJ028341 |
| *Sublegatus modestus* | KBAR390-06 | MACN-Or-ct325 | FJ028342 |
| *Suiriri suiriri* | KAARG174-07 | MACN-Or-ct2141 | FJ028343 |
| *Suiriri suiriri* | KBAR896-06 | MACN-Or-ct1667 | FJ028344 |
| *Suiriri suiriri* | KBAR897-06 | MACN-Or-ct1668 | FJ028345 |
| *Sylviorthorhynchus desmursii* | KBARG228-07 | MACN-Or-ct2646 | FJ028346 |
| *Sylviorthorhynchus desmursii* | KBARG251-07 | MACN-Or-ct2632 | FJ028347 |
| *Sylviorthorhynchus desmursii* | KAARG348-07 | MACN-Or-ct3033 | FJ028348 |
| *Synallaxis albescens* | KBARG342-07 | MACN-Or-ct2810 | FJ028349 |
| *Synallaxis albescens* | KBARG350-07 | MACN-Or-ct2817 | FJ028350 |
| *Synallaxis albescens* | KAARG587-07 | MACN-Or-ct3208 | FJ028351 |
| *Synallaxis albescens* | KBAR910-06 | MACN-Or-ct1684 | FJ028352 |
| *Synallaxis albescens* | KBAR546-06 | MACN-Or-ct1492 | FJ028353 |
| *Synallaxis albescens* | KBAR813-06 | MACN-Or-ct1556 | FJ028354 |
| *Synallaxis azarae* | KBAR073-06 | MACN-Or-ct760 | FJ028355 |
| *Synallaxis azarae* | KBAR072-06 | MACN-Or-ct739 | FJ028356 |
| *Synallaxis azarae* | KBAR074-06 | MACN-Or-ct996 | FJ028357 |
| *Synallaxis frontalis* | KBAR522-06 | MACN-Or-ct1450 | FJ028358 |
| *Synallaxis frontalis* | KAARG571-07 | MACN-Or-ct3187 | FJ028359 |
| *Synallaxis frontalis* | KAARG423-07 | MACN-Or-cp39 | FJ028360 |
| *Synallaxis frontalis* | KBAR866-06 | MACN-Or-ct1629 | FJ028361 |
| *Synallaxis spixi* | KAARG246-07 | MACN-Or-ct2279 | FJ028362 |
| *Synallaxis spixi* | KAARG247-07 | MACN-Or-ct2280 | FJ028363 |
| *Syndactyla rufosuperciliata* | KBARG155-07 | MACN-Or-ct1990 | FJ028364 |
| *Syndactyla rufosuperciliata* | KBAR077-06 | MACN-Or-ct724 | FJ028365 |
| *Syndactyla rufosuperciliata* | KBAR075-06 | MACN-Or-ct1116 | FJ028366 |
| *Syndactyla rufosuperciliata* | KBAR330-06 | MACN-Or-ct894 | FJ028367 |
| *Syndactyla rufosuperciliata* | KBAR889-06 | MACN-Or-ct1659 | FJ028368 |
| *Syrigma sibilatrix* | KBAR623-06 | MACN-Or-ct304 | FJ028369 |
| *Syrigma sibilatrix* | KBAR805-06 | MACN-Or-ct1521 | FJ028370 |
| *Syrigma sibilatrix* | KBAR836-06 | MACN-Or-ct1589 | FJ028371 |
| *Syrigma sibilatrix* | KBAR862-06 | MACN-Or-ct1623 | FJ028372 |
| *Tachuris rubrigastra* | KBAR172-06 | MACN-Or-ct692 | FJ028373 |
| *Tachuris rubrigastra* | KBAR170-06 | MACN-Or-ct598 | FJ028374 |
| *Tachuris rubrigastra* | KBAR171-06 | MACN-Or-ct675 | FJ028375 |
| *Tachycineta leucorrhoa* | KBARG119-07 | MACN-Or-ct1840 | FJ028376 |
| *Tachycineta leucorrhoa* | KBAR559-06 | MACN-Or-ct1512 | FJ028377 |
| *Tachycineta leucorrhoa* | KBAR560-06 | MACN-Or-ct1513 | FJ028378 |
| *Tachycineta meyeni* | KBARG253-07 | MACN-Or-ct2683 | FJ028379 |
| *Tachycineta meyeni* | KBARG261-07 | MACN-Or-ct2684 | FJ028380 |
| *Tachycineta meyeni* | KAARG031-07 | MACN-Or-ct287 | FJ028381 |
| *Tachycineta meyeni* | KBAR440-06 | MACN-Or-ct639 | FJ028382 |
| *Tachyphonus coronatus* | KBARG170-07 | MACN-Or-ct1952 | FJ028383 |
| *Tachyphonus coronatus* | KBARG283-07 | MACN-Or-ct2864 | FJ028384 |
| *Tachyphonus rufus* | KBAR927-06 | MACN-Or-ct1703 | FJ028385 |
| *Tachyphonus rufus* | KAARG542-07 | MACN-Or-ct3147 | FJ028386 |
| *Tachyphonus rufus* | KBAR562-06 | MACN-Or-ct1515 | FJ028387 |
| *Tachyphonus rufus* | KBAR856-06 | MACN-Or-ct1617 | FJ028388 |
| *Tachyphonus rufus* | KAARG599-07 | MACN-Or-ct3227 | FJ028389 |
| *Tapera naevia* | KBARG137-07 | MACN-Or-ct1877 | FJ028390 |
| *Tapera naevia* | KBARG145-07 | MACN-Or-ct1878 | FJ028391 |
| *Tapera naevia* | KAARG269-07 | MACN-Or-ct2357 | FJ028392 |
| *Tapera naevia* | KBAR489-06 | MACN-Or-ct1380 | FJ028393 |
| *Taraba major* | KAARG584-07 | MACN-Or-ct3203 | FJ028394 |
| *Taraba major* | KAARG547-07 | MACN-Or-ct3156 | FJ028395 |
| *Taraba major* | KAARG598-07 | MACN-Or-ct3226 | FJ028396 |
| *Thalassarche chlororhynchos* | KBARG059-07 | MACN-Or-ct1347 | FJ028397 |
| *Thalasseus maximus* | KAARG018-07 | MACN-Or-ct187 | FJ028398 |
| *Thalasseus maximus* | KAARG017-07 | MACN-Or-ct186 | FJ028399 |
| *Thalasseus sandvicensis* | KAARG013-07 | MACN-Or-ct167 | FJ028400 |
| *Thalasseus sandvicensis* | KAARG014-07 | MACN-Or-ct168 | FJ028401 |
| *Thalurania glaucopis* | KBARG321-07 | MACN-Or-ct2889 | FJ028402 |
| *Thalurania glaucopis* | KBARG344-07 | MACN-Or-ct2907 | FJ028403 |
| *Thalurania glaucopis* | KBARG362-07 | MACN-Or-ct2881 | FJ028404 |
| *Thamnophilus caerulescens* | KBAR767-06 | MACN-Or-ct1387 | FJ028405 |
| *Thamnophilus caerulescens* | KBAR091-06 | MACN-Or-ct1128 | FJ028406 |
| *Thamnophilus caerulescens* | KBAR092-06 | MACN-Or-ct892 | FJ028407 |
| *Thamnophilus caerulescens* | KAARG557-07 | MACN-Or-ct3170 | FJ028408 |
| *Thamnophilus caerulescens* | KBAR779-06 | MACN-Or-ct1425 | FJ028409 |
| *Thamnophilus caerulescens* | KBAR867-06 | MACN-Or-ct1630 | FJ028410 |
| *Thamnophilus caerulescens* | KAARG617-07 | MACN-Or-ct3251 | FJ028411 |
| *Thamnophilus ruficapillus* | KBAR093-06 | MACN-Or-ct1132 | FJ028412 |
| *Thamnophilus ruficapillus* | KBAR094-06 | MACN-Or-ct885 | FJ028413 |
| *Thamnophilus ruficapillus* | KAARG155-07 | MACN-Or-ct2121 | FJ028414 |
| *Thamnophilus ruficapillus* | KAARG216-07 | MACN-Or-ct2188 | FJ028415 |
| *Theristicus melanopis* | KBAR425-06 | MACN-Or-ct538 | FJ028416 |
| *Theristicus melanopis* | KBAR426-06 | MACN-Or-ct539 | FJ028417 |
| *Thinocorus orbignyianus* | KBAR016-06 | MACN-Or-ct865 | FJ028418 |
| *Thinocorus orbignyianus* | KBAR015-06 | MACN-Or-ct1180 | FJ028419 |
| *Thinocorus orbignyianus* | KBAR014-06 | MACN-Or-ct1103 | FJ028420 |
| *Thinocorus orbignyianus* | KBAR013-06 | MACN-Or-ct1102 | FJ028421 |
| *Thinocorus orbignyianus* | KBAR748-06 | MACN-Or-ct1319 | FJ028422 |
| *Thinocorus orbignyianus* | KBAR749-06 | MACN-Or-ct1321 | FJ028423 |
| *Thlypopsis ruficeps* | KBAR288-06 | MACN-Or-ct926 | FJ028424 |
| *Thlypopsis ruficeps* | KBAR286-06 | MACN-Or-ct734 | FJ028425 |
| *Thlypopsis ruficeps* | KBAR287-06 | MACN-Or-ct747 | FJ028426 |
| *Thlypopsis sordida* | KBAR495-06 | MACN-Or-ct1399 | FJ028427 |
| *Thlypopsis sordida* | KBARG020-07 | MACN-Or-ct1804 | FJ028428 |
| *Thlypopsis sordida* | KBARG081-07 | MACN-Or-ct1811 | FJ028429 |
| *Thraupis bonariensis* | KBAR289-06 | MACN-Or-ct1106 | FJ028430 |
| *Thraupis bonariensis* | KBAR290-06 | MACN-Or-ct871 | FJ028431 |
| *Thraupis bonariensis* | KBAR597-06 | MACN-Or-ct001 | FJ028432 |
| *Thraupis bonariensis* | KAARG309-07 | MACN-Or-ct2815 | FJ028433 |
| *Thraupis bonariensis* | KBARG039-07 | MACN-Or-ct1726 | FJ028434 |
| *Thraupis bonariensis* | KBARG209-07 | MACN-Or-ct2753 | FJ028435 |
| *Thraupis bonariensis* | KAARG310-07 | MACN-Or-ct2816 | FJ028436 |
| *Thraupis sayaca* | KBAR291-06 | MACN-Or-ct1159 | FJ028437 |
| *Thraupis sayaca* | KBAR626-06 | MACN-Or-ct361 | FJ028438 |
| *Thraupis sayaca* | KBAR770-06 | MACN-Or-ct1394 | FJ028439 |
| *Thraupis sayaca* | KBARG015-07 | MACN-Or-ct1727 | FJ028440 |
| *Thraupis sayaca* | KAARG095-07 | MACN-Or-ct1918 | FJ028441 |
| *Tigrisoma lineatum* | KBAR863-06 | MACN-Or-ct1625 | FJ028442 |
| *Tigrisoma lineatum* | KBAR881-06 | MACN-Or-ct1649 | FJ028443 |
| *Tinamotis ingoufi* | KBAR382-06 | MACN-Or-ct113 | FJ028444 |
| *Tinamotis ingoufi* | KAARG263-07 | MACN-Or-ct2335 | FJ028445 |
| *Tinamotis pentlandii* | KBAR003-06 | MACN-Or-ct1202 | FJ028446 |
| *Tinamotis pentlandii* | KBAR004-06 | MACN-Or-ct1203 | FJ028447 |
| *Tinamotis pentlandii* | KBAR002-06 | MACN-Or-ct1201 | FJ028448 |
| *Trichothraupis melanops* | KBARG284-07 | MACN-Or-ct2851 | FJ028449 |
| *Trichothraupis melanops* | KBARG324-07 | MACN-Or-ct2843 | FJ028450 |
| *Trichothraupis melanops* | KBARG177-07 | MACN-Or-ct1953 | FJ028451 |
| *Tringa flavipes* | KBARG007-07 | MACN-Or-ct1749 | FJ028452 |
| *Tringa flavipes* | KBARG017-07 | MACN-Or-ct1743 | FJ028453 |
| *Tringa flavipes* | KBARG051-07 | MACN-Or-ct1737 | FJ028454 |
| *Tringa melanoleuca* | KAARG012-07 | MACN-Or-ct160 | FJ028455 |
| *Tringa solitaria* | KBARG021-07 | MACN-Or-ct1735 | FJ028456 |
| *Tringa solitaria* | KBARG070-07 | MACN-Or-ct1750 | FJ028457 |
| *Troglodytes aedon* | KBAR180-06 | MACN-Or-ct1016 | FJ028458 |
| *Troglodytes aedon* | KBAR182-06 | MACN-Or-ct695 | FJ028459 |
| *Troglodytes aedon* | KBAR181-06 | MACN-Or-ct599 | FJ028460 |
| *Troglodytes aedon* | KBAR610-06 | MACN-Or-ct055 | FJ028461 |
| *Troglodytes aedon* | KBAR794-06 | MACN-Or-ct1475 | FJ028462 |
| *Troglodytes aedon* | KBARG073-07 | MACN-Or-ct286 | FJ028463 |
| *Troglodytes aedon* | KBARG221-07 | MACN-Or-ct2679 | FJ028464 |
| *Troglodytes aedon* | KBARG229-07 | MACN-Or-ct2680 | FJ028465 |
| *Troglodytes aedon* | KBARG268-07 | MACN-Or-ct2666 | FJ028466 |
| *Troglodytes aedon* | KBARG345-07 | MACN-Or-ct2893 | FJ028467 |
| *Troglodytes aedon* | KBARG348-07 | MACN-Or-ct2846 | FJ028468 |
| *Troglodytes aedon* | KAARG081-07 | MACN-Or-ct1902 | FJ028469 |
| *Troglodytes aedon* | KAARG261-07 | MACN-Or-ct2333 | FJ028470 |
| *Troglodytes aedon* | KBARG136-07 | MACN-Or-ct1860 | FJ028471 |
| *Troglodytes aedon* | KAARG578-07 | MACN-Or-ct3196 | FJ028472 |
| *Troglodytes aedon* | KAARG076-07 | MACN-Or-ct1895 | FJ028473 |
| *Troglodytes aedon* | KAARG082-07 | MACN-Or-ct1903 | FJ028474 |
| *Troglodytes aedon* | KAARG299-07 | MACN-Or-ct2599 | FJ028475 |
| *Troglodytes aedon* | KAARG555-07 | MACN-Or-ct3168 | FJ028476 |
| *Troglodytes solstitialis* | KBAR183-06 | MACN-Or-ct765 | FJ028477 |
| *Troglodytes solstitialis* | KBAR184-06 | MACN-Or-ct778 | FJ028478 |
| *Troglodytes solstitialis* | KBAR185-06 | MACN-Or-ct785 | FJ028479 |
| *Trogon rufus* | KBARG340-07 | MACN-Or-ct2845 | FJ028480 |
| *Trogon surrucura* | KBAR929-06 | MACN-Or-ct1705 | FJ028481 |
| *Trogon surrucura* | KBAR871-06 | MACN-Or-ct1636 | FJ028482 |
| *Trogon surrucura* | KBAR872-06 | MACN-Or-ct1637 | FJ028483 |
| *Trogon surrucura* | KBARG376-07 | MACN-Or-ct2946 | FJ028484 |
| *Trogon surrucura* | KBARG382-07 | MACN-Or-ct2934 | FJ028485 |
| *Turdus albicollis* | KBARG332-07 | MACN-Or-ct2844 | FJ028486 |
| *Turdus albicollis* | KBARG392-07 | MACN-Or-ct2968 | FJ028487 |
| *Turdus albicollis* | KBARG352-07 | MACN-Or-ct2909 | FJ028488 |
| *Turdus amaurochalinus* | KBAR193-06 | MACN-Or-ct923 | FJ028489 |
| *Turdus amaurochalinus* | KBAR194-06 | MACN-Or-ct938 | FJ028490 |
| *Turdus amaurochalinus* | KBAR195-06 | MACN-Or-ct975 | FJ028491 |
| *Turdus amaurochalinus* | KBAR772-06 | MACN-Or-ct1398 | FJ028492 |
| *Turdus amaurochalinus* | KBAR807-06 | MACN-Or-ct1537 | FJ028493 |
| *Turdus amaurochalinus* | KAARG089-07 | MACN-Or-ct1910 | FJ028494 |
| *Turdus amaurochalinus* | KAARG203-07 | MACN-Or-ct2173 | FJ028495 |
| *Turdus amaurochalinus* | KAARG574-07 | MACN-Or-ct3190 | FJ028496 |
| *Turdus amaurochalinus* | KAARG047-07 | MACN-Or-ct366 | FJ028497 |
| *Turdus amaurochalinus* | KAARG290-07 | MACN-Or-ct2387 | FJ028498 |
| *Turdus chiguanco* | KBAR198-06 | MACN-Or-ct726 | FJ028499 |
| *Turdus chiguanco* | KBAR196-06 | MACN-Or-ct1056 | FJ028500 |
| *Turdus chiguanco* | KBAR314-06 | MACN-Or-ct798 | FJ028501 |
| *Turdus falcklandii* | KBAR427-06 | MACN-Or-ct540 | FJ028502 |
| *Turdus falcklandii* | KBAR428-06 | MACN-Or-ct543 | FJ028503 |
| *Turdus falcklandii* | KBARG212-07 | MACN-Or-ct2626 | FJ028504 |
| *Turdus falcklandii* | KBARG149-07 | MACN-Or-ct2579 | FJ028505 |
| *Turdus leucomelas* | KBARG304-07 | MACN-Or-ct2902 | FJ028506 |
| *Turdus leucomelas* | KBARG338-07 | MACN-Or-ct2872 | FJ028507 |
| *Turdus leucomelas* | KBARG364-07 | MACN-Or-ct2848 | FJ028508 |
| *Turdus nigriceps* | KBAR199-06 | MACN-Or-ct960 | FJ028509 |
| *Turdus rufiventris* | KBAR202-06 | MACN-Or-ct1127 | FJ028510 |
| *Turdus rufiventris* | KBAR351-06 | MACN-Or-ct1154 | FJ028511 |
| *Turdus rufiventris* | KBAR621-06 | MACN-Or-ct270 | FJ028512 |
| *Turdus rufiventris* | KBAR331-06 | MACN-Or-ct895 | FJ028513 |
| *Turdus rufiventris* | KBAR780-06 | MACN-Or-ct1428 | FJ028514 |
| *Turdus rufiventris* | KAARG550-07 | MACN-Or-ct3161 | FJ028515 |
| *Turdus rufiventris* | KBARG314-07 | MACN-Or-ct2869 | FJ028516 |
| *Turdus rufiventris* | KBARG330-07 | MACN-Or-ct2871 | FJ028517 |
| *Turdus rufiventris* | KAARG610-07 | MACN-Or-ct3241 | FJ028518 |
| *Turdus rufiventris* | KAARG071-07 | MACN-Or-ct1890 | FJ028519 |
| *Turdus rufiventris* | KAARG079-07 | MACN-Or-ct1899 | FJ028520 |
| *Tyrannus melancholicus* | KBAR173-06 | MACN-Or-ct1125 | FJ028521 |
| *Tyrannus melancholicus* | KBAR175-06 | MACN-Or-ct891 | FJ028522 |
| *Tyrannus melancholicus* | KBAR335-06 | MACN-Or-ct906 | FJ028523 |
| *Tyrannus melancholicus* | KBARG001-07 | MACN-Or-ct1774 | FJ028524 |
| *Tyrannus melancholicus* | KBARG062-07 | MACN-Or-ct1763 | FJ028525 |
| *Tyrannus savana* | KBARG058-07 | MACN-Or-ct1789 | FJ028526 |
| *Tyrannus savana* | KBARG255-07 | MACN-Or-ct2744 | FJ028527 |
| *Tyrannus savana* | KBARG025-07 | MACN-Or-ct1724 | FJ028528 |
| *Tyto alba* | KBARG278-07 | MACN-Or-ct2730 | FJ028529 |
| *Upucerthia dumetaria* | KBAR606-06 | MACN-Or-ct039 | FJ028530 |
| *Upucerthia dumetaria* | KBAR078-06 | MACN-Or-ct559 | FJ028531 |
| *Upucerthia dumetaria* | KBAR079-06 | MACN-Or-ct594 | FJ028532 |
| *Upucerthia dumetaria* | KBAR080-06 | MACN-Or-ct601 | FJ028533 |
| *Upucerthia dumetaria* | KBARG226-07 | MACN-Or-ct2609 | FJ028534 |
| *Upucerthia dumetaria* | KAARG300-07 | MACN-Or-ct2648 | FJ028535 |
| *Upucerthia dumetaria* | KAARG304-07 | MACN-Or-ct2714 | FJ028536 |
| *Upucerthia dumetaria* | KAARG305-07 | MACN-Or-ct2725 | FJ028537 |
| *Upucerthia dumetaria* | KAARG320-07 | MACN-Or-ct2994 | FJ028538 |
| *Upucerthia dumetaria* | KAARG327-07 | MACN-Or-ct3005 | FJ028539 |
| *Upucerthia dumetaria* | KAARG331-07 | MACN-Or-ct3011 | FJ028540 |
| *Upucerthia dumetaria* | KAARG334-07 | MACN-Or-ct3014 | FJ028541 |
| *Upucerthia ruficaudus* | KBAR081-06 | MACN-Or-ct1072 | FJ028542 |
| *Upucerthia ruficaudus* | KBAR083-06 | MACN-Or-ct1095 | FJ028543 |
| *Upucerthia ruficaudus* | KBAR082-06 | MACN-Or-ct1075 | FJ028544 |
| *Vanellus chilensis* | KBAR782-06 | MACN-Or-ct1432 | FJ028545 |
| *Vanellus chilensis* | KBAR777-06 | MACN-Or-ct1423 | FJ028546 |
| *Vanellus chilensis* | KAARG345-07 | MACN-Or-ct3028 | FJ028547 |
| *Vanellus chilensis* | KBAR010-06 | MACN-Or-ct578 | FJ028548 |
| *Vanellus chilensis* | KBAR011-06 | MACN-Or-ct587 | FJ028549 |
| *Vanellus chilensis* | KBAR903-06 | MACN-Or-ct1676 | FJ028550 |
| *Vanellus chilensis* | KAARG110-07 | MACN-Or-ct1938 | FJ028551 |
| *Vanellus chilensis* | KAARG328-07 | MACN-Or-ct3007 | FJ028552 |
| *Veniliornis frontalis* | KBAR034-06 | MACN-Or-ct970 | FJ028553 |
| *Veniliornis passerinus* | KBAR931-06 | MACN-Or-ct1709 | FJ028554 |
| *Veniliornis passerinus* | KBAR874-06 | MACN-Or-ct1640 | FJ028555 |
| *Veniliornis passerinus* | KAARG059-07 | MACN-Or-ct1707 | FJ028556 |
| *Vireo olivaceus* | KBAR206-06 | MACN-Or-ct1123 | FJ028557 |
| *Vireo olivaceus* | KBARG102-07 | MACN-Or-ct1815 | FJ028558 |
| *Vireo olivaceus* | KBARG154-07 | MACN-Or-ct3061 | FJ028559 |
| *Vireo olivaceus* | KBARG297-07 | MACN-Or-ct2885 | FJ028560 |
| *Vireo olivaceus* | KBAR205-06 | MACN-Or-ct1121 | FJ028561 |
| *Volatinia jacarina* | KBAR588-06 | MACN-Or-ct1548 | FJ028562 |
| *Volatinia jacarina* | KAARG112-07 | MACN-Or-ct1945 | FJ028563 |
| *Volatinia jacarina* | KBAR590-06 | MACN-Or-ct1550 | FJ028564 |
| *Volatinia jacarina* | KBAR820-06 | MACN-Or-ct1565 | FJ028565 |
| *Xenops minutus* | KBARG367-07 | MACN-Or-ct2931 | FJ028566 |
| *Xenops minutus* | KBARG100-07 | MACN-Or-ct2060 | FJ028567 |
| *Xenops minutus* | KBARG359-07 | MACN-Or-ct2930 | FJ028568 |
| *Xiphocolaptes albicollis* | KBARG401-07 | MACN-Or-ct2956 | FJ028569 |
| *Xiphocolaptes albicollis* | KAARG379-07 | MACN-Or-ct2058 | FJ028570 |
| *Xiphocolaptes major* | KBAR088-06 | MACN-Or-ct793 | FJ028571 |
| *Xiphocolaptes major* | KBAR087-06 | MACN-Or-ct1000 | FJ028572 |
| *Xiphocolaptes major* | KAARG612-07 | MACN-Or-ct3245 | FJ028573 |
| *Xiphorhynchus fuscus* | KBARG404-07 | MACN-Or-ct2971 | FJ028574 |
| *Xiphorhynchus fuscus* | KBARG171-07 | MACN-Or-ct2021 | FJ028575 |
| *Xiphorhynchus fuscus* | KAARG535-07 | MACN-Or-ct2908 | FJ028576 |
| *Xolmis cinereus* | KBAR848-06 | MACN-Or-ct1605 | FJ028577 |
| *Xolmis cinereus* | KAARG292-07 | MACN-Or-ct2391 | FJ028578 |
| *Xolmis cinereus* | KAARG296-07 | MACN-Or-ct2405 | FJ028579 |
| *Xolmis coronatus* | KBARG233-07 | MACN-Or-ct2781 | FJ028580 |
| *Xolmis coronatus* | KBARG241-07 | MACN-Or-ct2782 | FJ028581 |
| *Xolmis irupero* | KAARG158-07 | MACN-Or-ct2124 | FJ028582 |
| *Xolmis irupero* | KBARG150-07 | MACN-Or-ct1827 | FJ028583 |
| *Xolmis irupero* | KBAR534-06 | MACN-Or-ct1470 | FJ028584 |
| *Xolmis irupero* | KBAR576-06 | MACN-Or-ct1532 | FJ028585 |
| *Xolmis irupero* | KAARG207-07 | MACN-Or-ct2178 | FJ028586 |
| *Xolmis pyrope* | KBARG133-07 | MACN-Or-ct2573 | FJ028587 |
| *Xolmis pyrope* | KBARG157-07 | MACN-Or-ct2581 | FJ028588 |
| *Xolmis rubetra* | KBARG325-07 | MACN-Or-ct2826 | FJ028589 |
| *Xolmis rubetra* | KBARG341-07 | MACN-Or-ct2828 | FJ028590 |
| *Zenaida auriculata* | KBARG006-07 | MACN-Or-ct398 | FJ028591 |
| *Zenaida auriculata* | KBARG202-07 | MACN-Or-ct2770 | FJ028592 |
| *Zenaida auriculata* | KBAR444-06 | MACN-Or-ct655 | FJ028593 |
| *Zenaida auriculata* | KBAR446-06 | MACN-Or-ct679 | FJ028594 |
| *Zenaida auriculata* | KBAR568-06 | MACN-Or-ct1522 | FJ028595 |
| *Zenaida auriculata* | KBAR569-06 | MACN-Or-ct1523 | FJ028596 |
| *Zenaida auriculata* | KBAR589-06 | MACN-Or-ct1549 | FJ028597 |
| *Zenaida auriculata* | KAARG302-07 | MACN-Or-ct2694 | FJ028598 |
| *Zonotrichia capensis* | KBARG232-07 | MACN-Or-ct2757 | FJ028599 |
| *Zonotrichia capensis* | KAARG262-07 | MACN-Or-ct2334 | FJ028600 |
| *Zonotrichia capensis* | KAARG553-07 | MACN-Or-ct3166 | FJ028601 |
| *Zonotrichia capensis* | KBAR294-06 | MACN-Or-ct1093 | FJ028602 |
| *Zonotrichia capensis* | KBAR308-06 | MACN-Or-ct671 | FJ028603 |
| *Zonotrichia capensis* | KBAR312-06 | MACN-Or-ct752 | FJ028604 |
| *Zonotrichia capensis* | KBAR609-06 | MACN-Or-ct051 | FJ028605 |
| *Zonotrichia capensis* | KBAR795-06 | MACN-Or-ct1480 | FJ028606 |
| *Zonotrichia capensis* | KAARG565-07 | MACN-Or-ct3179 | FJ028607 |
